# Characterization of the somatostatin system in tilapia: implications for growth and reproduction

**DOI:** 10.3389/fendo.2024.1302672

**Published:** 2024-06-21

**Authors:** Naama Mizrahi, Lian Hollander-Cohen, Ishwar Atre, Miriam Shulman, Aurora Campo, Berta Levavi-Sivan

**Affiliations:** ^1^ Department of Animal Sciences, The Robert H. Smith Faculty of Agriculture, Food, and Environment, Hebrew University of Jerusalem, Rehovot, Israel; ^2^ Department of Poultry and Aquaculture, Institute of Animal Sciences, Agricultural Research Organization, Rishon LeZion, Israel

**Keywords:** LH, FSH, GH, fish, pituitary

## Abstract

Somatostatin (SST) plays diverse physiological roles in vertebrates, particularly in regulating growth hormone secretion from the pituitary. While the function of SST as a neuromodulator has been studied extensively, its role in fish and mammalian reproduction remains poorly understood. To address this gap, we investigated the involvement of the somatostatin system in the regulation of growth and reproductive hormones in tilapia. RNA sequencing of mature tilapia brain tissue revealed the presence of three SST peptides: SST6, SST3, and low levels of SST1. Four different isoforms of the somatostatin receptor (SSTR) subfamily were also identified in the tilapia genome. Phylogenetic and synteny analysis identified tiSSTR2-like as the root of the tree, forming two mega clades, with SSTR1 and SSTR4 in one and SSTR2a, SSTR3a, and SSTR5b in the other. Interestingly, the tiSSTR-5 isoforms 5x1, 5x2, and 5x3 were encoded in the *sstr3b* gene and were an artifact of misperception in the nomenclature in the database. RNA-seq of separated pituitary cell populations showed that SSTRs were expressed in gonadotrophs, with *sstr3a* enriched in luteinizing hormone (LH) cells and *sstr3b* significantly enriched in follicle-stimulating hormone (FSH) cells. Notably, cyclosomatostatin, an SSTR antagonist, induced cAMP activity in all SSTRs, with SSTR3a displaying the highest response, whereas octreotide, an SSTR agonist, showed a binding profile like that observed in human receptors. Binding site analysis of tiSSTRs from tilapia pituitary cells revealed the presence of canonical binding sites characteristic of peptide-binding class A G-protein-coupled receptors. Based on these findings, we explored the effect of somatostatin on gonadotropin release from the pituitary *in vivo*. Whereas cyclosomatostatin increased LH and FSH plasma levels at 2 h post-injection, octreotide decreased FSH levels after 2 h, but the LH levels remained unaffected. Overall, our findings provide important insights into the somatostatin system and its mechanisms of action, indicating a potential role in regulating growth and reproductive hormones. Further studies of the complex interplay between SST, its receptors, and reproductive hormones may advance reproductive control and management in cultured populations.

## 1 Introduction

The two fundamental biological processes of energy homeostasis and reproduction are intimately related. As reproduction is an energy-intensive process, the activities of the regulators of these processes must be tightly coordinated (1). Both energy balance and reproduction are regulated by somatostatin, a critical 14 amino acid peptide hormone first isolated in 1973 from the sheep hypothalamus (2). In addition to the hypothalamus, somatostatin is produced in the gastrointestinal tract, liver, pancreas, and other tissues in all vertebrates, including fish. Somatostatin has many physiological effects, including inhibition of the secretion of growth hormone (GH), prolactin, and gonadotropins from the pituitary gland (3). Negative control of GH by somatostatin has been shown *in vivo* and *in vitro* in several fish species, including salmon, goldfish, rainbow trout, and tilapia (4–7). In the mammal brain, somatostatin also acts as a neuromodulator, regulating motor activity (8, 9), probably by affecting dopaminergic systems (10), and recent evidence suggests it also has significant brain neuroprotective effects (11). Somatostatin also influences the reproductive axis by inhibiting the secretion of luteinizing hormone (LH) from the pituitary (12) and reducing gonadotropin hormone-releasing hormone (GnRH) activity in goldfish, common carp, and grass carp [reviewed in (13)].

The diverse functions of somatostatin are reflected in the complexity of the somatostatin receptor (SSTR) family and their peptide ligands. The development of SSTs and their receptors, like many other neuropeptides and their corresponding receptors, was influenced by various rounds of whole-genome duplication (14). In vertebrates, six paralogous genes of SSTR have been identified (SSTR1–6) (15); five of them also exist in medaka, stickleback, and takifugu (16) and even in cartilaginous fishes (14). SST initiates the inhibition of pituitary hormone secretion by activating G protein-coupled receptors (GPCRs), which trigger a cascade of adenyl cyclase inhibition as well as reductions in intracellular cAMP, protein kinase A (PKA) activity, and Ca^2+^ channel function, while K^+^ channels are activated (17, 18). Some SSTRs reported in trout and goldfish show ligand selectivity (18), while activation of SSTR2a in goldfish has been linked to inhibition of GH release (19).

Vertebrate somatostatin genes group into six distinct clades when subjected to phylogenetic analysis. The somatostatin 1 (SST1) gene is ubiquitous across all vertebrate classes, spanning from agnathans to mammals, and it has served as the progenitor for SST2 and SST5 through two rounds of genome duplications (2R). SST4, an SST1 paralog, emerged from a third genome duplication (3R) observed in most teleost fish. SST3 and SST6 arose from tandem duplications of SST1 and SST2 (16, 20).

The involvement of the SST/SSTR system in GH production and release has been well studied; however, little is known about its effect on reproduction, especially in gonadotropin-producing cells. We have recently used transgenic tilapia with fluorescent-labeled gonadotrophs to perform RNA-seq on specific populations of LH and FSH cells. Our analysis revealed new candidates, including somatostatin receptors, that may directly regulate these hormones (21). In the present study, we investigated the influence of somatostatin on the regulation of gonadotropins in tilapia. RNA-seq of mature tilapia brain tissue revealed three SST peptides and nine SSTR subtypes, including receptors that were enriched specifically in either LH or FSH cells. We then explored the effect of an SSTR agonist and antagonist on SSTRs to analyze the binding sites. We also analyzed the *in vivo* effects of SST on LH and FSH release. Our findings provide valuable insights into the role of somatostatin in the mechanisms governing fish reproduction and may guide the development of novel approaches for reproductive control in fish populations.

## 2 Materials and methods

### 2.1 Animals

Sexually mature Nile tilapia (*Oreochromis niloticus;* body weight, 89.29 ± 32.93 g) were kept and bred in the fish facility unit at the Hebrew University of Jerusalem in 500 L tanks at 26°C, with 14L:10D photoperiod. Fish were fed daily with commercial fish pellets (Raanan Fish Feed, Miluot, Israel). The Gonadosomatic Index (GSI), which was calculated as gonad weight/body weight × 100, was 0.24 ± 0.40%. All experimental procedures were approved by the Hebrew University administrative panel for laboratory animal care.

### 2.2 RNA-seq for SST receptors and ligands

The RNA-seq library and gene expression were previously published (21, 22). To conduct the LH and FSH cell RNA-seq, pituitaries from 20 mature male and 20 mature female transgenic-*O. niloticus* (tg(FSH: GFP; LH: RFP) were harvested and validated using fluorescence microscopy for GFP (FSH)- and RFP (LH)-labeled cells (23, 24). The pituitaries were digested with trypsin into single-cell suspensions according to Biran et al. (25) and Levavi-Sivan and Yaron (26). The cell suspensions were sorted using a FACS Aria III sorter and 488 nm and 561 nm lasers to excite the GFP- and RFP-labeled cells, respectively. Three fractions were collected: a GFP-positive fraction enriched in FSH cells, an RFP-positive fraction enriched in LH cells, and a negative fraction consisting of all the pituitary cells except LH and FSH cells. After sorting, the cells were immediately centrifuged, and total RNA was extracted using TRizol reagent (Thermo Fisher), according to the manufacturer’s instructions. The total RNA samples were sent for RNA-seq library preparation and sequencing.

Due to the low amount of RNA extracted from the sorted cells, RNA libraries were prepared using the SMARTer^®^ Stranded Total RNA-seq Kit v2- Pico Input Mammalian (Takara Bio, Mountain View, CA, USA), which is adaptable for low-quality RNA samples. The libraries were subjected to next-generation sequencing using Illumina^®^ NextSeq^®^ 500 system (Illumina, Inc., San Diego, CA, USA). In the FACS-sorted cells, each library contained at least 24 M reads. Of the total identified genes in the RNA-seq libraries, an average of 45% were uniquely mapped reads to the *O. niloticus* genome (assembly O._niloticus_UMD_NMBU GCA_001858045.3). The average number of reads that were assigned to known genes in the *O. niloticus* genome were 12.3 M reads for LH cells, 2.85 M reads for FSH cells, and 3.5 M reads for negative cells. The FASTQ files and the results of DESeq analysis discussed in this study are available on the National Center for Biotechnology Information (NCBI) Gene Expression Omnibus (GEO) through accession number GSE159470. Cell-specific expression was identified by analyzing each LH or FSH library against the negative library. More than 3K genes with normalized read counts greater than 100 were identified.

For RNA-seq of the brain, complete brain tissues were collected from six mature tilapia. The tissues were transferred directly into TRIzol reagent (Thermo Fisher), and total RNA isolation was immediately performed using miRNeasy Mini Kit (QIAGEN) according to the manufacturer’s instructions. RNA quality and quantity were verified using the 2100 Bioanalyzer instrument (Agilent Technologies), and only samples with RIN. 8 were processed. Total RNA samples were sent to the Technion Genome Center (Haifa, Israel), where they were prepared for sequencing using the TruSeq RNA Sample Preparation Kit v2 (Illumina, San Diego, CA, USA) and subjected to next-generation sequencing using an Illumina Genome Analyzer (HighSeq 2500; Illumina, San Diego, CA, USA), which performed 100 bp single-end read sequencing. The brain RNA-seq samples contained between 18 and 32 million aligned reads (to the *O. niloticus* genome) in each sample; of those, more than 82% were assigned to a known gene. An average of 24.95 M reads were assigned to known genes. FASTQ files and the results of the DESeq analysis discussed in this study are available on the National Center for Biotechnology Information (NCBI) Gene Expression Omnibus (GEO) through accession number GSE169272.

### 2.3 Genome mining and synteny analysis

Genomic and synteny analyses were performed on the studied receptors of Nile tilapia and their duplicates using Genomics v. 110 and the ENSEMBL genome annotations. The analyses were performed on the syntenic regions of the actinopterigyan genes *sstr2*, *sstr3* (named *sstr5a* in spotted gar), and *sstr5* found in the holostean spotted gar (*Lepisosteus oculatus*). In the analysis, we included the genes encoding the proteins of interest in this study, as described in **Table 1**. The analysis used the neighboring genes of the spotted gar as a reference for the whole genome duplication event, also known as 3R, in teleosts. The genes of the duplicated paralogons, paralogons A and B, have been studied in the Asian bonytongue (*Sclreopages formosus*), European seabass (*Dicentrarchus labrax*), Zebrafish (*Danio rerio*), Fugu (*Takifugu rubripes*), and Nile tilapia (*Oreochromis niloticus*).

**Table 1:** Somatostatin receptors of Nile tilapia and the corresponding ligands addressed in this article.

Receptors					
Gene name	Accession	Protein	Paralogon	Protein name in the article	Uniprot
sstr2a	ENSONIG00000019645	XP_003438730.1	A	SSTR2a	I3KUD5
sstr3a	ENSONIG00000013851	XP_019215873.1	A	SSTR3a	I3K8H1
sstr3b	ENSONIG00000021451	XP_019213185.1	B	SSTR3b	I3L056
sstr5b	ENSONIG00000002138	XP_003452896.1	B	SSTR5b	I3J1C6
sstr2b	ENSONIG00000021384	XP_005454973.1	B	Not in this study	A0A669ERT6
sstr5a	ENSONIG00000017693	XP_003455841.1	A	Not in this study	I3KMG5

Ligands					
Gene Name		Symbol_resDvsS	Protein	Protein name in the article	
Somatostatin-1B		LOC100698045	XP_003444846.1	SST6	
Somatostatin-2		LOC100694069	XP_003448989.2	SST3	
Somatostatin- 1		LOC100693797	XM_003448940.5	SST1	

### 2.4 Phylogenetic tree

The phylogenetic tree was inferred using the maximum likelihood method and JTT matrix-based model (27). The analysis involved 153 amino acid sequences and 780 distinct alignment patterns. The tree with the Final ML Optimization Likelihood (-44204.62) is shown. Initial trees for the heuristic search were obtained automatically by applying the Neighbor-Join and BioNJ algorithms to a matrix of pairwise distances estimated using the JTT model with 1000 bootstrap replicates and then selecting the topology with a superior log likelihood value (28).

### 2.5 Homology modeling and binding site prediction

Three-dimensional *in silico* models for tilapia SSTRs (tiSSTR2a, tiSSTR3a, tiSSTR5b, and tiSSTR3b) and SSTs (tiSST6 and tiSST3) were prepared using the I-TASSER server (29, 30) and human (hu)SSTR2-huSST-14 (PDB:7T10) (31) as a template. The 3D models were selAll receptors in teleosts exhibit a monophyletected based on structural stability, C-score, and structural similarity with the known huSSTR2 structure (PDB: 7T10). Further structure processing, binding site prediction, docking, and mutation analysis were performed using Schrödinger (BioLuminate, Schrödinger, LLC, New York, NY, 2021). These structures were further refined and used for binding-site predictions.

### 2.6 *In situ* hybridization chain reaction and immunofluorescence on double-labeled pituitary tissues

Transgenic tilapia [FSH:GFP and LH:RFP (23, 24)] were employed for the HCR and immunofluorescence assays. Fish were anesthetized with MS-222 (Sigma) and decapitated. The pituitary glands were removed and fixed with 4% (wt/vol) paraformaldehyde in PBS for 6 h at 4°C, and then immersed in phosphate-buffered saline (PBS) containing 20% (wt/vol) sucrose and 30% (vol/vol) optimal cutting temperature (OCT) (Sakura) for about 24 h. The pituitaries were then embedded in OCT, frozen in liquid nitrogen, sectioned frontally at 12 μm on a cryostat at -18°C, and mounted onto Superfrost™ Plus glass slides (Thermo Scientific). All samples were kept at -80°C.

The HCR protocol was adapted from Molecular Instruments HCR v3.0 for fresh frozen or fixed frozen tissue sections, as described by Choi et al. (32), with slight modifications. Briefly, frozen sections were thawed to room temperature and then fixed in ice-cold 4% (wt/vol) paraformaldehyde in PBS for 15 min at 4°C. The pituitaries were then immersed in different ethanol concentrations (50, 70, and 100%) for 5 min each at room temperature. Each section was then incubated with 200 µl of 10 µg/ml proteinase K solution for 10 min in a humid chamber at 37°C and prehybridized in the probe hybridization buffer for 10 min at 37°C. The slides were incubated overnight at 37°C in a humid chamber in the same solution containing 0.4–0.8 pmol of denaturation probes (designed specifically for each tilapia SST receptor by Molecular Instruments; lot numbers SSTR2a-B1, PRJ208; SSTR5b-B1, PRJ209; SSTR3a-B1, PRJ210; SSTR3b-B1, and PRJ211). After hybridization, the sections were washed in 75% probe wash buffer/25% 5xSSCT for 15 min at 37°C, followed by a second wash in 50%, a third wash in 25% probe wash buffer, and a final wash with only 5×SSCT solution. The slides were preamplified with an amplification buffer for 30 min at room temperature. The pituitary slides were incubated overnight in a dark chamber in the same buffer containing snap-cooled h1 and h2 hairpins. To remove excess hairpins, the slides were washed in 5×SSCT twice for 30 min and then for 5 min.

After the HCR, the sections were subjected to immunofluorescence labeling. Sections were blocked in 5% (v/v) normal goat serum with 0.3% (v/v) Triton X-100 for 1 h at room temperature and incubated with specific antibodies raised in rabbits against recombinant tilapia rtGH (7, 33), diluted 1:500 in antibody dilution buffer (1% w/v BSA; 0.3% Triton X-100 in PBS) overnight at 4°C. The samples were stained using secondary antirabbit antibodies conjugated to Alexa fluorescent dyes (Invitrogen) diluted 1:300 and incubated for 2 h at room temperature. Following staining, the slides were stained with 4′,6-diamidino-2-phenylindole (DAPI), washed, and mounted using an antifade solution (2% w/v propyl-gallate, 75% v/v glycerol in PBS). The pituitaries were imaged using a confocal fluorescence microscope (Leica microsystems) using ×20 and ×60 objectives, and images were processed using the Fiji program (34).

### 2.7 Somatostatin peptide synthesis and purification

Tilapia (ti) somatostatin 6 (tiSST6; (N) AP**C**KNFFWKTFTS**C** (C); accession no. XP_003444846.1) and somatostatin 3 (tiSST3; (N) AG**C**KNFYWKGLTS**C** (C) accession no. XP_003448989.2) were synthesized by GL Biochem (cysteines are indicated in bold) using an automated solid-phase method and applying Fmoc active-ester chemistry. The crude peptides were purified by HPLC to >95% purity. The pure peptides had a single peak in analytical RP-HPLC, with the expected mass determined by MS analysis. For signal transduction reporter assays, the peptides were dissolved to the desired concentration in double-distilled water.

### 2.8 Receptor signal transduction reporter assays

The signaling pathways of SSTRs were studied by inserting the entire coding sequence of the four receptors expressed in the pituitary (SSTR2a, SSTR3a, SSTR5b, and SSTR3b; accession nos. XP_003438730.1, XP_019215873.1, XP_003452896.1, and XP_019213185.1, respectively; **Table 1**) into pcDNA3.1 (Invitrogen) and verified by cloning and sequencing. The sequences were obtained from GenScript Biotech based on sequence information retrieved from GenBank. The procedures for transient transfection of the different cell lines and receptor stimulation have been described previously (35, 36). In brief, COS-7 cells were cotransfected with a luciferase reporter plasmid (Cre-luc; 3 µg) and one of the SSTRs (3 µg). As a control treatment, the receptors were transfected without reporter plasmid (data not shown). After 48 h, the transfected cells were exposed to increasing concentrations of the tilapia native peptides SST6 and SST3 in the presence of forskolin (FSK; 20 μM; Sigma-Aldrich), an activator of protein kinase A that increases cAMP production. Six hours after stimulation, the cells were analyzed using the GloMax multidetection system (Promega).

In another set of assays, the SST receptors were exposed to increasing concentrations of cyclosomatostatin, an SSTR antagonist (0–1000 nM; each in triplicate), in combination with octreotide, an SSTR agonist (10 nM), and FSK (20 μM), or increasing concentrations of octreotide (0–1000 nM; each in triplicate) in combination with cyclosomatostatin (10 nM) and FSK (20 μM). Three individual experiments were conducted using distinct batches of COS-7 cells for each experiment and including three replicates for every concentration.

### 2.9 *In vivo* experiment

Adult female tilapia (body weight 89.29 ± 32.93 g) were injected IP with saline, cyclosomatostatin, or octreotide (100 µg/kg; n = 8 fish per group). The fish were bled from the caudal blood vessels into heparinized syringes at 0, 2, 4, 6, and 24 h after injection. Blood was centrifuged at 3,200 rpm for 30 min at 4°C to obtain plasma samples, which were stored at −20°C until assayed. This standard protocol was used previously to test the effect of GnRH and other hypothalamic neuropeptides on circulating levels of LH, FSH, and GH in tilapia (33, 37, 38). Three independent experiments were performed for each treatment.

### 2.10 ELISA for the measurement of tilapia FSH, LH, and GH

Plasma levels of LH, FSH, and GH were measured by specific competitive ELISAs developed for tilapia (39, 40) based on recombinant (r)tiGTHs or rtiGH. The isera were produced against rtLHβ (41), rtFSHβ (40), or rtGH (39), and rtLHβα (41), rtFSHβα (37) or rtGH (39) was used to generate a standard curve. The sensitivity of plasma measurements was 15.84 pg/ml for LH, 0.24 pg/ml for FSH, and 35.0 pg/ml for GH. The inter-assay coefficients of variation (CV) were 14.8, 12.5, and 13%, whereas intra-assay CVs were 7.2, 8, and 8% for LH, FSH, and GH, respectively.

### 2.11 Statistical analysis

The results are presented as mean ± SEM. Two-way ANOVA was used to compare mean LH, FSH, and GH values from the *in vivo* experiments. One-way ANOVA was used to compare the signal transduction results. In cases of statistically significant differences between the groups, the analysis was followed by an a posteriori Tukey multiple comparison test using JMP software version 9 (SAS Institute, Inc., Cary, NC, USA).

## 3 Results

### 3.1 Phylogenetic and synteny analyses of SSTRs and their differential expression in tilapia pituitary

**Figure 1:**
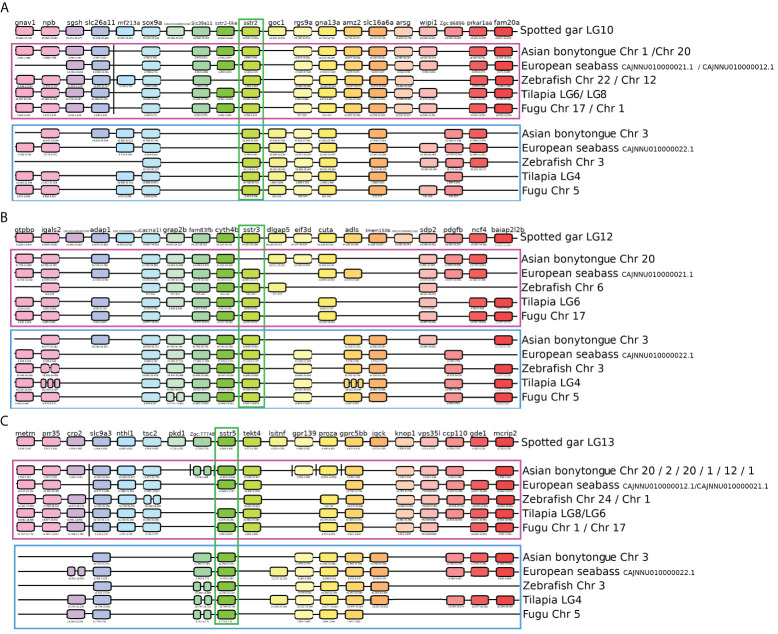
Synteny analysis of *sstr* genes. Representation of the genomic regions of the studied genes. Gene positions are expressed in 10^3^. **(A)** Synteny analysis of the *sstr2* gene. The spotted gar (*Lepisosteus occulatus*) has been used as a reference to identify two duplicated paralogons in teleosts. Paralogon A is enclosed in the purple square and paralogon B is enclosed in the blue square. The gene of interest, *sstr2*, is marked in green. **(B)** Synteny analysis of the *sstr3* gene. The spotted gar (*Lepisosteus occulatus*) has been used as a reference to identify two duplicated paralogons in teleosts. Paralogon A is enclosed in the purple square and paralogon B is enclosed in the blue square. The gene of interest, *sstr3*, is marked in green. **(C)** Synteny analysis of the *sstr5* gene. The spotted gar (*Lepisosteus occulatus*) has been used as a reference to identify two duplicated paralogons in teleosts. Paralogon A is enclosed in the purple square, and paralogon B is enclosed in the blue square. The gene of interest, *sstr5*, is marked in green.

In order to comprehend the involvement of somatostatin in the reproductive processes of fish, we initially undertook a thorough investigation of the tilapia genome, conducting analyses of phylogenetics and synteny related to their receptors. Genome mining revealed that the studied receptors in teleost fish were encoded in three groups of preserved *sstr* genes: *sstr2*, *sstr3*, and *sstr5*. The syntenic analysis showed that the *sstr2*, *sstr3*, and *sstr5* genes in spotted gar were duplicated in teleosts due to a whole genome duplication event. Two paralogons, A and B, containing an asymmetric conservation of the genes, were identified in each species (**Figure 1**). The duplicated genes in teleosts were differentiated after the paralogon as *sstr2a*, *sstr2b*, *sstr3a*, *sstr3b*, *sstr5a*, and *sstr5b*. The paralogon A of the gene *sstr2* contains the genes *fam20*, *amz2*, *arsg, slc39a1*, *sstr2-like*, and *sgsh*. Paralogon A had also undergone a transposition to a different chromosome in all the studied teleosts between the genes *slc26a11* and *rnf213a* (**Figure 1A**; **Supplementary Table 1A**). Paralogon B was characterized by containing the genes *goc1* and *zgc:86896*. The protein SSTR2 was encoded in the gene *sstr2a* of the Nile tilapia found in paralogon A (**Table 1**).

The gene related to *sstr2*, named *sstr2-*like, was found in the spotted gar as the closest gene to *sstr2*. In Nile tilapia, the gene *sstr2-like* consists of one exon 1963 nucleotides in length that encodes a protein of 339 amino acids. The protein encoded by the *sstr2-like* gene has prints for a somatostatin receptor. However, unlike the other SST receptors, the protein encoded by the *sstr2-like* gene contains only 6 transmembrane domains; it is missing one domain. This gene was also affected by the whole genome duplication effect and has been preserved in paralogon A in the teleosts.

The gene encoding the SSTR6 receptor exists in medaka (*Oryzias latipes*) and likely emerged after loss of a duplicate during the 3R event (42). Although not reported previously, medaka lacks copies of the sstr6, sstr1, and sstr4 genes. By contrast, tilapia lacks the sstr6, sstr1, and sstr4 genes altogether. These discrepancies in gene conservation between Nile tilapia and medaka may result from extensive genomic rearrangements that have affected the distribution of somatostatin receptor genes in teleosts. Chromosome missegregation and subsequent genomic rearrangements may also have contributed to these differences due to the short generation time in Nile tilapia and medaka species.

The analysis of the genomic region of *sstr3* revealed that paralogon A contains the gene *cuta* and paralogon B contains the gene *tmemb150b* (**Figure 1B**; **Supplementary Table 1B**). The protein SSTR3a was encoded by the gene *sstr3a* of the Nile tilapia found in paralogon A, and the protein SSTR3b was encoded by the gene *sstr3b* of the Nile tilapia found in paralogon B (**Table 1**). Further analysis of the receptors encoded by the gene *sstr3b* showed that SSTR5X1-X2 and -X3 are apparently three splicing variants of this gene and are misnamed as artifacts of possibly different naming processes.

Paralogon A of the gene *sstr5* contains the genes *metrn, prr35, nthl1, tsc2, text4, knop1*, and *vps35l*. Paralogon B contains the genes *iqck* and litaf. An entire transposition of paralogon A in teleosts occurred between the genes *crp* and *slc9a3* (**Figure 1C**; **Supplementary Table 1C**). The Asian bonytongue also experienced the transposition of some of the genes into different chromosomes. The protein SSTR5b was encoded by the gene *sstr5b* of the Nile tilapia found in paralogon B (**Table 1**).

**Figure 2:**
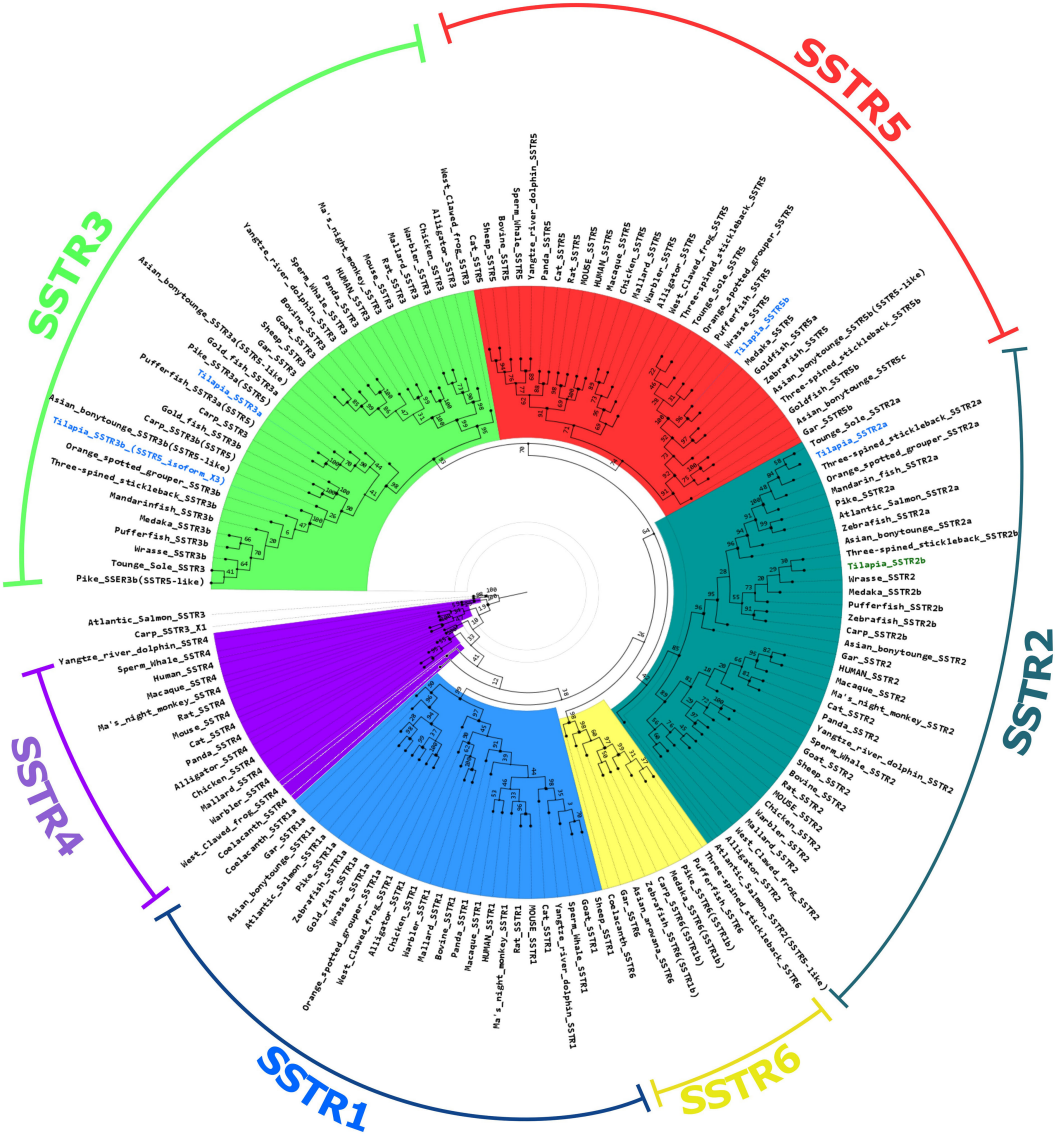
Phylogenetic tree of somatostatin receptors. A maximum likelihood method-based phylogenetic tree of the different SSTR types. Tilapia SSTRs relevant to this study [SSTR2a; SSTR3a; SSTR5b, and SSTR3b(5_isoform_X3)] are marked in blue. The tilapia SSTR2b is marked in green. The scale is marked on the right bottom corner.

The results of the phylogeny analysis showed a major group of monophyletic origin formed by SSTR5, SSTR3, and SSTR2. Within this branch, SSTR3 and SSTR5 exhibit a shared monophyletic origin, and SSTR2 is basal to them. The SSTR4, SSTR1, and SSTR6 sequences are basal to the previous list. Within this grouping, the SSTR4 sequences appear to be basal to SSTR1 and SSTR6 (**Figure 2**; **Supplementary File 1**, **Supplementary Table 2**). Generally, each group of SSTR receptors was further split into two clades, one for tetrapods and the other containing piscine SSTRs. In the piscine group, the spotted gar is basal to the teleosts. In SSTR1 and SSTR6, the coelacanth is basal to the actinopterygians and teleosts, while the coelacanth is absent from the other groups. No actinopterygian or teleost SSTR4 has been found thus far, but it is still present in the sarcopterygians, including the coelacanth.

**Figure 3:**
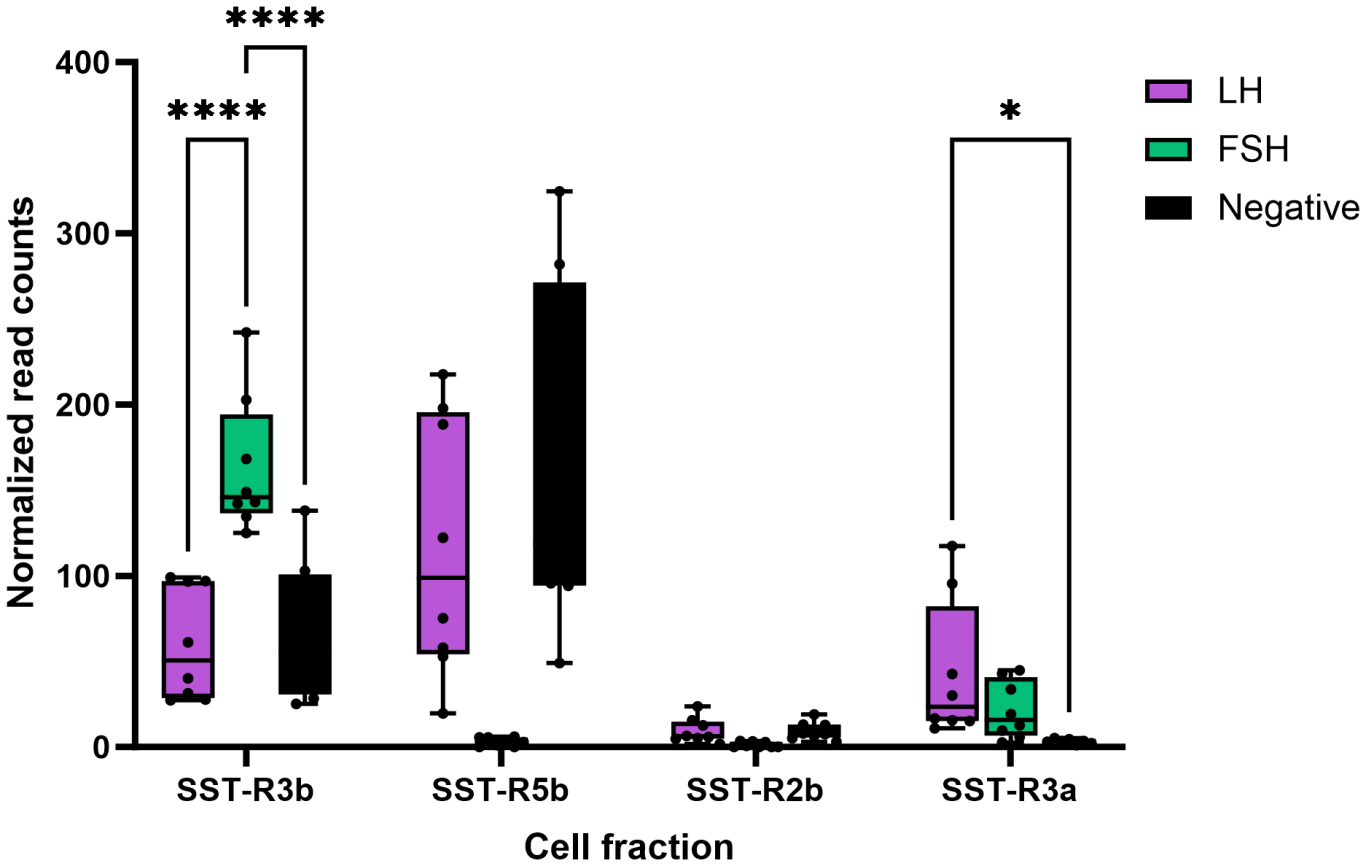
Expression of somatostatin receptors in LH and FSH cells isolated from tilapia pituitaries. RNA-seq of FACS-isolated cells identified the different types of SSTRs from pituitaries of transgenic tilapia expressing RFP in LH cells and GFP in FSH cells. Isolated LH cells, FSH cells, and negative pituitary cells were subjected to RNA-seq as described previously (21). The complete transcriptome information is available in GEO through accession number GSE159470. Asterisk represents significantly different expression values as compared to the negative cells (*p < 0.05; ****p < 0.00001).

We also performed RNA-seq analysis on pituitaries of transgenic tilapia that express GFP in FSH cells and RFP in LH cells (23, 24). Of the nine paralogs of SSTR identified in the tilapia genome, only four were expressed in the pituitary (SSTR3b, SSTR5b, SSTR2a, and SSTR3a; marked in blue in **Figure 2**) (21). Comparison of each receptor expression in LH and FSH cells to their expression in the negative fraction (**Figure 3**) identified two main SSTRs that had significantly high expression in each gonadotroph: *sstr*3b was highly expressed in FSH cells, whereas *sstr*3a and *sstr5b* were highly expressed in LH cells. The *sstr2a* and *sstr5b* genes were expressed in the negative fraction, probably because this fraction included GH cells (**Figure 3**, **Table 2**).

**Table 2:** The relative number of LH, FSH, and GH cells expressing each SSTR subtype according to hybridization chain reaction (HCR)/immunofluorescence assays, as presented in **Figure 4**.

	sst-r2a	sst-r3a	sst-r5b	sst-r3b
**LH**	+	++	–	++
**FSH**	–	+	–	++
**GH**	++	–	++	–

### 3.2 Colocalization of somatostatin receptors in tilapia pituitary

**Figure 4:**
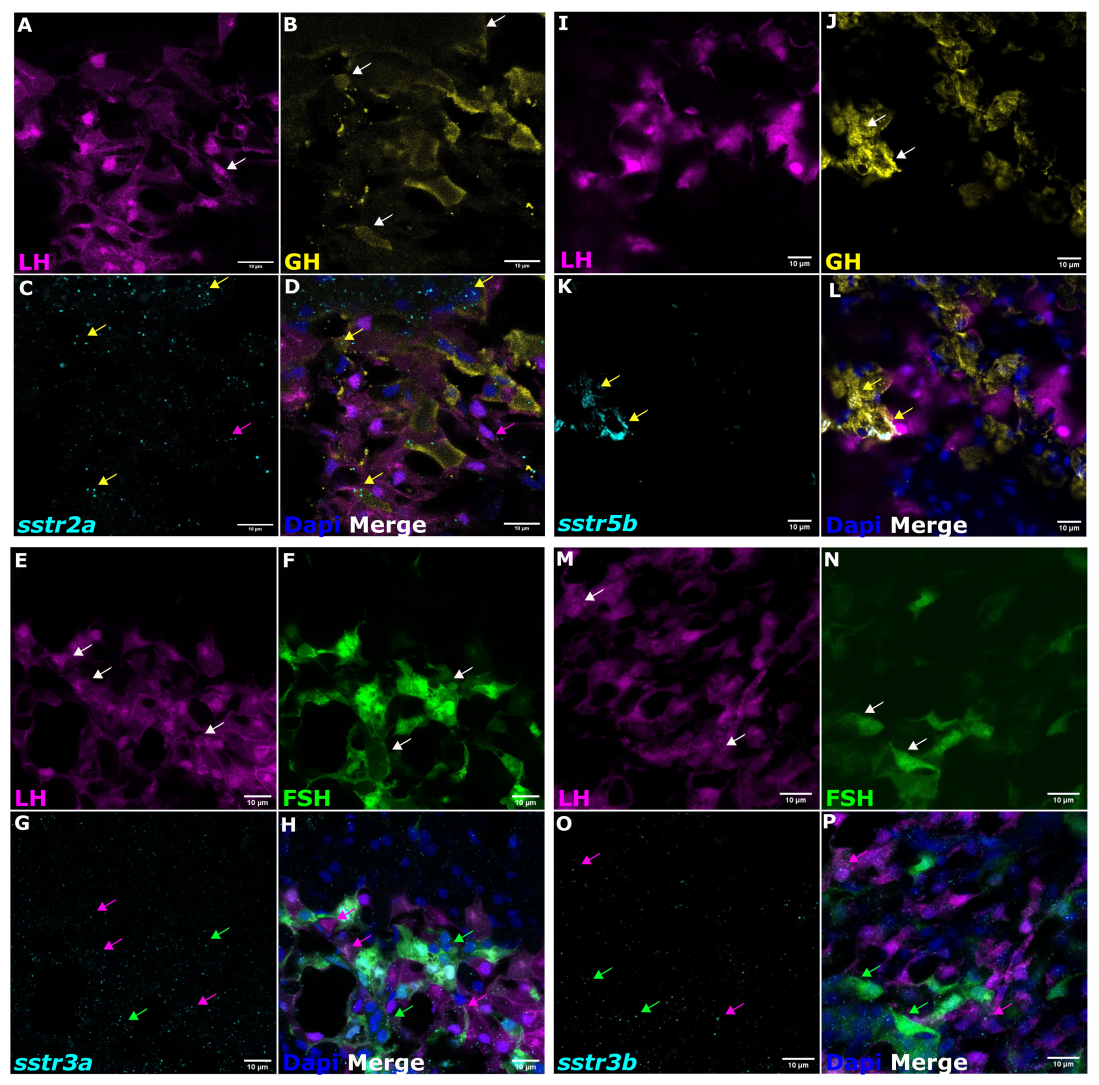
The mRNA expression analysis of *sstr* in tilapia pituitary cells. Double-labeled pituitaries from transgenic tilapia with labeled LH [**(A, I, E, M)** magenta] and FSH cells [**(F, N)** green] and immunofluorescence-stained for GH [**(B, J)** yellow] were used for *in situ* hybridization chain reaction (HCR) (v3.0) to determine co-localization of mRNA expression of different *sstr* genes [**(C)** *sstr2a*; **(K)** *sstr5b*; **(G)** *sstr3a*; **(O)** *sstr3b*; cyan). Nuclei are stained with DAPI [**(D, L, H, P)** blue]. In the merged box, magenta arrows show LH cells, green arrows show FSH, and yellow arrows show GH cells. Scale bar, 10 µm.

Cell-specific SSTR expression was revealed by RNA-seq and *in situ* hybridization using specific probes for each receptor in the double-labeled transgenic fish (23, 24). The use of immunofluorescence with specific antibodies for GH showed that the somatotropes almost exclusively expressed *sstr2a* (**Figures 4A–D**) and *sstr5b* (**Figures 4I–L**) mRNA, whereas *sstr3a* (**Figures 4E–H**) and *sstr3b* (**Figures 4M–P**) mRNA were expressed mainly by FSH and LH cells. These results are summarized in **Table 2**.

### 3.3 Homology modeling and binding site prediction

**Figure 5:**
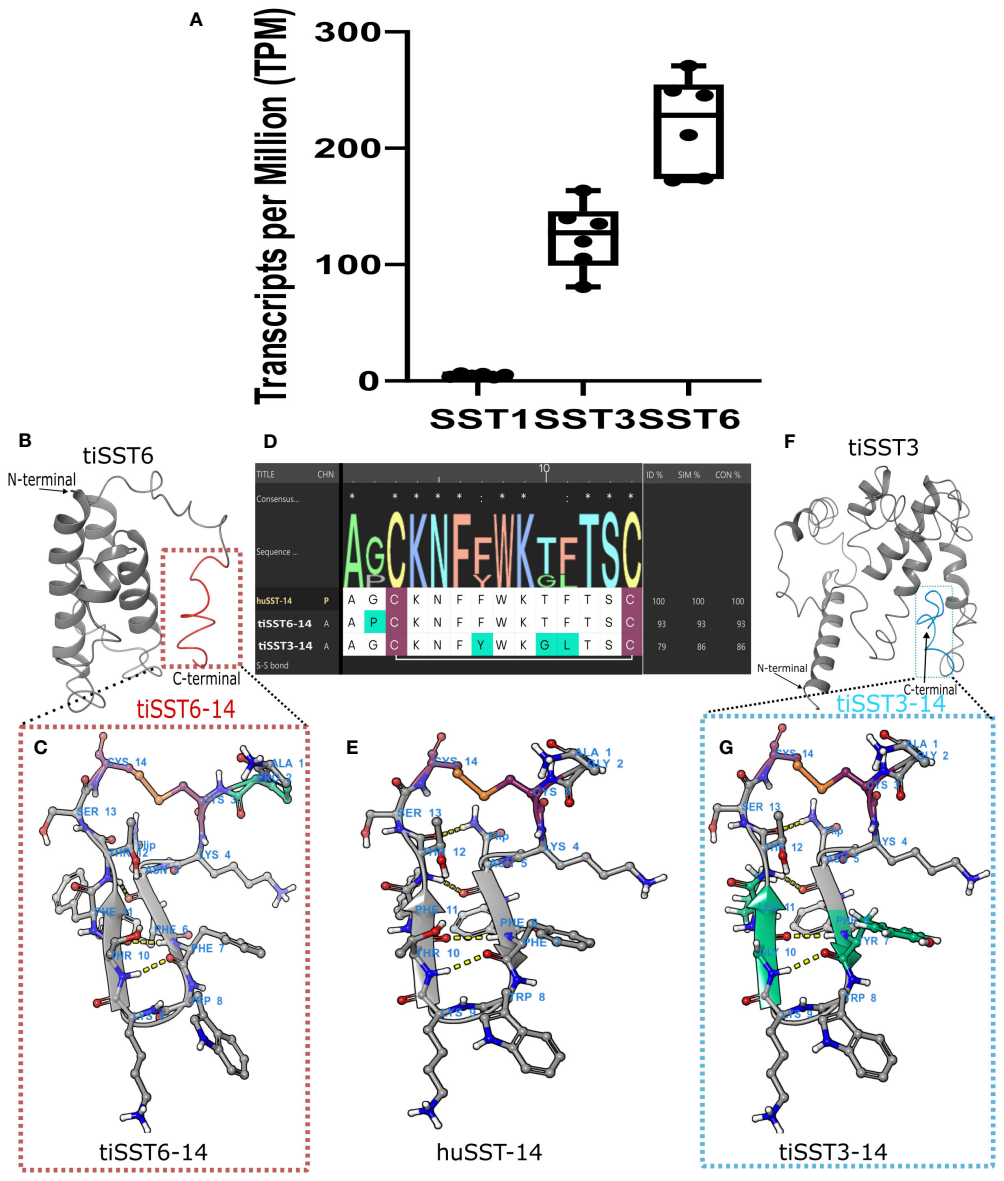
Structure of active peptide SST14 in tilapia brain. The 14-aa peptide SST14 is marked in red in tiSST6 **(A)** and in aqua in tiSST3 **(E)**. Multiple sequence alignment and homology models of SST6–14 [**(B)** APCKNFFWKTFTSC], tiSST3–14; [**(F)** AGCKNFYWKGLTSC]; and huSST14 [**(D)**, AGCKNFFWKTFTSC]. Cystine (Cys) is marked in maroon, sulfide bonds are marked in orange and red, and variations from huSST14 are marked in cyan. **(C)** The weblogo depicts the sequence alignment of the tilapia and human SST1–14. **(G)** The different types of somatostatin that are expressed by RNA-seq of tilapia brain.

RNA-seq conducted on whole brain samples from mature reproductive tilapia fish (22) indicated that tiSST3 and tiSST6 were more highly expressed than SST1 in mature reproductive males (**Figure 5A**, **Table 1**), implying potential pivotal roles for these two subtypes in reproduction. Because we focused on brain SST and its interactions with pituitary-expressed SSTRs during the reproductive season, we conducted further analyses only on tiSST6 and tiSST3.

Somatostatin precursors are large proteins (e.g., SST6, 110 aa; SST3, 191 aa), whereas the known biologically active component SST-14 is only a 14-aa-long peptide at the C-terminus of the protein (**Figures 5B, F**). SST-14 acquired a circular post-cleavage conformation by the formation of a sulfide bond between the cysteines at the 3^rd^ and 14^th^ positions of the peptide, resulting in the creation of a ring structure in the peptide chain (**Figure 5**). tiSST6–14 showed a higher identity to huSST-14, with only a proline substitution for glycine at the 2^nd^ position of the peptide. In tiSST3–14, tyrosine replaced phenylalanine at 7^th^ position, glycine replaced threonine at 10^th^ position, and leucine replaced phenylalanine at the 11^th^ position relative to huSST-14.

**Figure 6:**
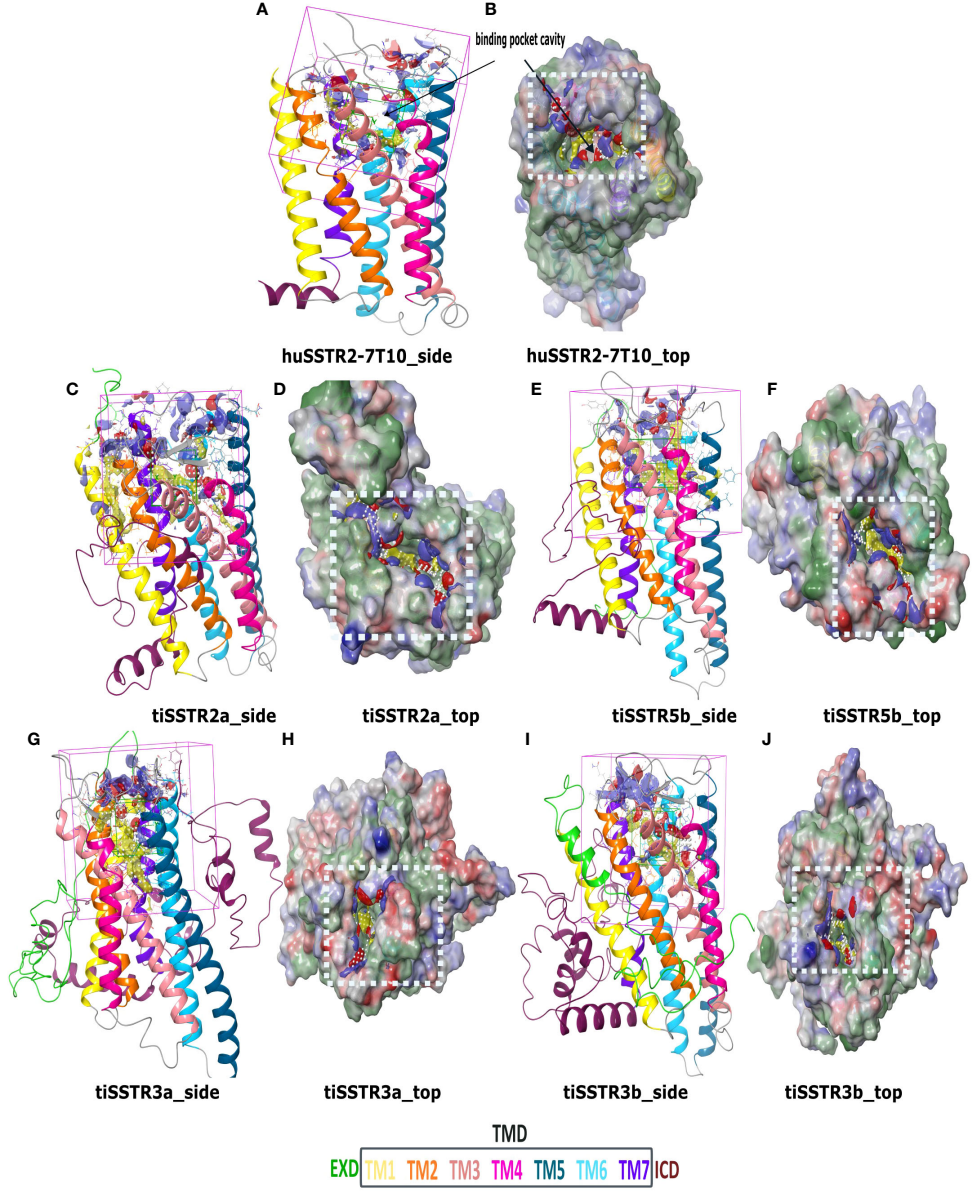
SSTR homology models and binding pockets. Homology models and predicted binding sites for huSSTR2 (PDB:7T10) **(A, B)**; tiSSTR2a **(C, D)**; tiSSTR5b **(E, F)**; tiSSTR3a **(G, H)**; and tiSSTR3b **(I, J)** showing ECD (green), TM1 (yellow), TM2 (orange), TM3 (peach), TM4 (pink), TM5 (teal), TM6 (cyan), TM7 (violet), and ICD (maroon). The site map within the white box **(D, F, H, J)** shows the hydrophobic region (yellow), the hydrogen-bond donor (blue), and acceptor maps (red).

The models for tiSSTRs generated using huSSTR2 as a template (PDB:7T10) are shown in **Figures 6A, B**. The SST receptors are class A (rhodopsin-like) GPCRs (31). As with other peptide-binding receptors, they exhibit an extracellular domain (ECD), a seven-transmembrane domain (7TM) connected by intracellular and extracellular loops (ECLs), and an intracellular domain (ICD). Our models showed a large ECD and ICD in all tiSSTRs; however, tiSSTR3a (482aa) and tiSSTR3b (483aa) had larger sequences and contained structurally similar N-terminal and C-terminal regions, which were longer than in the other homologs.

tiSSTR2a (**Figures 6C, D**), tiSSTR5b (**Figures 6E, F**), tiSSTR3a (**Figures 6G, H**) and tiSSTR3b (**Figures 6I, J**) each possessed a putative orthosteric binding pocket, a common feature in most class A GPCRs (43). This binding pocket was located in the transmembrane cavity, which implies the potential involvement of the ECD and ECL. Unlike the case in other specific GPCRs, such as GnRHRs, the ECD did not obstruct the binding pocket in tiSSTRs (**Figure 6**). The binding sites of tiSSTR3a and tiSSTR3b were quite similar and were primarily composed of the hydrophobic cleft in the TMD. The binding site located on tiSSTR2a was apparently larger than in other SSTRs.

### 3.4 Signal transduction analysis of SSTRs in the presence of a somatostatin agonist and native peptides

**Figure 7:**
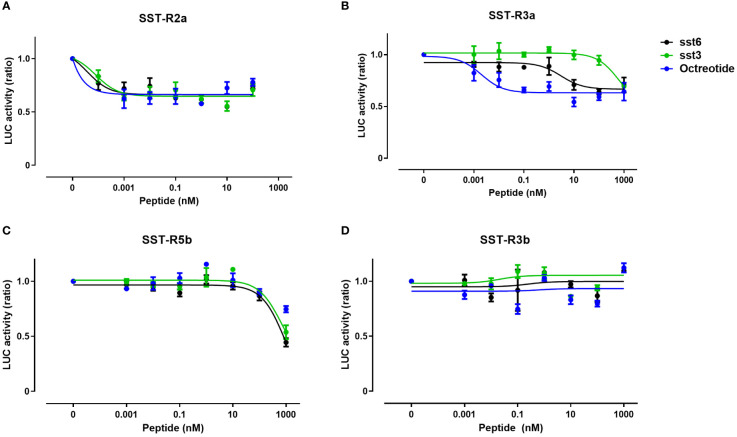
Signal transduction activity of somatostatin receptors induced by an SSTR agonist and native SST peptides. COS‐7 cells were co‐transfected with Cre‐luc plasmid and either SSTR2a **(A)**, SSTR3a **(B)**, SSTR5b **(C)**, or SSTR3b **(D)**. Transfected cells were exposed to increasing concentrations of octreotide (an SST agonist) or somatostatin peptides SST6 or SST3 (0–1000 nM) in combination with FSK (20 μM). Graphs show fold changes in luciferase activity relative to the basal level. Averaged data from three experiments are shown and are presented as mean ± SEM. Asterisk-marked points significantly differ from basal levels: *p < 0.05; **p < 0.01, (one-way ANOVA followed by Dunnett’s test).

Comparison of the activation of the different SST receptor types by the native SST6 and SST3 ligands versus octreotide, a commercial SSTR agonist, in COS-7 cells revealed differences in the potency, selectivity, and signal transduction pathways. Like dopamine and melatonin receptors, SSTRs are inhibitory receptors that inhibit adenylate cyclase activity upon coupling to Gαi/o and reduce cAMP production (17, 36); thus, the ligand effect was analyzed in the presence of forskolin (20 µM), which increased cAMP levels, as reflected by an elevation in luciferase activity. Treatment with octreotide, tiSST6, and tiSST3 at subnanomolar concentrations inhibited tiSSTR2a Cre-luc activity (**Figure 7A**, **Table 3**). Highly potent inhibition of SSTR5b Cre-luc activity was also observed (**Figure 7C**; IC_50_ = 906.1, 720.2, and 157.0 nM, for octreotide, tiSST6, and tiSST3, respectively; **Table 3**). Octreotide was the most efficient activator of SSTR3a (IC_50_ = 0.004 nM; **Figure 7B**, **Table 3**), and SST6 was more efficient than SST3 (**Figure 7B**; IC_50_ = 3.75 and 572.9 nM, respectively). When exposed to SSTR-3b, both ligands inefficiently inhibited Cre-luc activity (**Figure 7D**).

**Table 3:** IC_50_ values (nM) of SSTR agonist octreotide and native peptides SST6 and SST3 on tilapia SST receptors.

	SST6	SST3	Octreotide
**SST-R2a**	3.75x10^-5^ ± 0.03	2.86x10^-5^ ± 0.02	1.09x10^-7^ ± 0.04
**SST-R3a**	3.75 ± 0.04	572.9 ± 0.1	0.004 ± 0.03
**SST-R5b**	906.1 ± 0.5	720.2 ± 0.5	157 ± 0.1
**SST-R3b**	ND	ND	ND

Cre-luciferase was used to assess PKA activation. Results are shown as mean ± SEM.

ND, Not detectable.

### 3.5 Signal transduction activity of a somatostatin agonist (octreotide) and antagonist (cyclosomatostatin)

**Figure 8:**
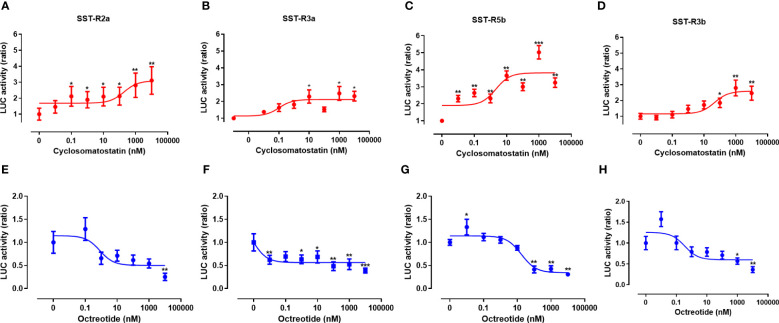
Signal transduction activity of somatostatin receptors induced by an SSTR agonist (octreotide) and antagonist (cyclosomatostatin). COS‐7 cells were co‐transfected with Cre‐luc plasmid and either SSTR2a **(A, E)**, SSTR3a **(B, F)**, SSTR5b **(C, G)**, or SSTR3b **(D, H)**. Transfected cells were exposed to increasing concentrations of cyclosomatostatin (0–1000nM) in the presence of octreotide (10 nM) and FSK (20 μM; **(A–D)**, red lines) and increasing concentrations of octreotide (0–1000nM) in the presence of cyclosomatostatin (10 nM) and FSK (20 μM; **(E–H)**, blue lines). Graphs show fold changes in luciferase activity relative to the basal level. Averaged data from three experiments are shown and are presented as mean ± SEM. Asterisk-marked points significantly differ from basal levels: *p < 0.05; **p < 0.01, ***p< 0.0001 (one-way ANOVA followed by Dunnett’s test).

Agonists and antagonists are commonly used to study receptor activation and to develop drugs with specific targets and activities. We, therefore, activated tiSSTRs by cyclosomatostatin, an SSTR antagonist, dose-dependently in the presence of a constant concentration of SSTR agonist (10 nM; **Figures 8A–D**). Conversely, the effect of different doses of octreotide, an SSTR agonist, was tested in the presence of a constant concentration of SSTR antagonist (10 nM; **Figures 8E–H**). Although both octreotide and cyclosomatostatin were developed for mammalian receptors (44), both were very effective in fish SSTRs (45). SSTR3a and SSTR5b were most effectively stimulated by the SSTR antagonist (EC_50_ = 0.1 and 2.15 nM, respectively; **Figures 8B, C**, **Table 4**). By contrast, SSTR2a displayed the lowest stimulation by different doses of SSTR antagonist (EC_50_ = 188.4 nM, **Figure 8A**, **Table 4**) and the highest inhibition by SSTR agonist together with SSTR3a and SSTR3b (IC_50_ = 0.81, 8.6x10^-4^ and 0.35 nM, **Figures 8F, H**, **Table 4**). SSTR3a was the most effective when stimulated with octreotide (**Figure 8F**, **Table 4**). Overall, SSTR5b was the most efficient receptor in terms of maximal response to the SST antagonist.

**Table 4:** The effect of cyclosomatostatin and octreotide on tilapia SST receptors.

		SST-R2a	SST-R3a	SST-R5b	SST-R3b
**Cyclosomatostatin** (SSTR antagonist)	EC_50_ (nM)	188.90 ± 4.34	0.10± 0.001	2.15 ± 0.009	46.21 ± 3.82
**Octreotide** (SSTR agonist)	IC_50_ (nM)	0.81 ± 0.01	8.6x10^-4^ ± 0.0007	17.36 ± 3.14	0.35 ± 0.04

Cre-luciferase was used to assess PKA activation. Results are shown as mean ± SEM.

### 3.6 *In vivo* effect of SSTR antagonist and agonist on FSH, LH, and GH plasma levels

**Figure 9:**
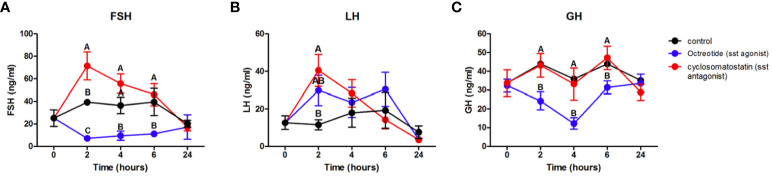
*In vivo* effect of an SSTR agonist (octreotide) and antagonist (cyclosomatostatin) on gonadotropins and GH plasma levels. Female tilapia were injected with the SSTR antagonist cyclosomatostatin or the SSTR agonist octreotide (100 μg/kg BW) at time point 0; saline-injected fish served as controls. Blood was sampled at 2, 4, 6, and 24 h after injection. Plasma FSH **(A)**, LH **(B)**, and GH **(C)** levels were analyzed by specific ELISAs (mean ± SEM; n = 8 fish per group). Letters denote statistically significant differences among groups (*P* < 0.05), as determined by two‐way ANOVA followed by Tukey’s multiple comparison test.

We investigated the physiological impact of the SSTR antagonist and agonist on the release of gonadotropins and growth hormone by assessing their plasma concentrations after administering intraperitoneal injections of these compounds to sexually mature female tilapia. Administration of SSTR antagonist cyclosomatostatin increased FSH and LH levels, compared to the control, as early as 2 h post-injection (**Figures 9A, B**). However, GH levels were the same as in the control fish. As expected, administration of the SSTR agonist octreotide significantly decreased GH plasma levels at 2 h post-injection, and this reduction persisted even after 4 and 6 h (**Figure 9C**). Octreotide also resulted in decreased FSH plasma levels at 2 h postinjection and thereafter (**Figure 9A**), whereas LH plasma levels remained similar to the control (**Figure 9B**).

## 4 Discussion

Somatostatin has diverse physiological functions in all vertebrates, including an essential role in inhibiting GH secretion from the pituitary (2). Its inhibitory action was later shown for a wide range of hypophyseal hormones, including prolactin, thyrotropin, and ACTH (46–48), as well as in the gastrointestinal hormones gastrin (49), cholecystokinin (CCK) (50), gastric inhibitory peptide (GIP) (51), neurotensin (52), and pancreatic glucagon (53, 54) and insulin (51, 53, 54). SST is also a neuromodulator that regulates motor activity (55) and aggressive behavior in cichlid fish (45). However, little is known about the effect of SST on reproduction in either fish or mammals. Therefore, we studied the regulation of growth and reproduction hormones by the somatostatin system in tilapia.

In jawed vertebrates, SST1–6 are the products of at least six paralogous *sst1–6* genes (16, 56). Following the second whole genome duplication, the vertebrate SST ancestor was deduced to have possessed three sst paralogs, *SST1*, *SST2*, and *SST5*. SST3 and SST6 then arose by duplication of the SST1 and SST2 genes, respectively (16, 56). Our characterization of the somatostatin peptides in the tilapia brain by RNA-seq of mature fish revealed only three SST peptide forms: SST6, SST3, and low levels of SST1. The SSTR subfamily encompasses SSTR1–5, which has several different isoforms in the tilapia genome (tiSSTR2a, tiSSTR3a, tiSSTR5b, and tiSSTR3b). Our syntenic analysis revealed that the studied proteins were affected by the whole genome duplication of the teleosts (57), thus producing several genes encoding different proteins, including the studied receptors (**Table 1**, **Figure 1**). Whole genome duplications described in other receptors and their ligands in teleosts (20, 58–61) increase their functional complexity.

Our results are consistent with previous analyses by Tostivint (62), who found preserved copies of the genes studied in this syntenic analysis. Furthermore, Tostivint and colleagues (62) suggested that the genes *sstr2* and *sstr3* were on the same chromosome in the ancestor of the teleosts. Contrary to the situation observed in the spotted gar, all the genes within paralogons A and B in teleosts were situated on the same chromosome. Therefore, the synteny analysis agrees with the hypothesis of Tostivint and O’Campo-Daza that the ancestral genes were arranged together and gave rise to the different receptors placed on the same chromosome in teleosts (63). The presence of three receptors on the same chromosome in both paralogon A and its duplicate, paralogon B, suggests a unique gene arrangement in teleosts, likely originating from a specific rearrangement involving the genes sstr2, sstr3, and sstr5. This rearrangement led to all three receptors being located on the same chromosome, followed by duplication events.

The gene sstr2-like in the spotted gar likely resulted from a local duplication. Its asymmetric conservation in the teleost paralogon A suggests that the duplication occurred before the teleost whole genome duplication, potentially in their actinopterygian ancestor. However, the gene structure and encoded peptide are highly divergent, as they lack introns and possess only 6 transmembrane domains, unlike the 7 domains that are essential for the proper function of rhodopsin-like GPCRs (42). Thus, the sstr2-like gene may be undergoing a functional alteration that may possibly lead to pseudogenization.

Our phylogenetic analysis showed that the earliest divergence was into a major clade containing SSTR2, SSTR3, and SSTR5. These receptors, as well as SSTR1 and SSTR6, have a monophyletic origin and are basal to the previous clade. The sequences of SSTR4 are basal to the other groups. The receptors SSTR1 and SSTR6, as well as SSTR3 and SSTR5, share a monophyletic origin. A previous investigation explored the hypothesis that two rounds of whole genome duplication, referred to as 2R, influenced the genes encoding the SSTRs (42, 62). According to this framework, the ancestor of vertebrates after the 2R event should have possessed 8 SSTR genes, but only 6 genes are currently identified. This discrepancy may be explained by a loss of 2 genes prior to the radiation of vertebrates, resulting in the present count of 6 somatostatin receptors in extant species. Our phylogenetic analysis aligns with this hypothesis (42, 62). Notably, the absence of a monophyletic origin for a pair of receptors branching with SSTR4 suggests a potential loss of the duplicate *sstr4* gene. Similarly, the clade housing the SSTR2 sequences lacks a coupled clade, indicating that the duplicate of the *sstr2* gene might have been lost following the whole genome duplication events in the vertebrate ancestor.

Within the SSTR1, SSTR2, SSTR3, and SSTR5 clades, two distinct monophyletic groups emerged, separating the terrestrial tetrapods from aquatic species, including the coelacanth. Notably, SSTR4 is absent in nonsarcopterygian species, while SSTR6 remains conserved exclusively in aquatic counterparts, including an ancestral sarcopterygian, the coelacanth. The coelacanth grouping within aquatic species in SSTR1 and SSTR6 suggests that phylogenetic divergence mainly affected terrestrial tetrapods. Previous evolutionary studies found that competing selective pressures for aquatic and terrestrial environments produced unique functions and somatic structures, such as the forelimb locomotor mode (64). Further functional analysis could clarify the association between the sequence divergence in sarcopterygian SSTR receptors and terrestrialization events in the tetrapod lineage.

All receptors in teleosts exhibit a monophyletic origin when present, with the spotted gar serving as the basal species. However, exceptions are noted in the SSTR3 sequences from salmon and carp and in the SSTR5 sequence from salmon. These proteins have undergone significant divergence following the whole genome duplication events specific to salmonids, known as 4R (65, 66) and the allotetraploidy events in carp (67). A comprehensive investigation into the conservation of duplicated genes and their functions in salmonids, as highlighted by (68), suggests that genes retained as duplicates after the 3R are likely to persist after the 4R. Consequently, as in our analysis, highly divergent genes are conserved after the 4R and might position outside the major clades or in positions basal to their groups.

In fish, as in mammals, multiple subtypes of SSTRs are found in many tissues, including the kidney, thyroid, adrenal gland, GI tract, and brain (69). Four distinct subtypes (SSTR1–3 and SSTR5) and several SSTR isoforms have been characterized in different fish species (reviewed in (70). We identified the receptor types that are expressed in defined pituitary cells by RNA-seq of LH and FSH cell populations separated from the tilapia pituitary (21, 71). Three distinct subtypes of SSTRs (SSTR2a, SSTR3a, and SSTR5b) and one isoform (SSTR3b) were found at different levels in these gonadotrophs. SSTR3a is enriched in LH cells, while SSTR3b is significantly enriched in FSH cells. The most highly expressed SSTR variants in the somatotrophs were SSTR5b and SSTR3b. SSTR3a was the most abundant SST receptor in LH cells, pointing to the importance of somatostatin in regulating the LH gonadotropin (21).

SSTRs have already been found in the mammalian hypothalamus and pituitary. SSTR2a is the most prevalent SSTR subtype in GnRH neurons in male and female rats (72) and mice (73). In the rat pituitary, SSTR mRNA was widely distributed across major endocrine cell groups. Mammalian somatotrophs showed relatively high expression levels of SSTR4 (which is absent in fish) and SSTR5b, whereas SSTR2a was predominantly expressed in thyrotrophs and LH cells (74). Knowledge of SSTR distribution in specific pituitary and brain cells of fish is minimal. Our data indicate that SST directly regulates LH and FSH at the pituitary level, thereby expanding its previously recognized role in GH release.

The SSTR signal is transduced by intracellular mediators, such as Ca^2+^, cAMP, cGMP, and nitric oxide (NO) (17). In general, cAMP has appeared as the most prominent signaling pathway for both gene transcription and secretion of SST [reviewed in (75)]. Here, we used both an SSTR agonist and an antagonist to study the signal transduction of these receptors in tilapia. CRE-luc was increased dose-dependently in all SSTRs in the presence of a constant concentration of SSTR agonist (10 nM), whereas it was repressed by different doses of octreotide and a constant dose of SSTR antagonist. Cyclosomatostatin is a nonpeptide SSTR3a-selective antagonist (76). While it elevated cAMP activity in all SSTRs, the highest response was seen in SSTR3a. Conversely, octreotide is an SSTR agonist that binds with high affinity to human SSTR2 and SSTR5 and with moderate affinity to SSTR3a (44).

Our results revealed a similar pattern in tilapia, as SSTR2a had the highest response, followed by SSTR5b. However, SSTR3a was more strongly inhibited by octreotide than by the native tilapia SSTs. Taken together, our results suggest that the binding sites of tilapia SSTRs are similar to those of the human receptors. When SSTR3b was tested solely against a backdrop of FSK, octreotide demonstrated no discernible activation. However, when SSTR3b was activated by cyclosomatostatin in the presence of both FSK and octreotide, efficient activation was observed. This discrepancy might be explained by considering that FSK alone may not have been adequate to elevate cellular cAMP levels sufficiently to detect suppression.

The SSTR subtypes exhibited nearly identical affinity for the endogenous peptide SST14, although SST14 had only 40–55% sequence homology and substantial variation in the extracellular region containing the ligand interaction site (31). This suggests that the binding pocket residues and cavities have substantial similarity. Therefore, we performed binding-site analyses on SSTRs expressed in the tilapia pituitary (tiSSTR2a, tiSSTR3a, tiSSTR5b, and tiSSTR3b). All these SSTRs displayed characteristics of canonical binding sites of peptide-binding class A GPCRs. Studies in huSSTR have suggested that both the extracellular half of the TM domains and the ECLs play essential roles in ligand binding and subtype selectivity (31). Similarly, the orthosteric binding sites in selected tiSSTRs were located on the upper region of the transmembrane cavity and included the ECD and the ECL loops. In huSSTR1, the ECL2 is reported to influence selectivity for the binding of particular peptides (77), whereas ECL3 and its adjoining transmembrane-spanning regions are reported to be critical for the binding of selective agonists (78). The allosteric site reported in human SSTR1 and murine SSTR2 is located in a similar region, albeit deeper within the hydrophobic cleft, and shows typical responses to agonistic and antagonistic compounds, similar to those reported for hSSTR1 (79).

Although the reduction of GH release by SST has been well established in tilapia (39), the possible effect of SST on FSH and LH release was unknown. Therefore, we examined the influence of an IP injection of an SSTR agonist and antagonist on tilapia FSH, LH, and GH plasma levels. In our study, the SSTR antagonist raised LH and FSH levels for 2 h post-injection. LH returned to baseline after 4 h, while FSH remained elevated for 6 h. Octreotide, an SSTR agonist, reduced FSH levels similarly to GH but had no significant effect on LH levels. The high-affinity SSTR agonist octreotide mainly treats acromegaly and pituitary adenomas (44). In female sheep, intracerebroventricular (ICV) infusion of SST abolished pulsatile LH secretion and decreased LH release (80). In rats, the suckling-induced activation of SST-SSTR2a signaling mediated the suppression of pulsatile LH secretion during lactation (81). The lack of an effect of octreotide on LH levels in our study is inconsistent with these previous reports on different species.

SST inhibits the secretion of LH from the pituitary in rats (12), and it also affects LH secretion indirectly by reducing GnRH activity in goldfish, common carp, and grass carp [reviewed in (13)]. Possibly, the concentration of the SSTR agonist used in our experiment (100 µg/kg BW) was insufficient to affect LH levels. A previous study demonstrated a decrease in aggressive behavior in cichlid fish using a higher dose of octreotide (4 mg/kg) (45). In another study, SST administration directly to the target cells via ICV infusion in male rats over a five-day period suppressed the activity of LH-positive pituicytes (12). Our data suggest that SST has similar effects on GH and FSH in tilapia, but has no effect on LH release. Levels of FSH, which regulate gonad growth and development, increase during vitellogenesis in tilapia, whereas LH is responsible for final oocyte maturation and ovulation (40, 82, 83).

The intricate interplay between FSH and GH in fish is required to balance growth and reproduction. The role of GH in the different reproductive stages of fish is not fully understood. GH stimulates the synthesis and secretion of vitellogenin, probably because it enhances estradiol in the liver (84). The vitellogenic phase is characterized by rising levels of both GH and FSH (68). This is consistent with the decreased FSH and GH levels we observed in response to the GH agonist. In both goldfish and tilapia, LH levels increase before spawning and decrease afterward (40, 85). In tilapia, somatic growth increases significantly after spawning (86), and GH levels remain high, while LH levels decrease (39). This suggests that GH may play a role in promoting somatic growth after spawning, and this may be reflected in the increase in LH levels in response to a GH antagonist.

In summary, we have provided a demonstration of a direct regulation of gonadotropin release by somatostatin. We have identified the specific SSTR types expressed on LH and FSH cells and mapped their co-localizations. Indications were also found that SST may directly regulate LH and FSH secretion into the bloodstream. These findings suggest a possible involvement of SST and its receptors in the interplay between growth and reproductive processes and a possible bridging function for SST between the somatic and reproductive axes.

## Data availability statement

The datasets presented in this study can be found in online repositories. The names of the repository/repositories and accession number(s) can be found in the article/**Supplementary Material**.

## Ethics statement

The animal study was approved by Hebrew University administrative panel for laboratory animal care. The study was conducted in accordance with the local legislation and institutional requirements.

## Author contributions

MN: Conceptualization, Data curation, Methodology, Visualization, Writing – original draft. H-CL: Data curation, Writing – review & editing. AI: Conceptualization, Data curation, Methodology, Writing – original draft. SM: Data curation, Methodology, Writing – review & editing. AC: Data Curation, Writing – review & editing. L-SB: Conceptualization, Funding acquisition, Methodology, Supervision, Writing – review & editing.

## Supplementary material

Supplementary Table 1
**(A)** (sstr2): Detailed information on the start and end positions of neighboring genes flanking the sstr2 gene within the spotted gar genome. The table features Ensembl accession numbers for these genes. Feasible, corresponding genes have been matched in teleost species such as Asian bonytongue, European seabass, zebrafish, tilapia, and fugu. The table additionally presents the start and end positions, along with the accession numbers, of these genes in the teleost species. **(B)** (sstr3): Detailed information on the start and end positions of neighboring genes surrounding the sstr3 gene in the spotted gar genome. Ensembl accession numbers for these genes are included. Comparable genes have been identified, wherever possible, in teleost species like Asian bonytongue, European seabass, zebrafish, tilapia, and fugu. The table also provides the start and end positions, as well as the accession numbers, of these genes in the teleost species. **(C)** (sstr5): Detailed information on the start and end positions of neighboring genes flanking the sstr5 gene within the spotted gar genome. The table contains Ensembl accession numbers for these genes. Comparable genes have been identified, when feasible, in teleost species including Asian bonytongue, European seabass, zebrafish, tilapia, and fugu. Additionally, the table presents the start and end positions, along with the accession numbers, of these genes in the teleost species.

SUPPLEMENTARY FILE 1 Sequences of the Somatostatin receptors used in **Figure 2** constructing the phylogenetic tree.

Supplementary Table 2 Uniprot IDs of the SSTRs used in for constructing the phylogenetic tree.
