# Supplementary material for: Characterization of the somatostatin system in tilapia: implications for growth and reproduction
Source: Front Endocrinol (Lausanne). 2024 Jun 21;15:1302672. doi: 10.3389/fendo.2024.1302672 (PMC11224465; doi:10.3389/fendo.2024.1302672)
Supplement: Supplementary file 5 [file DataSheet_5.docx]

**Characterization of the Somatostatin System in Tilapia: Implications for Growth and Reproduction**

Mizrahi Naama, Hollander-Cohen Lian, Atre Ishwar, Shulman Miriam, Aurora Campo and Levavi-Sivan Berta

**Supplementary File 1 -** Sequences of the Somatostatin receptors used in Figure 2 (the phylogenetic tree)

>HUMAN SSTR1 Uniprot ID:P30872

MFPNGTASSPSSSPSPSPGSCGEGGGSRGPGAGAADGMEEPGRNASQNGTLSEGQGSAIL

ISFIYSVVCLVGLCGNSMVIYVILRYAKMKTATNIYILNLAIADELLMLSVPFLVTSTLL

RHWPFGALLCRLVLSVDAVNMFTSIYCLTVLSVDRYVAVVHPIKAARYRRPTVAKVVNLG

VWVLSLLVILPIVVFSRTAANSDGTVACNMLMPEPAQRWLVGFVLYTFLMGFLLPVGAIC

LCYVLIIAKMRMVALKAGWQQRKRSERKITLMVMMVVMVFVICWMPFYVVQLVNVFAEQD

DATVSQLSVILGYANSCANPILYGFLSDNFKRSFQRILCLSWMDNAAEEPVDYYATALKS

RAYSVEDFQPENLESGGVFRNGTCTSRITTL

>HUMAN SSTR2 Uniprot ID:P30874

MDMADEPLNGSHTWLSIPFDLNGSVVSTNTSNQTEPYYDLTSNAVLTFIYFVVCIIGLCG

NTLVIYVILRYAKMKTITNIYILNLAIADELFMLGLPFLAMQVALVHWPFGKAICRVVMT

VDGINQFTSIFCLTVMSIDRYLAVVHPIKSAKWRRPRTAKMITMAVWGVSLLVILPIMIY

AGLRSNQWGRSSCTINWPGESGAWYTGFIIYTFILGFLVPLTIICLCYLFIIIKVKSSGI

RVGSSKRKKSEKKVTRMVSIVVAVFIFCWLPFYIFNVSSVSMAISPTPALKGMFDFVVVL

TYANSCANPILYAFLSDNFKKSFQNVLCLVKVSGTDDGERSDSKQDKSRLNETTETQRTL

LNGDLQTSI

>HUMAN SSTR3 Uniprot ID:P32745

MDMLHPSSVSTTSEPENASSAWPPDATLGNVSAGPSPAGLAVSGVLIPLVYLVVCVVGLL

GNSLVIYVVLRHTASPSVTNVYILNLALADELFMLGLPFLAAQNALSYWPFGSLMCRLVM

AVDGINQFTSIFCLTVMSVDRYLAVVHPTRSARWRTAPVARTVSAAVWVASAVVVLPVVV

FSGVPRGMSTCHMQWPEPAAAWRAGFIIYTAALGFFGPLLVICLCYLLIVVKVRSAGRRV

WAPSCQRRRRSERRVTRMVVAVVALFVLCWMPFYVLNIVNVVCPLPEEPAFFGLYFLVVA

LPYANSCANPILYGFLSYRFKQGFRRVLLRPSRRVRSQEPTVGPPEKTEEEDEEEEDGEE

SREGGKGKEMNGRVSQITQPGTSGQERPPSRVASKEQQLLPQEASTGEKSSTMRISYL

>HUMAN SSTR4 Uniprot ID:P31391

MSAPSTLPPGGEEGLGTAWPSAANASSAPAEAEEAVAGPGDARAAGMVAIQCIYALVCLV

GLVGNALVIFVILRYAKMKTATNIYLLNLAVADELFMLSVPFVASSAALRHWPFGSVLCR

AVLSVDGLNMFTSVFCLTVLSVDRYVAVVHPLRAATYRRPSVAKLINLGVWLASLLVTLP

IAIFADTRPARGGQAVACNLQWPHPAWSAVFVVYTFLLGFLLPVLAIGLCYLLIVGKMRA

VALRAGWQQRRRSEKKITRLVLMVVVVFVLCWMPFYVVQLLNLFVTSLDATVNHVSLILS

YANSCANPILYGFLSDNFRRFFQRVLCLRCCLLEGAGGAEEEPLDYYATALKSKGGAGCM

CPPLPCQQEALQPEPGRKRIPLTRTTTF

>HUMAN SSTR5 Uniprot ID:P35346

MEPLFPASTPSWNASSPGAASGGGDNRTLVGPAPSAGARAVLVPVLYLLVCAAGLGGNTL

VIYVVLRFAKMKTVTNIYILNLAVADVLYMLGLPFLATQNAASFWPFGPVLCRLVMTLDG

VNQFTSVFCLTVMSVDRYLAVVHPLSSARWRRPRVAKLASAAAWVLSLCMSLPLLVFADV

QEGGTCNASWPEPVGLWGAVFIIYTAVLGFFAPLLVICLCYLLIVVKVRAAGVRVGCVRR

RSERKVTRMVLVVVLVFAGCWLPFFTVNIVNLAVALPQEPASAGLYFFVVILSYANSCAN

PVLYGFLSDNFRQSFQKVLCLRKGSGAKDADATEPRPDRIRQQQEATPPAHRAAANGLMQ

TSKL

>MOUSE SSTR1 Uniprot ID:P30873

MFPNGTASSPSSSPSPSPGSCGEGACSRGPGSGAADGMEEPGRNASQNGTLSEGQGSAIL

ISFIYSVVCLVGLCGNSMVIYVILRYAKMKTATNIYILNLAIADELLMLSVPFLVTSTLL

RHWPFGALLCRLVLSVDAVNMFTSIYCLTVLSVDRYVAVVHPIKAARYRRPTVAKVVNLG

VWVLSLLVILPIVVFSRTAANSDGTVACNMLMPEPAQRWLVGFVLYTFLMGFLLPVGAIC

LCYVLIIAKMRMVALKAGWQQRKRSERKITLMVMMVVMVFVICWMPFYVVQLVNVFAEQD

DATVSQLSVILGYANSCANPILYGFLSDNFKRSFQRILCLSWMDNAAEEPVDYYATALKS

RAYSVEDFQPENLESGGVFRNGTCASRISTL

>MOUSE SSTR2 Uniprot ID:P30875

MEMSSEQLNGSQVWVSSPFDLNGSLGPSNGSNQTEPYYDMTSNAVLTFIYFVVCVVGLCG

NTLVIYVILRYAKMKTITNIYILNLAIADELFMLGLPFLAMQVALVHWPFGKAICRVVMT

VDGINQFTSIFCLTVMSIDRYLAVVHPIKSAKWRRPRTAKMINVAVWCVSLLVILPIMIY

AGLRSNQWGRSSCTINWPGESGAWYTGFIIYAFILGFLVPLTIICLCYLFIIIKVKSSGI

RVGSSKRKKSEKKVTRMVSIVVAVFIFCWLPFYIFNVSSVSVAISPTPALKGMFDFVVIL

TYANSCANPILYAFLSDNFKKSFQNVLCLVKVSGTEDGERSDSKQDKSRLNETTETQRTL

LNGDLQTSI

>MOUSE SSTR3 Uniprot ID:P30935

MATVTYPSSEPTTLDPGNASSTWPLDTTLGNTSAGASLTGLAVSGILISLVYLVVCVVGL

LGNSLVIYVVLRHTSSPSVTSVYILNLALADELFMLGLPFLAAQNALSYWPFGSLMCRLV

MAVDGINQFTSIFCLTVMSVDRYLAVVHPTRSARWRTAPVARTVSAAVWVASAVVVLPVV

VFSGVPRGMSTCHMQWPEPAAAWRTAFIIYTAALGFFGPLLVICLCYLLIVVKVRSTTRR

VRAPSCQWVQAPACQRRRRSERRVTRMVVAVVALFVLCWMPFYLLNIVNVVCPLPEEPAF

FGLYFLVVALPYANSCANPILYGFLSYRFKQGFRRILLRPSRRIRSQEPGSGPPEKTEEE

EDEEEEERREEEERRMQRGQEMNGRLSQIAQAGTSGQQPRPCTGTAKEQQLLPQEATAGD

KASTLSHL

>Mouse SSTR4 Uniprot ID:P49660

MNAPATLPPGVEDTTWTPGINASWAPDEEEEDAMGSDGTGTAGMVTIQCIYALVCLVGLV

GNALVIFVILRYAKMKTATNIYLLNLAVADELFMLSVPFARSAAALRHWPFGAVLCRAVL

SVDGLNMFTSVFCLTVLSVDRYVAVVHPLATATYRRPSVAKLINLGVWLASLLVTLPIAV

FADTRPARGGEAVACNLHWPHPAWSAVFVIYTFLLGFLPPVLAIGLCYLLIVGKMRAVAL

AGGWQQRRRSEKKITRLVLMVVTVFVLCWMPFYVVQLLNLFVTSLDATVNHVSLILSYAN

SCANPILYGFLSDNFRRSFQRVLCLRCCLLETTGGAEEEPLDYYATALKSRGGAGCICPP

LPCQQEPVQAEPGCKQVPFTKTTTF

>MOUSE SSTR5 Uniprot ID:O08858 (NP_001177937.1)

MEPLSLTSTPSWNASAASSSSHNWSLVDPVSPMGARAVLVPVLYLLVCTVGLGGNTLVIY

VVLRYAKMKTVTNVYILNLAVADVLFMLGLPFLATQNAVSYWPFGSFLCRLVMTLDGINQ

FTSIFCLMVMSVDRYLAVVHPLRSARWRRPRVAKLASAAVWVFSLLMSLPLLVFADVQEG

WGTCNLSWPEPVGLWGAAFITYTSVLGFFGPLLVICLCYLLIVVKVKAAGMRVGSSRRRR

SERKVTRMVVVVVLVFVGCWLPFFIVNIVNLAFTLPEEPTSAGLYFFVVVLSYANSCANP

LLYGFLSDNFRQSFRKALCLRRGYGVEDADAIEPRPDKSGRPQTTLPTRSCEANGLMQTS

RL

>West Clawed frog SSTR1 Uniprot ID:A0A803J9Q5

MLPNNTSTNVMGSLDNTPEFIWGNNTLNGTSADSPGIAIFISFVYSLVCIVGLCGNSVVI

YVILRYAKMKTATNIYILNLAIADELLMLSVPFLVTSTLLRHWPFGSLLCRLVLSLDAMN

MFTSVYCLAVLSLDRYVAVVHPISAARYRRPSVAKMVNLGVWLFSILIILPIVVFSSTAP

NSDGTVACNVLMPEPSQRWVVVFVLYTFLMGFLLPMAAICLCYILIITKMRVVALKAGWQ

QRRRSERKLTLMVTVVVTVFVVCWMPFYVVQLVGVFARKGDTTVSQLSVALGYANSCANP

FLYGFLSDNFKRSFQKVLCLSWMENANEEPVDYYATALKSRAYSAEELQNGMLTTGGIYS

NGTCTSRTH

>West Clawed frog SSTR2 Uniprot ID:A0A803JIC2

MEQDYSELSNTTELWFTSLSQLDIFGRVTPTNASISNSTTYYDMTSNAILTFIYFVVCIV

GLCGNTLVIYVILRYAKMKTITNIYILNLAIADELFMLGLPFLAMQVALVHWPFGKAICR

IVMTVDGINQFTSIFCLTVMSIDRYLAVVHPIKSAKWRRPRTAKMVNAAVWTVSLLVIMP

IMTYAGVQSYHGRGSCTIIWPGNSSAWYTGFIIYAFILGFLVPLSIICLCYLFIIIKVKS

SGIRVGSSKRKRSEKKVTRMVSIVVAVFIFCWLPFYIFNVSSVSLLIVPTPGLKAMWDFV

VVLSYANSCANPILYAFLSDNFKKSFQNVLCLSKVSGMDEVDRSDSKQDKSRLNETTETQ

RTLLNGDLQTSI

>West Clawed frog SSTR3 Uniprot ID:A0A803K7R3

LIVCFTPIMTPTFPTTLLESSYIEFNATLNTNISFNQNGTSPGLLIPLVYLVVCAVGLWG

NTLVIYLAWRSPAGQNSVTALYILNLALADDLFMLGLPFLAAQNALSYWPFGSPACRLVM

TLDAVNQFTSIFCLTVLSFDRYLAVVRPIQSAKWRKPKVAKCVNVTVWILSFLVVLPVVF

FSGVPGDTGTCHIAWPEPAQMWRTGFILYTAALGFFCPLLVICICHLLIVAQVRSSGKRV

RVAPNRRQGPERKVTKMVALTVTAFVLCWFPFYALNIINLLWPLPESPKLYGLYSFVVAL

SYANSCLNPIIYALLARPFQRGLRRVLCRTSVRVADGTLKRGDDEVQEELSRVNGISQEG

RSVRTDGGEGNSI

>West Clawed frog SSTR4 Uniprot ID:A0A803KEP4

MTTSPNLLVPVEAKILLSTWNESRDMVGTQYPISLIPSNKNTNSTSAPLENEKNVSMIVI

QFIYAIVCLIGLIGNSMVIFVILRYAKMKTATNIYILNLAIADELFMLSVPFLAASAALQ

HWPFGSGMCRTVLSVDGINMFTSVFCLTVLSVDRYVAVVHPLRAARYRRPTVAKMINICV

WIVSLLVISPILIFADTMPSKNGVVVCNLMWPHQTWSAVFVIYTFLLGFFLPVVAICLCY

ILIIIKMRAVALKAGWQQRKKSEKKITRMVLMVVTVFVICWMPFYIVQLLNLFLPHMDAT

INHISLILSYANSCANPILYGFFSDNFKRSFQRIVCFRWLENGTDEPVDYYATALKSRVC

NNNPLDFQQEPLQSDPCYKHGTITRTTTL

>West Clawed frog SSTR5 Uniprot ID:A0A803JBS3

MDPLPLLMASNLEAEMKDINFTLFKNASDNKTVVDPSVSGMSSFIIPLIYLLVCVIGLSG

NTLVIYVVLRYAKMKTVTNIYILNLAVADVLFMLGLPFLATQNAISYWPFGTFLCRLVMT

VDGVNQFTSIFCLTVMSIDRYLAVVHPIKSTKWRRPRVAKLISATVWTLSFLVTLPVIIF

SEVQPDYHTCNISWPDPVSVWAAAFIIYTSVLGFFGPLSVICLCYLLIVIKVKSSGLRVG

STRRRRSERKVTRMVVIIVAVFVFCWLPFYILNIVNLSFFVPEEPAFAGVYFFVVVLSYA

NSCANPILYGFLSDNFKQSFQKVLCLRKSNGIKDADLTENRQEKSSRLQETMLPSRNSEF

NGHMQTSKV

>Gold fish SSTR1 Uniprot ID:A0A6P6LN89

MLPNDTFKNLEDGLYLINFSSNETHNGDSHGSSAIFISFIYSVVCLVGLCGNSMVIYVIF

RYAKMKTATNIYILNLAIADELLMLSVPFLVTSSLLHHWPFGSLLCRLVLSVDAINMFTS

IYCLTVLSIDRYISVVHPIKAARYRRPTIAKMVNLGVWMFSILVILPIIIFSTTAPNSDG

SVACNMQMPEPERQWMAVFVIYAFLMGFLFPVIAICMCYILIIVKMRVVALKAGWQQRKK

SERKITLMVMMVVTVFVICWMPFHIMQLVSVFVQQHNATLSQLAVILGYANSCANPILYG

FLSDNFRRSFQRILCLRWWDNATEEPIDYYATALKSRGYSVDDFQPENLESGSTYRNGTC

TSRTTTL

>Gold fish SSTR3a Uniprot ID:Q8JID5

MELTSVDASAVLDLWGNGSVPGSLLNESILNGTCFLNALNCTNGTDAGNRAGTSMAGILI

PLIYIIVCVVGLGGNSLVIHIVLHYSKTESVTNIYILNLAIADELFMLGLPFLAVQNAMH

SWPFGSFTCRLVMTVDGINQFTSIFCLTVMSIDRYLAVVHPIRSSKWRRPQVAKAENGTI

WAVSFLVVLPVVIFANVQREGGICNIIWPEPANIWGAAFIIYTSTVGFFFPLLVICMCYL

LIVIKIRSSGKKVHATSTKRRKSERKVTRMVVIVVAVFVFCWMPFYALNIINLVESLRDE

PQGLHLFVVVLSYANSCANPIVYCFLSDNFKRGFRKALCRSSRRVENHESTEQQNQEERR

RVLMPRESLKRAVRNEEDEEEEEYREEVTEMTEICRITQNGNGSRQAESSRALFLERPSG

AGVSETSSPDRRGTAGDVKGPGFGTAATLLNGAKNGNVKTLPEEPVEKNSSLEISYL

>Gold fish SSTR3b Uniprot ID:Q7T2S8

MELHQCLSPPSTPEAPVWTNASSSMIPNQTFPPPYLLQTENLTDSRTLDYAPGVAGILIP

LIYIVVCIMGLIGNTLVIHIVLRYSQAESVTNIYILNLAIADELFMLGLPFLAVQNGLLS

WPFGSLMCRLVMTVDAINQFTSIFCLTVMSIDRYLAVVHPLQSSRWRQPRVAKMVNATVW

GISFVVVLPVVVFAGVLQDDGNCSIVWPEPAEVWKATFIVYTATVGFFGPLAVICLCYLL

IVVKVRSSGRRVRATSIRRRKSECKITRMVVIVVAVFVFCWLPFYVLNIVNLLVLLPGDF

RGLYYFVVVLSYANSCANPILYGFLSDNFKRGFRKALCRSSRRVENQDQQQIMGMVVLPL

EEIQRELDPNEHLKVTEVEEHISDHEMRKLQNGCREDSRIADAAGGMDGRSERSISLTSD

PPAVRNGEQQTHKHLTENSNDRRSVLEISFVM

>Goldfish SSTR5 -like-a Uniprot ID:Q8QGQ4

MATQGPMCNTSIPDYTNQSNETTNFSLQQNGDSMSEEDSTKVLAVIYVVVFIVGLTGNSL

AIFVVLRYTKMKTVTNMYILNLAVADELYILGIPFLTTHNVLGYWPFGNFLCRIVMWADS

ISQFTSTFCLTVMSIDRYLAVVHPIRSTRWRRPSVARVINSMVWALSCLLTLPVIIYCDV

QPELNTCNLSWPEPRDMWSTAFILYTAILGFFCPLLIICLCYLLIVIKVKSAGVRAGLSK

RRSSEKKVTRMVVIIVVVFALCWLPFFFLNIFNLISTLPENGLVSGIYFLTVILTYVNSC

ANPLLYGFLSDNFKRSFKQVLCIHRVNGVSEVHPVHARLSRSQQNEPFFSPRSSDFNENV

QSGHSIGLETEASAKTEIHQLGPEVSSQSI

>Goldfish SSTR5 -like-b Uniprot ID:Q7T2S9

MESINQSGLVGTIPGDYNSTFGMGFSGSPVGNYTGNVSDQSMPFQGSSTMVTAVISFTVF

IVGLTGNTLAIYVVLRYAKMKTVTNIYILNLAVADELYIMGLPFLTTQNVLSYWPFGSFL

CRVVMTADSLNQFTSIFCLTVMSIDRYLAVVHPIRSTKWRRPRVAKAVSAAVWAFSFIVV

LPVVIFSDVQDTFNSCNMSWPEPRDIWSTAFILYTATLGFFGPLLVICMCYLLIVVKVKS

SGARAGFTKRRRSERKVTRMVVVIVVVFVLCWLPFYIINIVNLVFILPENSVMAGVYFFA

VILSYANSCANPLLYGFLSDNFKQSFRKVLCVRKTNGVEDGDPSVPRTEKTTTQETFLAP

RNNDFNGHAQGSQVQSL

>Mandarin fish SSTR2 (Siniperca chuatsi) Uniprot ID:I6R7Y7

MALDQWPFLPTPPNISIPEPLLYDSYIQGNESDLDLNISETREPHQDKTSSVVITFIYFM

VCAVGLCGNALVIYVILRYAKMKTVTNIYILNLAVADVLCMMSLPFIALQLTLVHWPFGE

VLCRVIMTVGSLNQFTSIFCLMVMSIDRYFAVVHPIKSTKWRKPRVAKLINLTVWVVSLL

VILPTMIFSGLDKVPVCGIVWPEPQDVYYTAFIFYTFFIGFFLPLAVICLCYLLIIVRVK

SSGIRVGSTKRKRSERKVTRMVSIVVAVFVLCWLPFYIFNVTSVTGSIKPTSAVKSTFDF

VVVLGYANSCANPILYAFLSDNFKKSFQNVLCLKKVAGLDEIERSDSRADRSRMANDAAI

INANLETHNAALLNGELQTSI

>Mandarinfish SSTR3 (Siniperca chuatsi) Uniprot ID:I6S3S9

MGVVSLPGLEMTDEMWENGSSASPSPGPPLFLLMFNDSEMNETLFNSTNSTTADVSSGPG

VAGVLISLIYIVVCIIGLGGNTLVIHIVLHYSKIESVTNIYILNLAIADELFMLGLPFLA

VQNTLQSWPFGSFMCRLVMTVDSINQFTSIFCLTVMSIDRYLAVVHPIRSSKWRRPQVAK

VVNCTVWALSFLVVLPVVIFANIQKAGGTCNIAWPEPAKIWRAAFIIYTSTVGFFCPLLI

ICLCYLLIVFRIRSSGKKVHATSTKRRKSERKVTRMVVIVVAVFVFCWLPFYALNIINLL

VSLPSEYQGLYYFVVVLGYANSCANPIVYGFLSENFKRGFRKALCRSTRKVENHEPMERQ

QQQEEGRMALMPRESLRRAIGDEEDDDEEDVSEMTEIYRIAQNGNSSFQPQGSQPLLSEK

RATPRATEPSSPDERDKAGDTKGKDQVNGSTLTVPLLLNGTKNGSIKTLPEEGLEQSTSL

EISYL

>Orange spotted grouper SSTR1 Uniprot ID:D8L207

MQLNTSSSSSSSSSSSPLPATLSSVEDGGGFSLLNGAGNGSVSGGGAPSAGSAALISSIY

SVVCVVGLSGNSMVIYVIFRYAKMKTATNLYILNLAVADELLMLSVPFVVTDALLRRWPF

GAALCHLVLSVDAINMFTSIYCLTVLSVDRYIAVVHPLRASRYRRPTVAKLVNACVWMFS

LLVILPIILFSSTAPNSDGSVACNMQMPEPEHRWMAVFAVYAFLMGFLFPVLAICLCYVL

ILSQLRAVALRAGWQQRRKSERKITVMVTVVVSVFVVCWMPFHVVQLVGVFLQRHDPILS

QLAVVLGYANSCANPVLYGFLSDNFRRSFQRILCMRWMEAPEEPLDYLDYYSTALQSRRV

SLDQNHHEDQEEDRTCPPSNSSRM

>Orange spotted grouper SSTR2 Uniprot ID:D2CSU1

MALDQWPFLPTPPNISIPEPLLYDSYIQGNESDLDLNITETRELHQDKTSSMAITFIYFI

VCTVGLCGNALVIYVILRYAKMKTVTNIYILNLAVADVLCMMSLPFIALQLALVHWPFGE

VLCRVIMTVDSLNQFTSIFCLMVMSIDRYLAVVHPIKSTKWRKPRVAKLINLTVWGVSLL

VILPTMIFSGLDKVPVCGIVWPEPQDVYYTAFIIYTFFIGFFLPLAVICLCYLLIIVKVK

SSGMRVGSTKRKRSERKVTRMVSIVVAVFVLCWLPFYIFNVTSVTSSINPTSAVKSTFDF

VVVLGYANSCANPILYAFLSDNFKKSFQNVLCLKKVAGLDEIERSDSRADRSRMVNDAMI

ISANLETHNAALLNGELQTSI

>Orange spotted grouper SSTR3 Uniprot ID:D2CSU2

MGVVSLPELEMMDETWENSSFASPSPGFPLLLLIFNNSDLNETFLNDTNPKNSTEQGVPS

GLSVAGVLIPLLYIIVCIIGLGGNTLVIHIVLHYSKIESVTNIYILNLAITDELFMLSLP

FLAVQNTLHSWPFGSFMCRLVMTVDSINQFTSIFCLTVMSIDRYFAVVHPIRSSKWRRPQ

VAKMVNGTVWAVSFLVVLPVVIFSKIQRSGGTCNIAWPQPANIWSAAFIIYTSTVGFFCP

LLIICLCYLLIVFKIRSSGKKVHATSTKRRKSERKVTRMVVIVVAVFVFCWLPFYALNII

NLLVLLPPEYQSLYYFFVVLGYANSCANPIVYGFLSDNFKRGFRKALCRSTRKVENHEPM

ERQQQQEEGRTALMPRESLRRAIRDEEDDDEEDLSEMTEIYRIAQNGNSSFQPQSSQPLL

SEKGATTAATELSSPDRKDKSGEMKGKDPINGSTLTVPLLLNGTKNGSIKTLPEENLDQS

TSLEISYL

>Orange spotted grouper SSTR5 Uniprot ID:D2CSU3

MDGYYNWTLTSENGSMSSSQPYPRDHTYNNTSETAAPMPFSTVTAVVYTIVFIVGLLGNT

LVIYVVVRYTKMKTVTNMYILNLALADELYILGIPFLGTNSALSYWPYGDFFCKVCMTAD

SMSQFSSTFCLTVMSIDRYLAVVHPIRSAKWRKPQVAKVFNFMVWVVSFLIVLPVTIYSH

VQEEFNTCNITWPEPRELWSIVFILYTSILGFFGPLIVICLCYLLIVIKVRSAGARAGLT

KRRKSERKVTRMVVIIVLVFVLCWMPFYTTNIVNLFHTIPENNTTAAVYFFLVILTYVNS

CANPVLYGFLSDNFKQSFQKVLCFHKPNGAGMAGQVGGRQTSPQENHNPGFSPRNPAQNG

KPQSIQVLCKKYSCNPCDWLIAMLVGCDVGC

>Carp SSTR1b Uniprot ID:A0A9J7XE53

MAELAGYNGSENFVFSTGLPFNSTGDYEYYEPEPDASKIIIPSIYALVCCVGVTGNAMVIYVILKYAKMK

TATNIYILNLAIADELFMLSVPFLATSAAVHHWPFGSLMCRLVLSVDGINMFTSIFCLTVLSVDRYIAVV

HPIKAARYRRPTVAKVVNVCVWGLSLLVILPIIIFADTVPAQDGGVDCNFLWPESSWSEAFVVYTFLLGF

LLPVAAICLCYCLIVVRMRAVGLKAGWLQRRRSEKKITRMVLLVVAVFVLCWMPFYIVQLISVFRKPPDP

MVTQLFVILSYANSGANPILYGFVSDNFRRSFQRIICFRWLENGLDAEQVDYCAVALRRQTTCGPPNFPK

ECLASDMVFRNGTCTSRTTTL

>Carp SSTR2 Uniprot ID:A0A8C0YL69

MMDSWTFSFSPSNLPSNLSGLPMYDSILLGNISEEGLRNQTDQSLTVIITFMYFVVCAVGLCGNALVIYV

ILRYAKMKTVTNVYILNLALADVLFMLSLPFIALQLALVHWPFGAALCRVVITVDSLNQFTSIFCLMVMS

IDRYLAVVHPIKSTKWRKPQVAKTINLVVWVVSLVVNLPIVIYSGLITKPDGCFCTIVWPEPQETYYTVF

MFYTFFLGFFLPLMVICLCYLFIIIKVKSSGIRVSSSKRRQSERRVTRMVSIVVAVFVFCWLPFYIFNVT

SVTGTISTTPFLRSMFAFVVVLGYANSCANPILYAFLSENFKKSFQKVLFLKKAVALDEVEHTDSKQDKA

RIMNEPTETRDTLLNGDLQTSI

>Carp SSTR3 Uniprot ID:A0A8C1LV85

MELTSVDASAVLGLWGNGSIGFLLNESMLNDTCFLNTSNCTNGRDAGSRAGTSMAGILIP

LIYIIVCIVGLGGNSLVIHIVLHYSKTESVTNIYILNLAIADELFMLGLPFLAVQNAMHS

WPFGSFTCRLVMTVDGINQFTSIFCLTVMSIDRYLAVVHPIRSSKWRRPQVAKAVNGTIW

AVSFLVVLPVVIFANVQREGGICNIIWPEPANIWGAAFIIYTSTVGFFFPLLVICMCYLL

IVIKIRSSGKKVHATSTKRRKSERKVTRMVVIVVAVFVFCWMPFYALNIINLVESLRDEP

QGLHLFVVVLSYANSCANPIVYGFLSDNFKRGFRKALCRSSRRVENHEPTEQQNQEEGRR

VLMPRESLRRAVRDEEDEEEEEYREEVTEMTEICRITQSANVSGQPESTRSLFLERPSGT

GVSETCSPDRRGVDVLGKGPGFGPSATLLNGAKNGNVKTLPEEPVEKNTSLEISYL

>Carp_SSTR3_X1 (XP_042574638.1) Uniprot ID:A0A9Q9W0L5

MGFKLCLEHVPVIASVSNRDWLISTQSIMPAIVNLLFNTVSTNTTISFSLVLPLVSILSLLVGVGGHLLM

WLVLMRNPRRRSKPSSVLLLNLSFADLCALLTLPCVLLSASSQNWQLGGGVCVLLSFMTSLTAGVDIFSL

AALSVLRYRIVAPSTRPPATPTQVAGTVAVIWLVSIAMALPKVTYIQFDSGCTWSVGRGHWLGFLVPAFL

VYYVAPLLCIALHCGLIITHLYRCRGTLAADHRNKKATALLIGSTLVFAISWLPYYVLEFVNVLSPSLNS

VSSPESPASSSTAPSPASSTEVSLLWEVASLSAILLICLAPCWNPPLYFLLSKPALRQLRGLLPIMHQHW

RAATFLQHIVPKHAPTQPHSQPGSQHVPQNILEVTHPQ

>Carp SSTR5_A0A8C2FGM0

MELTSVDASAVLDLWGNGSIPGFLLNESILNDSCFLNALNCTNGTDAGNRAGTSMAGILIPLIYIIVCIV

GLGGNSLVIHIVLHYSKTESVTNIYILNLAIADELFMLGLPFLAVQNAMHSWPFGSFTCRLVMTVDGINQ

FTSIFCLTVMSIDRYLAVVHPIRSSKWRRPQVAKAVNGTIWAVSFLVVLPVVIFANVQREGGICNIIWPE

PANIWGAAFIIYTSTVGFFFPLLVICMCYLLIVIKIRSSGKKVHATSTKRRKSERKVTRMVVIVVAVFVF

CWMPFYALNIINLVESLRDEPQGLHLFVVVLSYANSCANPIVYCFLSDNFKRGFRKALCRSSRRVENHEP

TEQQNQEERRRVLMPRESLRRAVREEEDEEEEEYREEVTEMTEICRITQNGNGSRQPESTRALFLERPSG

TGVSETSSPDKRGTGGDAKGPGFGTAATLLNGAKNGNVKTLPEEPVEKNNSLEISYL

>Atlantic Salmon SSTR1 Uniprot ID:A0A1S3RDW0

MLPNSSFRNLTLEDGFFLMNNSSGNETYSESQGSAILISFIYSVVCLVGLCGNSMVIYVI

FRYAKMKTATNIYILNLAIADELLMLSVPFLVTSSLLHHWPFGSLLCRLVLSVDAINMFT

SIYCLTVLSIDRYIAVVHPIKASRYRRPTIAKIVNFGVWMFSILVILPIIIFSTTVPNLD

GSVACNIQMPKPVNQWMAVFVVYAFLMGFLFPVIAICMCYILIIAKMRVVALKAGWQQRK

KSERKITLMVMMVVTVFVICWMPFHIVQLVNVFVEHHNATLMQLAVILGYANSCANPILY

GFLSDNFKRSFQRILCLRWMDNATEEPIDYYATALKSRGYSVDEFQPDNIECDSTYRNGT

CTSRTTTL

>Atlantic Salmon SSTR2 Uniprot ID:A0A1S3Q3V3

MDLWPLLPSSPNLSLAEPLYYDSYFPGNESDLGSRNDTPDETQQVFDKTSSVVITFIYFM

VCAVGLTGNTLVIYVILRYAKMKTVTNIYILNLAVADVLCMLSLPFIAMQLALVHWPFGA

VLCRLVMTVDCLNQFTSIFCLTVMSIDRYLAVVHPIKSTKWRKPRVAKIINLTVWGVSLL

VNLPIMIFSGLMPNKNQAWVCTIVWPEPQEAYQTAFMFYTFFLGFFLPLTVICLCYLLII

VKVKSSGVRVGSTKRKCSERKVTRMVSIVVAMFVLCWLPFYVFNVTSVTGTIDTTPVLKS

TFEFVVVLGYANSCANPILYAFLSDNFKKSFQNVLCLKKVAGLDEVERSDSRMDRTRMVK

DITAATHNAALLNGELQTSI

>Atlantic Salmon SSTR3 Uniprot ID:A0A1S3R6B7 (XP_014047284.1)

MPAIVNLLFNTVSTNTTISFSLVLPLVSVLSLLVGVGGHLLMWLVLMRNPRRRSKPSSVL

LLNLSLADMGALLTLPCVLLSASSQDWQLGGGICVLLGFMTSLTAGVDIFSLAALSVLRY

RIVAPPARPPASPTQVAGIVAVIWLVSVTMALPKVTYIQFDSGCTWSVGRGQWLGFLVPA

FLVYYVAPLLCIALNCGLIITHLHRCRGTLAADRRNKKATALLIGSTLVFAISWLPYYAL

EFVNVMSPYVSFVASPISQQPSSSLNSSASLSPPTEGDTEVSLLWEVASLSAILLVCLAP

CWNPPLYFLLSRPAVRQLRALLLSLRKCLGSLRPPITPAPPPRFPHLRPLPTL

>Atlantic Salmon SSTR5-like Uniprot ID:A0A1S3NF59

MDSSFTSETFYNAGSIADPTTSYVDEDMYLQEDLDVFSVTMAVLYLAVCIIGLGGNTLVIVAILKLDKMA

SATTVYIFNLALADGLFMVGLPFIAIQNFQNHWAFGDLACKLVMVLDGINQFTSVFCLTVMSIDRYMALV

DPLRFARWRTPKRAKIVSGFLWLFSLLPVLPMTIHFSARDGLCNLDPQVASDSWWLAFITYTFVLGFALP

FLVMIVSYTALVVTLRTHRHQASSPGQESPRLETQVTKMVVAVVLAFAVCWLPFYAFNFCSLYHTDLVLT

FARCFEFVVLLSYSWSCANPILYACLSETFGRHFLTLLCPTKRFPSVQCNPDTERYDLNDTSGMGNSAVV

>Zebrafish SSTR1a_ A0A0R4IV97

MLPNDTFKNLEDGLYLLNSSNETHNGDAHGSSAIFISFIYSVVCLVGLCGNSMVIYVIFR

YAKMKTATNIYILNLAIADELLMLSVPFLVTSSLLHHWPFGSLLCRLVLSVDAINMFTSI

YCLTVLSIDRYISVVHPIKAARYRRPTIAKMVNLAVWMFSILVILPIIIFSTTAPNSDGS

VACNMQMPEPERQWMAVFVIYAFLMGFLFPVIAICMCYILIIVKMRVVALKAGWQQRKKS

ERKITLMVMMVVTVFVICWMPFHIVQLVSVFVQQHNATLSQLAVILGYANSCANPILYGF

LSDNFRRSFQRILCLRWWENATEEPIDYYATALKSRGYSVDDFQPDNLESDSTYRNGTCT

SRTTTL

>Zebrafish SSTR1b_ A0A1D5NSQ4

MAELAGYNGNESFMLATDLPFNSTGDYEYYESEPDASKIIIPSIYALVCCIGVTGNAMVI

YVILKYAKMKTATNIYILNLAIADELFMLSVPFLATSAAVHHWPFGSLMCRLVLSVDGIN

MFTSIFCLTVLSVDRYIAVVHPIKAARYRRPTVAKVVNVCVWGLSLLVILPIIIFADTVP

AQDGGVDCNFMWPESSWSEAFVVYTFLLGFLLPVGAICLCYCLIVVRMRAVGLKAGWLQR

RRSEKKITRMVLLVVAVFVLCWMPFYIVQLISVFRKPPDPMVTQLFVILSYANSGANPIL

YGFVSDNFRRSFQRIICFRWLENGLDAEQVDYCAVALRRQTTCGQQDFPKECLASDMVFR

NGTCTSRTTTL

>Zebrafish SSTR2 Uniprot ID:E7FDL0 (gene: sstr2a)

MDTWTFMPNSNLSLPDRLVNDSFFPGNESDFGLEFYPPNGTHPGFDHTSSVVITFVYFVV

CAVGLCGNALVIYVILRYAKMKTVTNIYILNLAVADVLCMLSLPFIAIQLSLLHWPFGSA

ICRVVLTVDSMNQFTSIFFLTVMSFDRYLAVVHPIKSTKWRKPRMAKTISLGMWSVALLV

NLPIMIYSGVNAKKNEARTCTMLWPEPQNTYYTAFIFYTFFLGFFLPLIVISMCYLLIVI

KVKSSGMRVGSTKRKRSERKVTRMVSIVVVVFVLCWLPFYVFNVTSVTGTVPTTPVLKST

FDFVVVLGYANSCANPILYAFLSDNFKKSFQNVLCLKRVGGLDEIDRSDSRQDRTRMVND

IMTETHNAALLNGDLQTTI

>Zebrafish SSTR2-like Uniprot ID:E7F6Y6 (gene: sstr2b)

MDSWTYWFSPSYYSTNLSGYSLYDGLLNISEEETQRNEPEQHLTNTNTAVVTVMYFLVCV

VGLCGNALVIYVILRYAKMKTVTNIYILNLAVADILFMLSLPFIAIQLAMVHWPFGATMC

HVVITIDSLNQFTSIFCLMVMSIDRYLAVVHPMKSIKWRKPQVAKATNVAVWVVSLLVIL

PVVFYSGLITKAEGCFCSIVWPDPQGAYQTAFMIYTFLLGFFLPLMVICLCYLLIIIKVK

SSGIKVSSSKRRYSEKKVTRLVSVVVLVFVFCWLPFYIFNMTSVTGTISDTPFLRSVFAF

VVVLGYANSCANPILYAFLSENFRKSFQNVLCFKKEALSDAARSNSRLDRPQILNDPMET

QHTVINGDLQTSL

>Zebrafish SSTR5 Uniprot ID:Q6NV10 (gene: sstr5)

MATQEPIYNTSLSNQTTNSSSDPNENLLAEEESTKALAVIYLVVFIVGLTGNSLAIFVVL

RYTKMKTVTNMYILNLAVADELYILGLPFLTTHNVLSYWPFGNFLCRILMWADSISQFTS

TFCLTVMSIDRYMAVVHPIRSARWRRPSVAKVINSMVWALSCLLTLPVIIYCDVQPGLNT

CNLSWPEPRDVWSTAFILYTAILGFFCPLLVICLCYLLIVIKVKSASARAGLSKRRKSEK

KVTRMVVIIVVVFVICWLPFFMLNIFNLVVTLPENNIITGVYFLTVILTYVNSCANPLLY

GFLSDNFKRSFQKVLCIHRVNGVSDEHPNRARISRNHQEPFFPPRSFDYNDHVQSYQSIG

LDAEHCAKPENHHPGPQVISQSI

>tilapia SSTR2 Uniprot ID:I3KZY9

MDSWIFPSSPPNLSEHLLYDSFVQDNESIPHGNYTDHSFTRTSTVVITCMYFLVCAMGLCGNALVIYVIL

RYAKMKTVTNIYILNLAVADVLFMLGLPFIAIQLALVHWPFGPVLCRVVMTVDSLNQFTSIFCLMVMSID

RYLAVVHPIKSTKWRKPRMAKTINLAVWGVSLMVNLPIVIYSGVITKHDGCFCTIVWPEPQEAYYTAFMF

YTFILGFFLPLMVICLCYLCIIIKVKSSGIRVGSSKRKRSERKVTRMVSIVVAVFVFCWLPFYVFNVTSV

TGTISTTPILRSTFAFVVVLGYANSCANPILYAFLSENFKKSFQNVLCLKNVGGLDEVDRSDSRQDKSRM

MNDPTETQSTLLNGDLQTSI

>tilapia SSTR2a Uniprot ID:I3KUD5

MALDQLPFLPTSPNNSIPEPFWYDIQGNDSDLGFNISQDKEHHQDKTSSVVITFIYFMVC

AVGLCGNTLVIYVILRYAKMKTVTNIYILNLAVADVLCMMSLPFIALQLALVHWPFGEAL

CRMIMSVDSLNQFTSIFCLMVMSIDRYLAVVHPIKSTKWRKPRVAKLINLTVWGVSLLVI

LPTLIFSGLNKVPVCGIVWPEPQDVYYTAFIFYTFFIGFFLPLAVICMCYLLIIVKVKSS

GLRVGSTKRKRSERKVTRMVSIVVAVFVLCWLPFYIFNVTSVTGSIKPTSAVKSTFDFVV

VLGYANSCANPILYAFLSDNFKKSFQNVLCLKKVAGLDEVERSDSRADRSKMINEAAIIN

ANLYTHSPTLLNGELQTSI

>tilapia SSTR2-like Uniprot ID:A0A669ERT6

MNASFLYDADYNQSLVDGDFYWEENLDGFGITMAFLYLVVCVMGLAGNALVIVAILKFDKLSSSTTVYIF

NLALADGLFMVGLPFIASQNLMNRWIFGDVACKAVMVLDGINQFTSVFCLTVMSIDRYMALVDPLRFACW

RTPRCAKIVSSLLWLFSLLTILPMAVHFSTDHGFCTPDLGSDARWLGILSYTFILGFALPFTVMTGFYVA

LLLALWSQKLRTAAVNLESQQLERQVTKMVVAVVVVFGLCWLPFYTFNFCSPYQNSYAVTFFRAFEFVVL

LSYSWSCANPILYACLSDTFRRHFRTLLCPITKPSSSMQCIPDQQNLNDTVVCDGTITP

>tilapia SSTR3a Uniprot ID:I3K8H1

MELIQFALLPQEVPTATWSNSSAPSYSHFLLLSTPSEPLHSFNQDDTSFLLNSSCQNCTKSKPGSLPGLA

GIFIPLIYGIVCVVGLVGNTLVIHVVVNYTKHESVTNIYILNLAIADELFMLGLPFLAVQNALLSWPFGS

LMCRVVMTVDAINQFTSIFCLTVMSVDRYLAVVHPIRSSWWRRPHVAKAISGTVWAGSFVVVLPVVVFAD

VLKDDGNCSIVWPEPAEVWKTSFIVYTCTVGFFCPLLVICLCYLLIVIKVRSVGKRAQATSSRRKKSERK

ITRMVVVVVAVFIFCWLPFYVLNILNLLVVLPGDFRGLYFFVVVLSYANSCANPILYGFLSDNFKRGFRK

ALCRTSRRVNSNDRTGIEAQRPTEEWGGIVLQQQISEGITHVHGKESSRNEEEEEIGGIEGAIQMREICK

TSQNGNQNGVKEGSRTQVTQRGTRDQGPSLDPAEGVSSLASKRSRSPEEFLDQNSVLDISYL

>tilapia SSTR5b Uniprot ID:I3J1C6

MEDYSGIFENVNISSTYNFTADSFSNTTMLLPFNKVIVIIHTIVFIVGFLGNTLAIYVMARYAKMNTATN

IYILNLAVADELYILGIPFIGTNSVLSYWPFGEAVCKISMTADAMSQFASTFSLTLMSIDRFLFVVYPVR

SVKWRKPRLAKITSCIVWVVSFLIVLPVIIFSSVQEDFKTCNMSWPEPHDVWSAVFILYTSILSFFGPLI

VVCLCYLFIVIKVRSARERAGLTRRRKSERKVTRMVVIIVLVFVLCWLPFYTTNIVNLIHIIPEHSMDIY

FSVVILTYVNSCANPFLYAFLSKNFKESFQKVLCFQKANVARAAQVLQGRQNDTKSVQLKDLRTADSEPL

NTKNVVNGQ

>tilapia SSTR5 isoform X1 Uniprot ID:UPI00090532D2

MTSISLGAVIEVLMQSRWVGAEITEGQSNMGDASYSGLEMTAEMWENGSFASPSPGLPLFPLMFNDSDLN

DTLFNSTNSTAPDAFSGPSVAGVLIPLIYIIVCIIGLGGNTLVIHIVLHYSKIESVTNIYILNLAIADEL

FMLGLPFLAVQNTLQSWPFGSFMCRLVMTVDSINQFTSIFCLTVMSIDRYLAVVHPIRSSKWRRPQVAKV

VNGTVWALSFLVVLPVVIFANIQKAGGTCNIAWPQPANIWRAAFIIYTSTVGFFCPLLIICLCYLLIVFK

IRSSGKKVHATSTKRRKSERKVTRMVVIVVAVFVFCWLPFYALNIINLLVALPPEYQGLYYFVVVLGYAN

SCANPIVYGFLSDNFKRGFRKALCRSTRKVENHEPLEHQQQDEGRRALMPRESLRRAIRDEEDDDEEVVS

EMTEIYRIAQNGNSSYQLQSSQPLFLEKGATPGVTELASPDRRDNTGDAKGKDANGATLTVPLLLNGTKS

GSTKTLPEENLEPSASLEISYL

>tilapia SSTR5 isoform X2 Uniprot ID: I3L056

MTSISLGAVIEVLMSRWVGAEITEGQSNMGDASYSGLEMTAEMWENGSFASPSPGLPLFPLMFNDSDLND

TLFNSTNSTAPDAFSGPSVAGVLIPLIYIIVCIIGLGGNTLVIHIVLHYSKIESVTNIYILNLAIADELF

MLGLPFLAVQNTLQSWPFGSFMCRLVMTVDSINQFTSIFCLTVMSIDRYLAVVHPIRSSKWRRPQVAKVV

NGTVWALSFLVVLPVVIFANIQKAGGTCNIAWPQPANIWRAAFIIYTSTVGFFCPLLIICLCYLLIVFKI

RSSGKKVHATSTKRRKSERKVTRMVVIVVAVFVFCWLPFYALNIINLLVALPPEYQGLYYFVVVLGYANS

CANPIVYGFLSDNFKRGFRKALCRSTRKVENHEPLEHQQQDEGRRALMPRESLRRAIRDEEDDDEEVVSE

MTEIYRIAQNGNSSYQLQSSQPLFLEKGATPGVTELASPDRRDNTGDAKGKDANGATLTVPLLLNGTKSG

STKTLPEENLEPSASLEISYL

>tilapia SSTR5 isoform X3 Uniprot ID:XP_005469090.1

MGDASYSGLEMTAEMWENGSFASPSPGLPLFPLMFNDSDLNDTLFNSTNSTAPDAFSGPSVAGVLIPLIY

IIVCIIGLGGNTLVIHIVLHYSKIESVTNIYILNLAIADELFMLGLPFLAVQNTLQSWPFGSFMCRLVMT

VDSINQFTSIFCLTVMSIDRYLAVVHPIRSSKWRRPQVAKVVNGTVWALSFLVVLPVVIFANIQKAGGTC

NIAWPQPANIWRAAFIIYTSTVGFFCPLLIICLCYLLIVFKIRSSGKKVHATSTKRRKSERKVTRMVVIV

VAVFVFCWLPFYALNIINLLVALPPEYQGLYYFVVVLGYANSCANPIVYGFLSDNFKRGFRKALCRSTRK

VENHEPLEHQQQDEGRRALMPRESLRRAIRDEEDDDEEVVSEMTEIYRIAQNGNSSYQLQSSQPLFLEKG

ATPGVTELASPDRRDNTGDAKGKDANGATLTVPLLLNGTKSGSTKTLPEENLEPSASLEISYL

>Gar SSTR5-like Uniprot ID:W5NNI5

QLCARMDLAGNSAWAVSPEAFNQTLLTNISRNGTIQSVPLDGISTVLIPVIYLTVFTIGL

TGNTLAIYVVLRYTKMKTVTNIYILNLAVADELFMLGLPFLTTQNVLSYWPFGSFLCRLV

MTVDAINQFTSIFCLTVMSIDRYLAVVHPIKSTKWRRPRVAKVINASVWILSFLVVLPVI

IFSDVQDGLNTCNINWPDPNAVWSTTFIIYTSVLGFFGPLLVICLCYLLIVVKVKSSGVR

AGFTKRRKSERKVTRMVVIIVVVFVCCWLPFYILNMVNLVFIVPENNVMAGIYFFVVILS

YANSCANPLLYGFLSDNFKQSFRKVLCIYKGNGVEDGDPSLPRTEKTTFHDSFLSHRNND

FNGHMQTSK

>Gar SSTR2 Uniprot ID:W5NMK0

MDPETFSAFPNLTDPWMYDGLIMGNESTNNTGGKTEIFTDHNFDKTTTMIITFMYFVVCA

VGLCGNTLVIYVILRYAKMKTVTNIYILNLAIADELFMLGLPFFAIQLALVHWPFGQVLC

RIVMTVDGLNQFTSIFCLTVMSIDRYLAVVHPIKSTKWRKPRMAKMINLAVWGISLLVNL

PIIIYSGLLTKNNTSSCTIIWPNPPEAYYTGFMFYTFFLGFFLPLMVICLCYLLIIIKVK

SSGIRVGSSKRKKSERKVTRMVSIVVAVFVFCWLPFYVFNVTSVTGTINTTPVLKTTFDF

VVVLGYANSCANPILYAFLSENFKKSFQNVLCLKKVGGLDDIDRSDSRQDKTRMMNDATD

TQSTLLNGDLQTSI

>Gar SSTR1 Uniprot ID:W5NME7

MLPNNSSRHLSIEDGYYLMNSSGNETHSESHGSAILISFIYSVVCLVGLCGNSMVIYVIL

RYAKMKTATNIYILNLAIADELLMLSVPFLVTSSLLHHWPFGSLLCRLVLSVDAINMFTS

IYCLTVLSIDRYIAVVHPIKAARYRRPTIAKMVNLGVWIFSIMVILPIIIFSTTAPNSDG

SVACNMQMPQPQRQWMAVFVIYTFLMGFLFPVIAICMCYILIIVKMRVVALKAGWQQRKK

SERKITLMVMMVVTVFVICWMPFYIVQLVNVFVQQHNATISQLSVILGYANSCANPILYG

FLSDNFKRSFQRILCLRWMDNAAEEPIDYYATALKSRAYSVDDFQPDNMESDSTYRNGTC

TSRTTTL

>Gar SSTR3 Uniprot ID:W5N026

MAPSLSEARASVPPSLLPPPTGDALLSGLALGELFQNGSLPNSTGEGEGEGAGPGVAGVL

IPLIYFTVCIIGLGGNTLVIHIVLRYSKTESVTNIYILNLAIADELFMLGLPFLAVQNAL

SFWPFGSFMCRLVMTVDGINQFTSIFCLTVMSIDRYLAVVHPIRSCRWRKPQVAKAVNGT

VWAVSFVVVLPVVIFADVPRGGGICNIDWPQPAEVWRAAFIIYTSTLGFFGPLLVICLCY

LLIVVKVRSSGKKVRATSTKRKKSERKVTRMVVIVVAVFVFCWLPFYALNIINLVVALPE

EPKGVYFFVVVLSYANSCANPIVYGFLSDNFKRGFRKTLCRSSRKVESQEQLAAHRLQPG

QLGQPGQLGQQQQPPPPRRQGQQEEEEEEEEEEDGEATEMTEICHIAQNGNGQPEAARAL

FLPKGQRSGTSTVSPTSRNPRKAAEPNGKTPDSGTAPAPAPCLLNGATNGSMKPLPEEPG

EKNTLLEISYL

>Wrasse SSTR5 Uniprot ID:A0A3Q3FK80

MDGLNWTMISESDGVSPSQPFRSSPTSRNTSEMATPMPFDVVTAVVYTVVFIVGLLGNTL

AIYVVVRYAKMKTVTNMYILNLALADELYILGIPFLGTNSVLSNWPFGDFFCKVCMTADT

MSQFASTFCLIVMSIDRYMAVVHPIRSSKWRKPQVAKVLNGMVWVVSFLVVLPVTIYSNV

QEELNTCNITWPDPQELWAVVFILYTSILGFYGPLVIICLCYLLIVIKVRSAGVRAGLTK

RRKSERKVTRMVVIIVVVFVLCWFPFYTTNTVNLIHIIPETKATTAIYFILVILTYVNSC

ANPVLYGFLSDNFKQSFQKVICVHKPNSVCTTEKKRGRKKAPQENHVPIGTEWKTAERSG

ILQKK

>Wrasse SSTR3 Uniprot ID:A0A3Q3EHV0

MDLLGHPLFLLMFNDSDLNETLFNITNSTVPDVFSGPSVAGVLIPLIYIIVCIIGLGGNT

LVIHIVLHYSKIESVTNIYILNLAIADELFMLGLPFLAVQNTLQSWPFGSFMCRLVMTVD

SINQFTSIFCLTVMSIDRYLAVVHPIRSSKWRHPQVAKVVNTTVWALSFLVVLPVVIFAN

IQKAGGTCNIAWPQPADIWRAAFIIYTSTVGFFCPLLIICLCYLLIVFKIRSSGKKVHAT

STKRRKSERKVTRMVVIVVAVFVFCWLPFYALNILNLLVSLPSEYQGLYYFVVVLGYANS

CANPIVYCFLSDNFKRGFRKALCRSSRKREEGRTIYRIIHSNHEGNMHHNPAGCHLCTLS

FVFMFCSCWSGCRIEYFAPLQKCSLSCCLDFKNEEIKDVFQ

>Wrasse SSTR2 Uniprot ID:A0A3Q3GL87

MDSWIFPSSPPNLSEHLMYDSFMQGNESDVHGNYTDHTFNRTSTVVITCLYFLVCAVGLC

GNALVIYVILRYAKMKTVTNIYILNLAVADVLFMLGLPFIAIQLALVNWPFGPVLCRVVM

TVDSLNQFTSIFCLMVMSIDRYLAVVHPIKSTKWRKPRMAKTINLTVWGVSLMVNLPIVI

YSGVHTKHDGCFCTIVWPEPQEAYYTAFMFYTFVLGFFLPLMVICLCYLFIIIKVKSSGI

RVGSSKRKRSERKVTRMVSIVVAVFVFCWLPFYVFNVTSVTGTISTTPILRSTFAFVVVL

GYANSCANPILYAFLSENFKKSFQNVLCLKKVGGLDEVERSDSRQDKSRMINDPTETQST

LLNGDLQTSI

>Wrasse SSTR1a Uniprot ID:A0A3Q3FYX2

LAALISSIYSVVCLVGLSGNSMVIYVIFRYAKMKTATNLYILNLAVADELLMLSVPFVVT

AALLRRWPFGAALCRLVLSVDAINMFTSIYCLTVLSVDRYIAVVHPLRASRYRRPTVAKL

VNVCVWMFSLLPERRWMAAFAVYAFLMGFLFPVLAIVLCYALILSQLRVVALRAGWQQRR

SSERKITVMVTVVVSVFVVCWMPFHVVQLVGVFLQRHDPTLSQLAVVLGYANSCANPLLY

GFLSDNFRRSFQRILCVRWMEAPEEPLDYLDYFSTALKSRRLSLDQDQEEQDRPRPHNTC

SRITTVVPA

>Pike SSTR5 Uniprot ID:A0A6Q2YDV8

ILTNSAPLHCFLSGIDAWGFHATVAGVLIPLIYMAVCVVGLVGNTLVIYIVLHYLRAESV

TNIYILNLAIADELFMLGLPFLAVQNTLLSWPFGSLMCRVVMTVDAINQFTSIFCLTVMS

TDRYLAVVQPIRASWWRRPRVAKAVNATVWAVSFVVVLPVVAFADVLEDDGNCSIVWPEP

AEVWKASFIIYTCTVGFFGPLLVICLCYLLIVVKVQTARATSSRRRKSERKITRMVVVVV

AVFVLCWLPFYLLNIINLLVLLPGEFRGLYYFVVVLSYANSCANPILYGFLSDNFKRGFR

KALCRASRRVDNHERGEASEQQRPTEVWGGIELEPRDGPRVTEGQGGGEEEKEEGAGKET

IEGAAQTREISKIVPNSRGGQVENRILSTQGGQVEMVNLGQRDKSVELSETGSGSGPTVA

LPIVENGSKNKSDHPRPEETADKGSKLEISCL

>Pike SSTR1b Uniprot ID:A0A3P8ZYE9

AKMDNIIIPSIYALVCCVGLTGNAMVIYVILKYAKMKTATNIYILNLAIADELFMLSVPF

LATSAAVRHWPFGSLMCRLVLSVDGINMFTSIFCLTVLSVDRYVAVVHPIKAARYRRPTV

AKVVNVCVWGFSLLVILPIIIFADTVPAQDGGVDCNFLWPEAVWSEAFVVYTFVLGFLLP

VGAICLCYCLMVARMRAVGLKAGWLQRRRSEKKITRMVLCVVAVFVLCWMPFYIIQLVSV

FHRPPDPMVTQLFVILSYANSGANPILYGFVSDNFRRSFQRIVCFRWLESGLDGEQVDYR

AVALKRQATSGQKDFPKEHQVSDMVFRNGTCTSRTTTL

>Pike SSTR5-like Uniprot ID:A0A3P8YRY2

MWENSTVPIPYINPPLSLLLCNDTLFNDTFINCTNSTTTTSPEMPAGPSVAGVLIPLIYI

VVCFIGLGGNTLVIHIVLHYSKTESVTNIYILNLAIADELFMLGLPFLAIQNTLQTWPFG

SFMCRLVMTVDSINQFTSIFCLTVMSIDRYLAVVHPMRSSRWRRPQVAKVVNCTVWAISF

LVVLPVVIYANVQKTGGTCNISWPKPAHIWRAAFIIYTSTVGFFCPLLIICLCYLLIVFK

IRSSGKKVHATSTKRRKSERKVTRMVVIVVAVFVFCWLPFYALNIINLLVSLPSEYQGLY

YFVVVMGYANSCANPIVYGFLSENFKRGFRKALCRSSRKVESHELTERQQQQEERRRVLM

PRESLRRVVQDEEEEDDEEREDVTEMTEICRITQNGNRQPESCQALLTPKTPAPGASEQI

DSLEKKAKAGDLCGKDPVLGPLASLHNGDKNGSVKPLPEEPVENPSLEISYL

>Pike SSTR1 Uniprot ID:A0A3P8XB42

MIPNNSFRNLTLEDVFFLMNNSSGNETHSESHGSAILISFIYSVVCLVGLCGNSMVIYVI

FRYAKMKTATNIYILNLAIADELLMLSVPFLVTSSLLHHWPFGSLLCRLVLSVDAINMFT

SIYCLTVLSIDRYIAVVHPIKASRYRRPTIAKIVNFGVWIFSILVILPIIIFSTTVPNLD

GSVACNIQMPEPMNQWMAVFVIYAFLMGFLFPVIAICMCYILIIAKMRVVALKAGWQQRK

KSERKITVMVVMVVTVFVICWMPFHIVQLVNVFVEHHNATLMQLAVILGYANSCANPILY

GFLSDNFKHSFQRILCLRWMENATEEPIDYYATALKSRGYSVDEFQPDNIERDSTFRNGT

CTSRTTTL

>Pike SSTR2 Uniprot ID:A0A3P8ZKP3

MDAWPLLPSPPNLSLADPLYYDSYFPGNESDLGSRNDTPDEIHHGFDKTSSVVITFIYFT

VCAVGLTGNALVIYVILRYAKMKTVTNIYILNLAVADVLCMLSLPFIAVQLALVRWPFGS

VLCRLVMTADSLNQFTSIFCLTVMSIDRYLAVVHPIKSTKWRKPRVAKIINLTVWGVSLL

VNLPIMIFSGLTPNRNHAWVCTIVWPEPQEAYRTAFMFYTFFLGFFLPLTVICLCYLLII

IKVKSSGMRVGSTKRKRSERKVTRMVSFVVAMFVLCWLPFYVFNVTSVIGTIKTTSVLKS

TFEFVVVLGYANSCANPILYAFLSDNFKKSFQNVLCLKRVSGLDEVERSESRMDRTRTVN

DATAETHNAALLNGELQTSI

>Chicken SSTR2 Uniprot ID:Q58G84

MDLEYELPNATTFWFSPASPFDNFSVEAPTNASQNATGQHFDLTSNAILTFIYFVVCIIG

LCGNTLVIYVILRYAKMKTITNIYILNLAIADELFMLGLPFLAMQVALVHWPFGKAICRI

VMTVDGINQFTSIFCLTVMSVDRYLAVVHPIKSAKWRRPRTAKMINMAVWGVSLLVIMPI

MIYAGVQQNHGRSSCTIIWPGESGAWYTGFIIYAFILGFLVPLTIICLCYLFIIIKVKSS

GIRVGSSKRKKSEKKVTRMVSIVVAVFIFCWLPFYIFNVSSVSVLIVPTPVLKGMFDFVV

VLSYANSCANPILYAFLSDNFKKSFQNVLCLVKVSGMDDADRSDSKQDKSRLNETTETQR

TLLNGDLQTSI

>Chicken SSTR1_A9UGZ3

MLPDGTCTRLPGGAGSHSGGSHSGASGALEEAAARGMDSGGRNSSGAPNSTLSESQGSAI

LISFIYSVVCLVGLCGNSMVIYVILRYAKMKTATNIYILNLAIADELLMLSVPFLVTSTL

LHHWPFGSLLCRLVLSVDAINMFTSIYCLTVLSVDRYIAVVHPIKAARYRRPTVAKMVNL

GVWVLSILIILPIIIFSNTAANSDGTVACNMLMPEPTQRWLVVFVVYTFLMGFLLPVVAI

CLCYILIIAKMRMVALKAGWQQRKRSERKITLMVMMVVMVFVICWMPFYIVQLVNVFVEQ

DDATISQLSVILGYANSCANPILYGFLSDNFKRSFQRLLCLSWMDNAPEEPIDYYATALK

SRAYSVEDFPPDNLESGSMYRNGTCTSRITTL

>Chicken SSTR3 Uniprot ID:Q4ZJF2

MDTSAFSLPTPTVSEEGNASGSWAGFTTPNSSTTTTSPGVVVSGVLIPMVYLIVCVVGLI

GNSLVIYVVLRHSVSESVTNVYILNLALADELFMLGLPFLAAQNALSYWPFGSFMCRLVM

AVDAINQFTSIFCLTVMSVDRYLAVVHPGKSSKWRTARVAKAVSATVWMLSSIVVLPVVV

FSDVPLGMNTCHIQWPEPASVWRAGFIIYTATLGFFGPLLVICLCYLLIVVKVRSSGRRV

RALSSKHKLSERRVTRMVVTVVAVFVLCWLPFYVLNIINVVCPLPEEPSLFGVYFLVVVL

PYANSCANPIIYGFLSYRFKQGFRRAIFRPSRRVQSQEVPECPPEKSDDGGEEKEISKIT

QNGNDREERPLSSRAGERNGQKPLPEEPVGCEKSSKLHVSYL

>Chicken SSTR4 Q4PLA6

MSTNAEHLPPWAASAVPPNASTAWMEGPSARGGRVGELAGMVVIQCIYALVCLLGLLGNS

LVIFVILRYTKMKTATNIYLLNLAIADELFMLSIPFVATSAALHHWPFGRALCRTVLGVD

GLNMFTSVFCLTVLSLDRYIAVVHPLRAATYRRPRVAKMVNGAVWLLSLLVASPIPIFAG

TAVTRDGQAVACNLLWPSPAWSAAFVVYTTLLGFLLPVLAMGLCYLLIVGKMRAVAQRVG

WQQRRRSEGKLTRLVLMVVAMFVVCWMPFYVVQLVNLLLPGRLDATVNNACLILSYSNSC

ANPILYGFLSENFRHSFHGVLRRCLDASLCCCPPEARAVGDEEEEEEEEPLDYRAVPRSD

PKGKGCMCPPLPCQQEPARPEPFCKPGTLLAKTTTF

>Chicken SSTR5 Uniprot ID:F6RCI2

MDSSSSDLNSSLLTNTTENGTLSEQLPFKYIHKVLIPIFYILVCAVGLSGNTLVIYVVLR

YAKMKTVTNIYILNLAVADVLFMLGLPFLATQNAISYWPFGSFLCRLVMTVDGINQFTSI

FCLTVMSMDRYLAVVHPIKSTKWRRPRVAKLISMTVWTFSFLVVLPVFIFSDVQEDFHTC

NMNWPEPVNIWSAAFIIYTSVLGFFGPLLVICLCYLLIVIKVKSSGIRVGSTRRRRSERK

VTRMVVIIVVVFVFCWLPFYMMNIVNLIFILPEDPVLVGVYFFVVVLSYANSCANPILYG

FLSDNFKQSFQKVLCLRKGNGVEDGDPIEHRQENSSRLQESMLTQRNIEFNGHMQTSKV

>Sperm Whale SSTR2 Uniprot ID:A0A455C1M3

MDPAYELLNGTQPWLSSPFDLNGSVATANISNQTEPYYDLTSNAVLTFIYFVVCIVGLCG

NTLVIYVILRYAKMKTITNIYILNLAIADELFMLGLPFLAMQVALVHWPFGKAICRVVMT

VDGINQFTSIFCLTVMSIDRYLAVVHPIKSAKWRRPRTAKMINVAVWGVSLLVILPIMIY

AGLRSNQWGRSSCTINWPGESGAWYTGFIIYAFILGFLVPLTIICLCYLFIIIKVKSSGI

RVGSSKRKKSEKKVTRMVSIVVAVFIFCWLPFYIFNVSSVSVAISPTPALKGMFDFVVVL

TYANSCANPILYAFLSDNFKKSFQNVLCLVKVSGTDDGERSDSKQDKSRLNETTETQRTL

LNGDLQTSI

>Sperm Whale SSTR5 Uniprot ID:A0A2Y9EE72

MEPLFPASPLTSWNASSAATGSGGENGTLAGLVPSPGARAVVVPVLYLLVCTVGLGGNAL

VIYVVLRHAKMKTVTNIYILNLAVADVLLMLGLPFVATQNAISYWPFGPVLCRLVMTLDG

INQFTSIFCLTVMSVDRYLAVVHPIRSARWRRPRVAKLASAAVWAFSLVMSLPLVVFADI

QEGWNTCNLSWPEPVGLWGAIFIIYTSVLGFFGPLLVICLCYLLIVVKLKASGMRVGSTR

RRSERKVTRMVVVVVLVFVGCWLPFFIVNIVNLAFVLPEEPASAGAYFFVVVLSYANSCA

NPLLYGFLSDNFRQSFRKVLCLRKSYGTEDADATEPRPGQSSRLQEAMLPTRSCKANGLM

QTSKL

>Sperm Whale SSTR3 Uniprot ID:A0A2Y9EZ24

MDTPGYPSLVPTPSEPWNASSAWPLDAILGNASSARSAAGLAVSGILIPLVYLVVCVVGL

LGNSLVIYVVLRQTASPSVTNIYILNLALADELFMLGLPFLAAQNALSYWPFGSLMCRLV

MAVDGINQFTSIFCLTVMSVDRYLAVAHPTRSARWRTAPVARTVSAAVWVASAVVVLPVV

VFSGVPRGMSTCHMQWPEPAAAWRAGFIIYTAALGFFGPLLVICLCYLLIVVKVRSAGRR

VWAPSCQRRRHSERRVTRMVVAVVALFVLCWMPFYVLNIINVVCPLPEEPAFFGLYFLVV

ALPYANSCANPILYGFLSYRFKQGFRRVLLRPSRRVRNQEPPLGPPEKTEEEEDEEEDGG

GEDGQEGAGKLEELKEMNGRVNRITQPGPSGQEQPPSGPTRKEHQFLPQEPSAAEKSGTL

HISYL

>Sperm Whale SSTR1 Uniprot ID:A0A2Y9FK33

MFPNGTASSPSSPSPSPGSCGEGGGSRGPGAGAADGMEEPGRNASQNGTLSEGQGSAILI

SFIYSVVCLVGLCGNSMVIYVILRYAKMKTATNIYILNLAIADELLMLSVPFLVTSTLLR

HWPFGALLCRLVLSVDAVNMFTSIYCLTVLSVDRYVAVVHPIKAARYRRPTVAKVVNLGV

WVLSLLVILPIVVFSRTAANSDGTVACNMLMPEPAQRWLVGFVLYTFLMGFLLPVGAICL

CYVLIIAKMRMVALKAGWQQRKRSERKITLMVMMVVMVFVICWMPFYVVQLVNVFAEQDD

ATVSQLSVILGYANSCANPILYGFLSDNFKRSFQRILCLSWMDNAAEEPVDYYATALKSR

AYSVEDFQPENLESGGVFRNGTCTSRITTL

>Sperm Whale SSTR4 Uniprot ID:A0A2Y9FUR2

MSAPPTPPPGEEERLETAWPPGDQRQRHPGGDGGGGGGGDRVAIQCVSAPVCEVVNALVI

FVTLRSAKMKTATDIYLLNLAVAHELFMLSVPFVGSSAAPRPWPFGPALCRAVLSVDGLN

AFPSVFCLAGLSVDRYLAVVHPLRAATCRRPGVARLVSXGVWLSSSLVTLTIAAFADTRP

ARGGRAVAYNLRWPHPAWSAVFVVCTFLLGFXLPVLAVSLCYVLIVGKTWALALPAGWQQ

RKRSEEKITWLVLTVVAVFVLCWLPFYAVQLLNLSVTGLDATVNHVSLILSYANSCANPI

LYGFLSDNFRRSFQRVLCLRCCLLDASGGADQEPLDYCATALKSRGGAGAACPPLPSQQE

PRQPEPSRKQAPLSRTTTF

>Tounge Sole SSTR2 Uniprot ID:A0A3P8X3M9

MALDPWPFLSTPPNTSIPEPLLYDSFLPGNESDLDLNISGTREPHQDRTSSVVITFIYFM

VCAVGLCGNTLVIYVILRYAKMKTVTNIYILNLAVADVLCMMSLPFVALQLALVRWPFGE

ALCRVIMTVDSLNQFTSIFCLMLMSIDRYLAVVHPIKSTKWRKPRVAKLINLMVWGVSLL

VILPTMIFSGLNKVPVCGILWPEPQDVYYKAFIFYTFSVGFFLPLVVICMCYLLIIVKVK

SSGMRVGSTKRKRSERKVTRMVSIVVAVFVLCWLPFYIFNVTSVTSFIHPTSAIKSTFDF

VVVLGYANSCANPILYAFLSENFKKSFQNVLCLKKVAGLDEIERSDSRADRSRMMVNDAI

ISSANLETHNTALLNSELQTSI

>Tounge Sole SSTR5-like Uniprot ID:A0A3P8W8E7

MGLTPSSCSMDGYNWTSPSDNVSMSQSNPGLSTRNSSDELEPLPFSVDTAVIYTIVFIVG

FLGNSLVIYVVIRYAKMKTVTNMYILNLALADELYILGIPFLGTNSVLSYWPYGDFFCKV

CMTADAMSQFSSTFCLTVMSIDRYLAVVYPIRSNKWRKPRMAKIFNGMVWVVSFLVVLPV

TFYSDVQDELNTCNISWPEPVDLWSIVFILYTSILGFFGPLFVISICYLLIVVKVRSAGV

RAGLTKRRKSERKVTRMVVIIVLVFVICWLPFFIANTVNLFYIIPESRTTATVYFFLVIL

TYVNSCANPILYGFLSENFKQSFQKVFCFQKPNGVGVTEQMGAQTSPKVKLVRLPPTDLA

CSLHHKTLHVQLHERVIFIFYSFLLPNINIQFLKLNAEFNISLGRTC

>Tounge Sole SSTR3 Uniprot ID:A0A3P8X2W9

MGDTWVPGLQPWENNSWAGQSQSLSLLLMLNNSILNDTLLCCSNFTNSTGADIPSGLSLG

AVLIPLVYIVVCIVGLGGNTLVIHIVLHYSKTESVTNIYILNLAIADELFMLGLPFLAVQ

NTLQLWPFGPFMCRLVMTVDSINQFTSIFCLTVMSIDRYLAVVHPILSSKWRRPKVAKIV

NGTVWALSFLVVLPVVVFANIQKGGGTCNIAWPQPANIWSTAFIIYTSTVGFFCPLLIIC

LCYLLIIFKIRSSGKKVHATSIKRRKSERKVTRMVVIVVAIFVVCWLPFYAVNIINVLVS

LPSEYHGLYYFVVVLGYANSCANPIIYGFLSDNFKRGFRKALCRSTRKEQEEEEDEDVSE

MTEIYRIADNGNSKMHLR

>Medaka SSTR1b Uniprot ID:A0A3B3HD21

HTAVSTFHLLTDQLSFYLDYEDGYHEENASKIIIPSIYALVCCLGLTGNAMVIYVILKYA

KMKTATNIYILNLAIADELFMLSVPFLATSAAIRHWPFGSVMCRLVLSVDGINMFTSIFC

LTVLSVDRYVAVVHPIKAARYRRPTVAKVVNVCVWGLSFIVILPIIIFADTVPAEDGGVD

CNFLWPEAFWSEAFVVYTFLLGFLLPVGAICFCYCLIVARMRAVGLKAGWLQRRRSEKKI

TRMVLLVVAVFVLCWMPFYIVQLVSVFHRPPNPMVTQLFVILSYANSGANPILYGFVSEN

FRRSFQRIVCFRWLEPGLDEEQVDYCAVALKRKTTRNHLEIPKDYMASDVLFRNGTYTSR

TTTL

>Medaka SSTR2 Uniprot ID:A0A3P9HMT4

MESWAFPSCSPNCSEHVMYDSLVQGNDSSQSRNHTDDSFSRTSTVVITCLYFLVCAVGLC

GNALVIYVILRYAKMKTVTNIYILNLAVADVLFMLGLPFIALQLALVHWPFGPVLCRIVM

TVDSLNQFTSIFCLMVMSIDRYLAVVHPIRSTKWRKPRVAKTINVAVWGVSLLVNLPIVI

YSGIIMKHDSCFCTIVWPEPQEAYYTAFMFYTFILGFFLPLMVICLCYLFIIIKVKSSGI

RVGSSMRKRSERKVTRMVSIVVAVFVFCWLPFYVFNVTSVTGTISTTPFLRSTFAFVVVL

GYANSCANPILYAFLSENFKKSFQNVLCLKKVGGLDEVERSDSKQDKPRLMNDPTETQST

LLNGDLQTSI

>Medaka SSTR3

MDVSTWENGSLAGPSPGLSLGLLLFNESDLNETLFNSTNSTFPDFFPGPSVAGVLIPLIY

IVVCVIGLGGNTLVIHIVLHYSKIESVTNIYILNLAIADELFMLGLPFLAVQNTLQSWPF

GSFMCRLVMTVDSINQFTSIFCLTVMSIDRYLAVVHPIRSSKWRRPQVAKVVNGTVWALS

FLVVLPVVIFANIQKAGGTCNISWPQPANIWRAAFIIYTSTVGFFCPLLIICLCYLLIVF

KIRSSGKKVHATSTKRRKSERKVTRMVVIVVAVFVFCWLPFYALNIINLLVLLPSEYQGL

YYFVVVLGYANSCANPIVYGFLSDNFKRGFRKALCRSTRRVENHEPVEHQQQQESLRRDV

SEMTEIYRITQNGNGSFQPQSSQPPFAEKGATPGVTELSSANENAGDGKGKEPVTGATVP

LLLNGAKNGNAKNLPEENPEQSTSLEISYL

>Medaka SSTR5 Uniprot ID:A0A3P9MAF8

MMNSSASWYGMLMNINHVSFLTFRSFELCPPSLRMNHHNWNETLFSSSGPVDTITNFTNE

TDPMPINKTAAITYPIVFIVGFLGNTLAIYVVGRYGKLNNVTNIYIINLAIADELYVLGI

PFILTNNVFSYWPFGDFFCKMFMTTDAMCQFASTFCLTLMSIDRFMAVVYPMRSAKWRTP

PKAKIFSGIVWIMSFLTVLPVTIYSHVQDNIKTCNLSWPEPHGSWSMVFILYTSILGFFS

PLIIISLCYLLIVIKVKSASVRAGLTKRRRSERKVTRMVVIIVFVFVLCWLPFFTANIVN

LFYVIPENETTAAIYFSLVILTYVNSCANPFLYGFLSENFEKNMKEALKCGKLNNAASPK

SGRQNAAKVNHDVYAVTHKSDMHGSKENSPTCFYKKGKVCEIIKKRSVKK

>Yangtze river dolphin SSTR2 Uniprot ID:A0A340X5U1

MDLAYELLNGTQPWLSSPFDLSGSVATANISNQTEPYYDLTSNAVLTFIYFVVCIIGLCG

NTLVIYVILRYAKMKTITNVYILNLAIADELFMLGLPFLAMQVALVHWPFGKAICQVVMT

VDGINQFTSIFCLTVMSVDRYLAVVHPIKSAKWRRPRTAKMINVAVWGVSLLVILPIMIY

AGLRSNQWGRSSCTINWPGESGAWYTGFIIYAFILGFLVPLTIICLCYLFIIIKVKSSGI

RVGSSKRKKSEKKVTRMVSIVVAVFIFCWLPFYIFNVSSVSVAISPTPALKGMFDFVVAL

TYANSCANPILYAFLSDNFKKSFQNVLCLVKVSGTDDGERSDSKQDKSRLNETTETQRTL

LNGDLQTSI

>Yangtze river dolphin SSTR5 Uniprot ID:A0A340WX78

MEPLFPASPLTSWNASSAATGRGGENGTLAGLVPSPGARAVVVPVLYLLVCTVGLGGNAL

VIYVVLRHAKMKTVTNIYILNLAVADVLLMLGLPFVATQNAISYWPFGPVLCRLVMTLDG

INQFTSIFCLTVMSVDRYLAVVHPIRSARWRRPRVAKLASATVWAFSLVMSLPLVVFADI

QEGWNTCNLSWPEPVGLWGAIFIIYTSVLGFFGPLLVICLCYLLIVVKLKASGMRVGSTR

RRSERKVTRMVVVVVLVFVGCWLPFFIVNIVNLAFVLPEEPASAGAYFFVVVLSYANSCA

NPLLYGFLSDNFRQSFRKVLCLRKSYGTEDADATEPRPGQSSRLQEAMLPARSCEANGLM

QTSKL

>Yangtze river dolphin SSTR3 Uniprot ID:A0A340X4Q6

MDTPGYPSPVPTPSEPWNASSAWPLDTILGNASAVQNAVGLAVSGILIPLVYLVVCVVGL

LGNSLVIYVVLRQTASPSVTNIYILNLALADELFMLGLPFLAAQNALSYWPFGALMCRLV

MAVDGINQFTSIFCLTVMSVDRYLAVAHPTRSARWRTAPVARTVSAAVWLASAVVVLPVV

VFSGVPRGMSTCHMQWPEPAAAWRAGFIIYTAALGFFGPLLVICLCYLLIVVKVRSAGRR

VWAPSCQRRRHSERRVTRMVVAVVALFVLCWMPFYVLNIINVVCPLPEEPAFFGLYFLVV

ALPYANSCANPILYGFLSYRFKQGFRRVLLRPSRRVRNQEPPLGPPEKTEDEEDEGEDGG

GEAGQEGAGEHEGPKETNGRVTRITQPGPSGQEQPPSGPTRKECQFLPQEPSAAENSGTL

HISYL

>Yangtze river dolphin SSTR4 Uniprot ID:A0A340XEX0

MSAPPTPPPGGEERLKTAAAGTVAIQCVSALVCVVGNALVIFVILRYAKMKTATNNYVLN

LAVADELFXLSVPFVASXLNHWPFGPALCRAVLSVDGLSSFPSVFCLYVLSVDRYLAVVH

PLRAATCRRPGVARLVSXGVXLSSSLVTQTIAAFADTRPAGGGRAVACNLHWPHPAWLAV

FVVCTFLLGFLLPILAVCLCYVLVVGKMWALALLAGWQQRKRSKKITWLNLTVAAVFVLC

WLPFYTVXXXLNLSVTGLDARVHRVSLILASHSHANSCANPILYGFLSDSFLRSFQGVLC

LCCCLLDASGGAEQEPLNYCATALKSRGGAGAACPPLPCQQEPRQPEPSRKQVPLTRSTT

F

>Yangtze river dolphin SSTR1 Uniprot ID:A0A340XWY4

MFPNGTASSPSSPSPSPGSCGEGGGSRGPGAGAADGMEEPGRNASQNGTLSEGQGSAILI

SFIYSVVCLVGLCGNSMVIYVILRYAKMKTATNIYILNLAIADELLMLSVPFLVTSTLLR

HWPFGALLCRLVLSVDAVNMFTSIYCLTVLSVDRYVAVVHPIKAARYRRPTVAKVVNLGV

WVLSLLVILPIVVFSRTAANSDGTVACNMLMPEPAQRWLVGFVLYTFLMGFLLPVGAICL

CYVLIIAKMRMVALKAGWQQRKRSERKITLMVMMVVMVFVICWMPFYVVQLVNVFAEQDD

ATVSQLSVILGYANSCANPILYGFLSDNFKRSFQRILCLSWMDNAAEEPVDYYATALKSR

AYSVEDFQPENLESGGVFRNGTCTSRITTL

>Alligator SSTR5 Uniprot ID:A0A3Q0GL42

MDALYYPSAFDMETSSQDMNFSLLNNVTENETLATLPPSKDIHKVLIPIIYLLVCAIGLS

GNTLVIYVVLRYAKMKTVTNIYILNLAVADVLFMLGLPFLATQNAISYWPFGSFLCRLVM

TADGINQFTSIFCLTVMSMDRYLAVVHPIKSTKWRRPRVAKLISITVWTFSILVVLPVII

YSDVQEDFQTCNMNWPDPVNIWSAAFIIYTSVLGFFGPLLVICLCYLLIVIKVKSSGIRV

GSTRRRRSERKVTRMVVIIVVVFVFCWLPFYILNIVNLIFILPEDPVLLGVYFFVVVLSY

ANSCANPILYGFLSDNFKQSFQKVLCLRKGNGIEDGDPIEHRQENSSRLQESMLTQRNIE

FNGHMQTSKV

>Alligator SSTR1 Uniprot ID:A0A1U7R0Z7

MDSGGRNSSGAPNSTTLSEAQGSAILISFIYSVVCLVGLCGNSMVIYVILRYAKMKTATN

IYILNLAIADELLMLSVPFLVTSTLLHHWPFGSLLCRLVLSVDAINMFTSIYCLTVLSVD

RYIAVVHPIKAARYRRPTVAKMVNLGVWVLSILIILPIIIYSNTAANSDGTVACNMLMPE

PTQKWLVVFVVYTFLMGFLLPVVAICLCYILIIAKMRMVALKAGWQQRKRSERKITLMVM

MVVMVFVICWMPFYIVQLVNVFNVEQDDATISQLSVILGYANSCANPILYGFLSDNFKRS

FQRLLCLSWMDNAAEEPVDYYATALKSRAYSVEDFPPDRLESGSVYRNGTCTSRITTL

>Alligator SSTR4 Uniprot ID:A0A1U7RCF9

MSTDANHLPTGSQEVSGALWTPSSWTASDVPPNTSASLPEQPGEEEWGGNVGEIAGMVVI

QFIYALVCLLGLVGNSLVIFVILRYAKMKTATNIYLLNLAIADELFMLSIPFVATSAALH

HWPFGRALCRTVLGVDGLNMFTSVFCLTVLSLDRYIAVVHPLRAATYRRPRVAKMVNGGV

WLLSLLVASPIPIFAGTATTRDGHAVACNLLWPSPAWSAAFVVYTTLLGFLLPVLAMGLC

YLLIVGKMRAVAQRVGWQQRRRTEGKLTRLVLIIVAMFVVCWMPFYVVQLVNLLLPGRLD

ATVNNASLILSYSNSCANPILYGFLSENFRHSFQGVLRRCFDASFCCCPVDLDEAEEEEE

EPLDYCANPRGDEKNKGCMCPTLPCQQEPVHPEPCCKPGTLLTKTTTF

>Alligator SSTR3 Uniprot ID:A0A1U7S5M0

MDTPAFSFPTPMVPEEGNTTINWIEPISGNLSIAASPGAVISGVLIPLVYLIVCVVGLVG

NSLVIYVVLCHSVSESVTNVYILNLALADELFMLGLPFLAAQNALSYWPFGSFMCRLVMA

VDAINQFTSIFCLTVMSVDRYLAVVHPGKSSKWRTARVAKVVSTTVWVLSSVVVLPVVVF

SDVPLGMSTCHIQWPEPASVWKAGFIIYTAALGFFGPLLVICLCYLLIVIKVRSSGRRVQ

ALSAKRKLSERRVTRMVVAVVAVFVLCWLPFYVLNIINVVCPLPEEPSLFGVYFLVVVLP

YANSCANPIIYGFLSYRFKQGFRRAILRPSRRVQSQEVAACPLEKSEDEEEEEEEEARRV

SKIAQNGNGQQECPLSSGAGGSSEQKPLPEDPVSCEKTSVLNISYL

>Alligator SSTR2 Uniprot ID:A0A1U8DA43

MDGEPDLPNITQFWYSSASPFHNLSTETPANASQNVTHNHFDLTSNAVLTLIYFVVCIIG

LCGNTLVIYVILRYAKMKTITNIYILNLAIADELFMLGLPFLAMQVALVHWPFGKAICRV

VMTVDGINQFTSIFCLTVMSIDRYLAVVHPIKSAKWRRPRTAKMINVAVWGLSLLVIMPI

MIYAGVQSNHGRSSCTIIWPDESGAWYTGFIIYAFILGFLVPLTIICLCYLFIIIKVKSS

GIRVGSSKRKKSEKKVTRMVSIVVAVFIFCWLPFYIFNVSSVSVLIVPTPALKGMFDFVV

VLSYANSCANPILYAFLSDNFKKSFQNVLCLVKVSGMDDADRSDSKQDKSRLNETTETQR

TLLNGDLQTSI

>Panda SSTR2_D2HUB6

MDLEYELLNESRTWPSPPFDLDGSVVAANSSNQTEPYYDLTSNAVLTFIYFVVCIIGLCG

NTLVIYVILRYAKMKTITNIYILNLAIADELFMLGLPFLAMQVALVHWPFGKAICRVVMT

VDGINQFTSIFCLTVMSIDRYLAVVHPIKSAKWRRPRTAKMVNVAVWGVSLLVILPIMIY

AGLRSNQWGRSSCTINWPGESGAWYTGFIIYTFILGFLVPLTIICLCYLFIIIKVKSSGI

RVGSSKRKKSEKKVTRMVSIVVAVFIFCWLPFYIFNVSSVSVAISPTPALKGMFDFVVVL

TYANSCANPILYAFLSDNFKKSFQNVLCLVKVSGTDDGERSDSKQDKSRLNETTETQRTL

LNGDLQTSI

>Panda SSTR5 Uniprot ID:D2HDY0

MEPLFPAPTLAGWNTSSAATSAGGENGTLAGLAPSPGARAVVVPVLYLLVCAVGLGGNAL

VIYVVLRHAKMKTVTNIYILNLAVADVLLMLGLPFLATQNAVSYWPFGPVLCRLVMTLDG

INQFTSIFCLTVMSVDRYLAVVHPIRSTRWRRPRVAKLASAAVWAFSLLMSLPLVVFADI

QEGWNTCNLSWPEPVGLWGAVFIIYTSVLGFFGPLLVICLCYLLIVVKVKASGVRVGASR

RRSERKVTRMVVVVVVVFVGCWLPFFIVNIVNLAFVLPEEPASAGAYFFVVILSYANSCA

NPVLYGFLSDNFRQSFRKVLCLRKGHGAEDADATEPQPDKGSRLQEATLPVRGSEANGVM

QTSRL

>Panda SSTR1 Uniprot ID:D2HTA1

MFPNGTASSPSSPSPSPSSCGEGGGSRGPGAGAADGMEEPGRNASQNGTLSEGQGSAILI

SFIYSVVCLVGLCGNSMVIYVILRYAKMKTATNIYILNLAIADELLMLSVPFLVTSTLLR

HWPFGALLCRLVLSVDAVNMFTSIYCLTVLSVDRYVAVVHPIKAARYRRPTVAKVVNLGV

WVLSLLVILPIVVFSRTAANSDGTVACNMLMPEPAQRWLVGFVLYTFLMGFLLPVGAICL

CYVLIIAKMRMVALKAGWQQRKRSERKITLMVMMVVMVFVICWMPFYVVQLVNVFAEQDD

ATVSQLSVILGYANSCANPILYGFLSDNFKRSFQRILCLSWMDNAAEEPVDYYATALKSR

AYSVEDFQPENLESGGGVFRNGTCTSRITTL

>Panda SSTR3 Uniprot ID:G1MNJ6

MDTPGYPVSVPTTLEPGNTSSAWLLDATLGNASAAPSVAGLAVSGVLIPLVYLVVCVVGL

LGNSLVIYVVLRHTASPSVTSVYILNLALADELFMLGLPFLAAQNALSYWPFGSLMCRLV

MAVDGINQFTSIFCLTVMSVDRYLAVVHPTRSARWRTAPVARTVSVAVWVASAVVVLPVV

VFSGVPHGMSTCHMQWPEPAAAWRAGFIIYTAALGFFGPLLVICLCYLLIVVKVRSAGRQ

VRAPSCQRRRHSERRVTRMVVAVVALFVLCWMPFYVLNIVNVVCPLPEEPAFFGLYFLVV

ALPYANSCANPILYGFLSYRFKQGFRRVLLRPSRRVRSQEPPAGPPGGKTVEDKGEEEDR

EEDGREGAGKQGEGKEVNGRVSLIVQPGTSRQEKPPSGKASKDKQFLPQEASAADKPGAL

HISYL

>Panda SSTR4 Uniprot ID:G1ML85

MSASPTLPPRGELETACSPAANASCAPAGEEEAAAGTRDTGTGGMVAIQCVYALVCLVGL

VGNALVIFVILRYAKMKTATNIYLLNLAIADELFMLSVPFVASSAALRHWPFGSVLCRAV

LSVDGLNMFTSVFCLTVLSVDRYVAVVHPLRAATYRRPSVAKLINLGVWLASLLVTLPIA

IFADTRPARGGPAVACNLHWPHPAWSAVFVVYTFLLGFLLPVLAIGLCYLLIVGKMRAVA

LRAGWQQRRRSEKKITRLVLMVVAVFVLCWMPFYVVQLLNLFVTSLDATVNHVSLILSYA

NSCANPILYGFLSDNFRRSFQRVLCLRCCLLDAAGGAEEEPLDYYATALKNRGGAEWICS

PLPCQQEPLQPEPSRKQVPLTRTTTF

>Goat SSTR1 Uniprot ID:A0A452DS64

MFPNGTASSPSSPSPSPGSCGEGGGGRGPGAGAADGMEESGRNASQNGTLSEGQGSAILI

SFIYSVVCLVGLCGNSMVIYVILRYAKMKTATNIYILNLAIADELLMLSVPFLVTSTLLR

HWPFGALLCRLVLSVDAVNMFTSIYCLTVLSVDRYVAVVHPIKAARYRRPTVAKVVNLGV

WVLSLLVILPIVVFSRTAANSDGTVACNMLMPEPAQRWLVGFVLYTFLMGFLLPVGAICL

CYVLIIAKMRMVALKAGWQQRKRSERKITLMVMMVVMVFVICWMPFYVVQLVNVFAEQDD

ATVSQLSVILGYANSCANPILYGFLSDNFKRSFQRILCLSWMDNAAEEPVDYYATALKSR

AYSVEDFQPENLESGGVFRNGTCTSRITTL

>Goat SSTR2 Uniprot ID:A0A452DWP0

MDLASELNETQPWLTSPFDLNGSVGAANISNQTEPYYDLASNVVLTFIYFVVCIVGLCGN

TLVIYVILRYAKMKTITNIYILNLAIADELFMLGLPFLAMQVALVHWPFGKAICRVVMTV

DGINQFTSIFCLTVMSIDRYLAVVHPIKSAKWRRPRTAKMINVAVWGVSLLVILPIMIYA

GLRSNQWGRSSCTINWPGESGAWYTGFIIYAFILGFLVPLTIICLCYLFIIIKVKSSGIR

VGSSKRKKSEKKVTRMVSIVVAVFIFCWLPFYIFNVSSVSVAISPTPALKGMFDFVVVLT

YANSCANPILYAFLSDNFKKSFQNVLCLVKVSGTDDGERSDSKQDKSRLNETTETQRTLL

NGDLQTSI

>Goat SSTR5 Uniprot ID:A0A8C2PGG9

MEPLFLASPLTVWNTSSAVPSGSGDENGTLAGPGPSPGARAVVVPVLYLLVCAVGLGGNA

LVIYVVLRHAKMKTVTNIYILNLAVADVLLMLGLPFVATQNAISYWPFGPVLCRLVMTLD

GINQFTSIFCLTVMSVDRYLAVVHPIRSARWRRPRVAKLASAAVWAFSLVMSLPLVVFAD

IQEGWNTCNLSWPEPVGLWGAVFIIYTSVLGFFGPLLVICLCYVLIVVKLKASGVRVGST

RRRSERKVTRMVVVVVLVFAGCWLPFFIVNIVNLAFALPEEPASAGAYFFVVVLSYANSC

ANPLLYGFLSDNFRQSFRKVLCLRKGYGAGAEDADATEPRPGPSSRLQEATIPMRSCKAN

GLMQTSKL

>Goat SSTR3 Uniprot ID:A0A452G345

MNTPGSLSPVPATSEPGNTSSAWPPDAVLGNASTASSVAGLAVSGILIPLVYLVVCVVGL

LGNSLVIYVVLRQTATPSVTNVYILNLALADELFMLGLPFLAAQNALSYWPFGSLMCRLV

MAVDGINQFTSIFCLTVMSVDRYLAVVHPTRSARWRTAPVARTVSAAVWVASAIVVLPVV

VFSGVPRGMSTCHMQWPEPAAAWRAGFIIYTAALGFFGPLLVICLCYLLIVVKVRSAGRR

VRAPSCQRRRHSERKVTRMVVAVVALFVLCWMPFYVLNIINVVCPLPEEPAFFGLYFLVV

ALPYANSCANPILYGFLSYRFKQGFRRALLRPSRRVQNQEPPVGPPEKTEEEEEDGDGED

RHEGAGKPGDWGEMNGRVNQIIQPGPSGQEQPPSSTTSKEHQFLPQEPSAGEKSDTLSHL

G

>Sheep SSTR5 Uniprot ID:Q8MI04

MEPLFLASPLTVWNTSSAVPSGRPMRMGRWRAGALPGARAVVVPVLYLLVCAVGLGGNAL

VIYVVLRHAKMKTVTNIYILNLAVADVLLMLGLPFVATQNAISYWPFGPVLCRLVMTLDG

INQFTSIFCLTVMSVDRYLAVVHPIRSARWRRPRVAKLASAAVWAFSLVMSLPLVVFADI

QEGWNTCNLTWPEPVGLWGAVFIIYTSVLGFFGPLLVICLCYVLIVVKLKASGVRVGSTR

RRSERKVTRMVVVVVLVFAGCWLPFFIVNIVNLAFALPEEPASAGLYFFVVVLSYANSCA

NPCLYGFLSDNLRQSFRKVLCLRKGYGAGAEDADATEPRPDPSSRLQEATMPIRSCKANG

LMQTSKL

>Sheep SSTR3 Uniprot ID:W5QG61

MNTPGSLSPVPATSEPGNTSSAWPPDAVLGNASTASSAAGLAVSGILIPLVYLVVCVVGL

LGNSLVIYVVLRQTATPSVTNVYILNLALADELFMLGLPFLAAQNALSYWPFGSLMCRLV

MAVDGINQFTSIFCLTVMSVDRYLAVVHPTRSARWRTAPVARTVSAAVWVASAVVVLPVV

VFSGVPRGMSTCHMQWPEPAAAWRAGFIIYTAALGFFGPLLVICLCYLLIVVKVRSGRHS

ERKVTRMVVAVVALFVLCWMPFYVLNIINVVCPLPEEPAFFGLYFLVVALPYANSCANPI

LYGFLSYRFKQGFRRALLRPSRRVQNQEPPVGPPEKTEEEEEDGDGEDQHEGAGKPGGWG

EMNGRVNQIIQPGPSGQERPPSSTTSKEHQFLPQEPSAGEKSDTLHISYL

>Sheep SSTR2 Uniprot ID:W5NPT8

MDLASELNETQPWLTSPFDLNGSVGAANISNQTEPYYDLASNVVLTFIYFVVCIVGLCGN

TLVIYVILRYAKMKTITNIYILNLAIADELFMLGLPFLAMQVALVHWPFGKAICRVVMTV

DGINQFTSIFCLTVMSIDRYLAVVHPIKSAKWRRPRTAKMINVAVWGVSLLVILPIMIYA

GLRSNQWGRSSCTINWPGESGAWYTGFIIYAFILGFLVPLTIICLCYLFIIIKVKSSGIR

VGSSKRKKSEKKVTRMVSIVVAVFIFCWLPFYIFNVSSVSVAISPTPALKGMFDFVVVLT

YANSCANPILYAFLSDNFKKSFQNVLCLVKVSGTDDGERSDSKQDKSRLNETTETQRTLL

NGDLQTSI

>Sheep SSTR1 Uniprot ID:W5Q7Z2

MFPNGTASSPSSPSPSPGSCGEGGGGRGPGAGAADGMEEPGRNASQNGTLSEGQGSAILI

SFIYSVVCLVGLCGNSMVIYVILRYAKMKTATNIYILNLAIADELLMLSVPFLVTSTLLR

HWPFGALLCRLVLSVDAVNMFTSIYCLTVLSVDRYVAVVHPIKAARYRRPTVAKVVNLGV

WVLSLLVILPIVVFSRTAANSDGTVACNMLMPEPAQRWLVGFVLYTFLMGFLLPVGAICL

CYVLIIAKMRMVALKAGWQQRKRSERKITLMVMMVVMVFVICWMPFYVVQLVNVFAEQDD

ATVSQLSVILGYANSCANPILYGFLSDNFKRSFQRILCLSWMDNAAEEPVDYYATALKSR

AYSVEDFQPENLESGGVFRNGTCTSRITTL

>Bovine SSTR2 Uniprot ID:F1MEN6

MDLVSELNETQPWLTAPFDLNGSVGAANISNQTEPYYDLASNVVLTFIYFVVCIIGLCGN

TLVIYVILRYAKMKTITNIYILNLAIADELFMLGLPFLAMQVALVHWPFGKAMCRVVMTV

DGINQFTSIFCLTVMSIDRYLAVVHPIKSAKWRRPRTAKMINVAVWGVSLLVILPIMIYA

GLRSNQWGRSSCTINWPGESGAWYTGFIIYAFILGFLVPLTIICLCYLFIIIKVKSSGIR

VGSSKRKKSEKKVTRMVSIVVAVFIFCWLPFYIFNVSSVSVAISPTPALKGMFDFVVVLT

YANSCANPILYAFLSDNFKKSFQNVLCLVKVSGTDDGERSDSKQDKSRLNETTETQRTLL

NGDLQTSI

>Bovine SSTR5 Uniprot ID:F1MEN6

MEPLFPASPLTTWNTSSVVPSGSGDENGTLAGLGPSPGARAVVVPVLYLLVCAVGLGGNT

LVIYVVLRHAKMKTVTNIYILNLAVADVLLMLGLPFVATQNAISYWPFGPVLCRLVMTLD

GINQFTSIFCLTVMSVDRYLAVVHPIRSARWRRPRVAKLASAAVWAFSLVMSLPLVVFAD

IQEGWNTCNLSWPEPVGLWGAVFIIYTSVLGFFGPLLVICLCYLLIVVKLKASGVRVGST

RRRSERKVTRMVVVVVLVFAGCWLPFFIVNIVNLAFALPEEPASAGAYFFVVVLSYANSC

ANPLLYGFLSDNFRQSFRKVLCLRKGYGAGAEDADATEPQPGPSSRLQEAMMPVRSCKAN

GLMQTSKL

>Bovine SSTR3 Uniprot ID:G3MWT4

MNTPGSLSLLPVTSEPGNTSSAWPPDAVLGNVSAASSAAGLAVSGILIPLVYLVVCVVGL

LGNSLVIYVVLRQTATPSVTNVYILNLALADELFMLGLPFLAAQNALSYWPFGSLMCRLV

MAVDGINQFTSIFCLTVMSVDRYLAVVHPTRSARWRTAPVARTVSAAVWVASAVVVLPVV

VFSGVPRGMSTCHMQWPEPAAAWRAGFIIYTAALGFFGPLLVICLCYLLIVVKVRSAGRR

VRAPSCQRRRHSERKVTRMVVAVVALFVLCWMPFYVLNIINVVCPLPEEPAFFGLYFLVV

ALPYANSCANPILYGFLSYRFKQGFRRVLLRPSRRVQNQEPPVGPPEKTEEEEEAGDGED

RHEGAGKQGDWGEMNGRVNQIIQPGPSGQERPPSSTTSKERQFLPQEPLAGEKSDTLHIS

YL

>Bovine SSTR1 Uniprot ID:E1BFV6

MDFQYRAWHVWMGEGGRAVRLRLLRSQQRVLLSAVAVARQTQGALGTTSCIKRFAVGFLR

APEHSRPPRAACGVIRQKPLTQGCGPSSSSCSRLRVSFRDSSESEWAPVLGSRPKKGEGP

HGERHRAYQTEPALCTGQLRVGLETARPGPSPGSCGEGGGSRGPGAGAADGMEEPGRNAS

QNGTLSEGQGSAILISFIYSVVCLVGLCGNSMVIYVILRYAKMKTATNIYILNLAIADEL

LMLSVPFLVTSTLLRHWPFGALLCRLVLSVDAVNMFTSIYCLTVLSVDRYVAVVHPIKAA

RYRRPTVAKVVNLGVWVLSLLVILPIVVFSRTAANSDGTVACNMLMPEPAQRWLVGFVLY

TFLMGFLLPVGAICLCYVLIIAKMRMVALKAGWQQRKRSERKITLMVMMVVMVFVICWMP

FYVVQLVNVFAEQDDATVSQLSVILGYANSCANPILYGFLSDNFKRSFQRILCLSWMDNA

AEEPVDYYATALKSRAYSVEDFQPENLESGGVFRNGTCTSRITTL

>Bovine SSTR5 Uniprot ID:M5FK46

MEPLFPASPLTTWNTSSVVPSGSGDENGTLAGLGPSPGARAVVVPVLYLLVCAVGLGGNT

LVIYVVLRHAKMKTVTNIYILNLAVADVLLMLGLPFVATQNAISYWPFGPVLCRLVMTLD

GINQFTSIFCLTVMSVDRYLAVVHPIRSARWRRPRVAKLASAAVWAFSLVMSLPLVVFAD

IQEGWNTCNLSWPEPVGLWGAVFIIYTSVLGFFGPLLVICLCYLLIVVKLKASGVRVGST

RRRSERKVTRMVVVVVLVFAGCWLPFFIVNIVNLAFALPEEPASAGAYFFVVVLSYANSC

ANPLLYGFLSDNFRQSFRKVLCLRKGYGAGAEDADATEPQPGPSSRLQEAMMPVRSCKAN

GLMQTSKL

>Macaque SSTR2 Uniprot ID:A0A5F8A2I1

MDMVDKPLNGSHTWLSIPFDLNGSVVSTNTSNQTEPYYDLTSNAVLTFIYFVVCIVGLCG

NTLVIYVILRYAKMKTITNIYILNLAIADELFMLGLPFLAMQVALVHWPFGKAICRVVMT

VDGINQFTSIFCLTVMSIDRYLAVVHPIKSAKWRRPRTAKMITMAVWGVSLLVILPIMIY

AGLRSNQWGRSSCTINWPGESGAWYTGFIIYTFILGFLVPLTIICLCYLFIIIKVKSSGI

RVGSSKRKKSEKKVTRMVSIVVAVFIFCWLPFYIFNVSSVSMAISPTPALKGMFDFVVVL

TYANSCANPILYAFLSDNFKKSFQNVLCLVKVSGTDDGERSDSKQDKSRLNETTETQRTL

LNGDLQTSI

>Macaque SSTR4 Uniprot ID:A0A2K5UGX7

MNAPSTLPPGGEEGLETAWPPAANASSAPAEEEEAVAGCGDAGAAGMVAIQCIYALVCLV

GLVGNALVIFVILRYAKMKTATNIYLLNLAVADELFMLSVFGWPPLRHWPFGSVLCRTVL

SVDGLNMFTSVFCLTVLSVDRYVAVVHPLRAATYRRPSVAKLINLGVWLASLLVTLPIAI

FADTRPARGGQAVACNLHWPHPAWSAVFVVYTFLLGFLLPVLAIGLCYLLIVGKMRAVAL

RAGWQQRRRSEKKITRLVLMVVAVFVLCWMPFYVVQLLNLFVTSLDATINHVSLILSYAN

SCANPVLYGFLSDNFRRSFQRVLCLRCCLLEGTGGAEEEPLDYYATALKSRGGAGWMCPP

LPCQQEPLQPEPSRKRIPLTRTTTF

>Macaque SSTR5 Uniprot ID:A0A1D5R040

MEPLFPASTPSWNASSPGAASGGADNRTLVGPAPSVGARAVLVPVLYLLVCAAGLGGNTL

VIYVVLRFAKMKTVTNIYILNLAVADVLYMLGLPFLATQNAASFWPFGPILCRLVMTLDG

VNQFTSVFCLTVMSVDRYLAVVHPLSSARWRRPRVAKLASAAAWALSLCMSLPLLVFADV

QEGGTCNASWPEPVGLWGAVFIIYTAVLGFFGPLLVICLCYLLIVVKVRAAAVRVGCVRR

RSERKVTRMVLVVVLVFAGCWLPFFTVNIVNLAVALPQEPASAGLYFFVVILSYANSCAN

PVLYGFLSDNFRQSFQKVLCLRKGSGAKDADAMEPRPDRSRQQQEAMPPTHGAKANGLMQ

TSKL

>Macaque SSTR1 Uniprot ID:A0A2K5UEB0

MFPNGTASSPSSSPSPSPGSCGEGGGSRGPGAGAADGMEEPGRNASQNGTLSEGQGSAIL

ISFIYSVVCLVGLCGNSMVIYVILRYAKMKTATNIYILNLAIADELLMLSVPFLVTSTLL

RHWPFGALLCRLVLSVDAVNMFTSIYCLTVLSVDRYVAVVHPIKAARYRRPTVAKVVNLG

VWVLSLLVILPIVVFSRTAANSDGTVACNMLMPEPAQRWLVGFVLYTFLMGFLLPVGAIC

LCYVLIIAKMRMVALKAGWQQRKRSERKITLMVMMVVMVFVICWMPFYVVQLVNVFAEQD

DATVSQLSVILGYANSCANPILYGFLSDNFKRSFQRILCLSWMDNAAEEPVDYYATALKS

RAYSVEDFQPENLESGGVFRNGTCTSRITTL

>Warbler SSTR1 Uniprot ID:A0A7L1FU30

MLPHGTCPRLPGGAGSDSGDSDSGGSDSGGGRAGGASEEAAAEGMDSGGRNSSGAPNSTL

SESQGSAILISFIYSVVCLVGLCGNSMVIYVILRYAKMKTATNIYILNLAIADELLMLSV

PFLVTSTLLHHWPFGSLLCRLVLSVDAINMFTSIYCLTVLSVDRYIAVVHPIKAARYRRP

TVAKMVNLGVWVLSILIILPIIIFSNTAANSDGTVACNMLMPEPTQRWLVVFVVYTFLMG

FLLPVVAICLCYILIIAKMRMVALKAGWQQRKRSERKITLMVMMVVMVFVICWMPFYIVQ

LVNVFVEQDDATISQLSVILGYANSCANPILYGFLSDNFKRSFQRLLCLSWMDNAAEEPI

DYYATALKSRAYSVEDFPPDNLESGSMYRNGTCTSRITTL

>Warbler SSTR4 Uniprot ID:A0A7L1EY11

WASSEPTPNSSTVVAVGEASRPPVSPAHGGSAAEIAGMVVLQCIYGLVCLLGLLGNALVI

FVILRYAKMKTATNIYLLNLAIADELFMLSVPFVATAVALRRWPFGRALCRTVLGVDGLN

MFSSVFCLTVLSLDRYIAVVHPLRAASYRRPRVAKLVNGGVWLLALLVASPIPVFAGTAV

TRDGHAVACDLLWPSPAWAAAFVVYSSTLGFVLPVVAMALCYLLLAGKMRVVAQGVGWQQ

RRRSEGKLTRLVVTVVATFVVCWLPFYVVQLLELLLPGRLDASAHNASLLLSYSNSCANP

ILYGLLSENFRNSFHGVLRRCRDAGLCCCRATDGDGGDSEDEEEPLDYCAAPRGDTKGCV

CPPLPCQQDPLRPQPCCAPGALLPKTTPF

>Warbler SSTR3 Uniprot ID:A0A7L1F516

MDTSAFSLPTPAVLEEGNASSSWAGFTTPNSSSTISPGVVVSGVLIPVVYLIVCVVGLAG

NSLVIYVVLRHSVSESVTNVYILNLALADELFMLGLPFLAAQNALSYWPFGSFMCRLVMA

VDAINQFTSIFCLTVMSVDRYLAVVHPGKSSKWRTARVAKAVSVTMWVLSSIVVLPVVVF

SDVPLGMSTCHIQWPEPASVWRAGFIVYTATLGFFGPLLVICLCYLLIVVKVRSSGRRVR

ALSSKHKLSERRVTRMVVTVVAVFVLCWLPFYVLNIINVVCPLPEEPSLFGVYFLVVVLP

YANSCANPIIYGFLSYRFKQGFRRAIFRPSRRVQSQEVPACFPEKIDDEREEGEISKITQ

NGNDRQEHSLSSGEGKSNQQKPLPEEPVGCEKRNKLHVSYL

>Warbler SSTR5 Uniprot ID:A0A7L1FC20

ANSSLLLLLLPNVTENGTFSEPPPFQYIHKVLIPICYLLVCAVGLSGNALVIYVVLRHAK

MKTVTNIYILNLAVADVLFMLGLPFLATQNAISYWPFGSFLCRLVMTVDGINQFTSIFCL

TVMSMDRYLAVVHPIKSTKWRRPRVAKLISITVWTFSFLVVLPVIIFSDVQEDFQTCNMN

WPEPVNVWSAAFIIYTSALGFFGPLLVICLCYLLIVVKVKSSGIRVGSTRRRRSERKVTR

MVVIIVVVFVFCWLPFYTMNIVNLILILPADPVLEGLYFFMVVLSYANSCANPILYGFLS

DNFKQSFRKVLCLRKGDGPEDGEPVEHRQENSSRLQESMLTQRNVEFNGHMQTSKV

>Warbler SSTR2 Uniprot ID:A0A7L1FHX6

MELEDELPNATAFWFSPASPFDNFSLEAPTNTSQNATGQHFDLTSNAILTFIYFVVCIVG

LCGNTLVIYVILRYAKMKTITNIYILNLAIADELFMLGLPFLAMQVALVHWPFGKALCRI

VMTVDGINQFTSIFCLTVMSVDRYLAVVHPIKSAKWRRPRTAKMINVAVWGVSLLVIMPI

MIYAGVQHNHGRSSCTIIWPGESGAWYTGFIIYAFILGFLVPLTIICLCYLFIIIKVKSS

GIRVGSSKRKKSEKKVTRMVSIVVAVFIFCWLPFYIFNVSSVSVMIVPTPVLKGMFDFVV

VLSYANSCANPILYAFLSDNFKKSFQNVLCLVKVSGMDEADRSDSKQDKSRLNETTETQR

TLLNGDLQTSI

>Duck SSTR1 Uniprot ID:U3IUQ1

MLPNGTCTRLPGGAGSDSGGSDSGGGGAGGALEEAAAGGMDSGGRNSSGAPNSTLSESQG

SAILISFIYSVVCLVGLCGNSMVIYVILRYAKMKTATNIYILNLAIADELLMLSVPFLVT

STLLHHWPFGSLLCRLVLSVDAINMFTSIYCLTVLSVDRYIAVVHPIKAARYRRPTVAKM

VNLGVWVLSILIILPIIIFSNTAANSDGTVACNMLMPEPTQRWLVVFVVYTFLMGFLLPV

VAICLCYILIIAKMRMVALKAGWQQRKRSERKITLMVMMVVMVFVICWMPFYIVQLVNVF

VEQDDATISQLSVILGYANSCANPILYGFLSDNFKRSFQRLLCLSWMDNAAEEPIDYYAT

ALKSRAYSVEDFPPDNLESGSMYRNGTCTSRITTL

> Duck SSTR2 Uniprot ID:U3HZT6

MELEFELPNATAFWFSPASPFDNFSVEAPTNASQNATGQHFDLTSNAILTFIYFVVCIVG

LCGNTLVIYVILRYAKMKTITNIYILNLAIADELFMLGLPFLAMQVALVHWPFGKAICRV

VMTVDGINQFTSIFCLTVMSVDRYLAVVHPIKSAKWRRPRTAKMINVAVWGVSLLVIMPI

MIYAGVQHNHGRSSCTIIWPGESGAWYTGFIIYAFILGFLVPLTIICLCYLFIIIKVKSS

GIRVGSSKRKKSEKKVTRMVSIVVAVFIFCWLPFYIFNVSSVSVLIVPTPVLKGMFDFVV

VLSYANSCANPILYAFLSDNFKKSFQNVLCLVKVSGMDDADRSDSKQDKSRLNETTETQR

TLLNGDLQTSI

> Duck SSTR3 Uniprot ID:U3I0S7

MDTSTFSLPTPAVSEEGNASGSWAGFTTPNSSTTASPGVVFSGVLIPLVYLIVCVVGLVG

NSLVIYVVLRHSVSESVTNVYILNLALADELFMLGLPFLAAQNALSYWPFGSFMCRLVMA

VDAINQFTSIFCLTVMSVDRYLAVVHPGKSSKWRTARVAKAVSATVWMLSSVVVLPVVVF

SDVPLGMSTCHIQWPEPASVWRAGFIVYTATLGFFGPLLVICLCYLLIVVKVRSSGRRVR

ALSSKHKLSERRVTRMVVAVVAVFVLCWLPFYVLNIINVVCPLPEEPSLFGVYFLVVVLP

YANSCANPIIYGFLSYRFKQGFRRAIFRPSRRVQSQEVPAGPPEKTDDEGEEGEISKIAQ

NGNDRQERPLCSGAGESNEQKPLPEQPVGSEKSNKLHVSYL

>Duck SSTR4 Uniprot ID:A0A493SVA4

MAPFRLLHHEFHPGSDFRRGFGRVTALCTLLHQLFARGRALGQGPGQELWQPPPWDVGCV

ARCGMRTRMRDARQDAGASAGGALPPSRRQGNRPPSPPTRGGPPQPPLGAGGSRPSLPRC

WTAASPPPQQAWDGGGWERASLGHGRSKRNAANLKGKGEGFGSCRSSDPGQAGLYLASSP

WSSSPXWLAAPAAAPQQRAGGGLSIPGGLRGWHPSRRCPPPWATPSMNVEAEQLPLWATS

EMPPNTSEASTEAGQLQGPSAWGGRAGEIAGMVVIQCIYALVCLLGLLGNSLVIFVILRY

AKMKTATNIYLLNLAIADELFMLSIPFVATSAALHHWPFGRALCRTVLGVDGLNMFTSVF

CLTVLSLDRYIAVVHPLRAATYRRPRVAKIVNGGVWLLSLLVASPIPIFAGTATTRDGQA

VACNLLWPSPAWSAAFVVYTTLLGFLLPVLAMGLCYLLIVGKMRAVAQRVGWQQRRRSEG

KLTRLVLMVVAMFVVCWMPFYVVQLVNLLLPGRLDATVNNASLILSYSNSCANPILYGFL

SENFRHSFHGVLRRCLDASLCCCHAEAGAAEEEEEEEEPLDYCAVPRGDDKGKGCMCPSL

PCQQEPAHPEPCCKPGTLLAKTTTF

> Duck SSTR5 Uniprot ID:U3I1T8

MDPLYFPSTLSMDASSSDLNSSLLTNVTENGTLSEQPPFKYIHKVLIPIFYILVCAVGLS

GNTLVIYVVLRYAKMKTVTNIYILNLAIADVLFMLGLPFLATQNAISYWPFGSFLCRLVM

TVDGINQFTSIFCLTVMSMDRYLAVVHPIKSTKWRRPRVAKLISMTVWTFSFLVVLPVFI

FSDVQEDFHTCNMNWPEPVNIWSAAFIIYTSVLGFFGPLLVICLCYLLIVIKVKSSGIRV

GSTRRRRSERKVTRMVVIIVVVFVFCWLPFYMMNIVNLIFILPEDPVLVGVYFFVVVLSY

ANSCANPILYGFLSDNFKQSFQKVLCLRKGNGVEDGDPIEHRQENSSRLQESMLTQRNIE

FNGHMQTSKV

>Ma's night monkey SSTR2 Uniprot ID:A0A2K5C5L2

TDMADEPLNGSHTWLSIPFDLNGSVGSANTSNQTEPYYDLTSNAVLTFIYFVVCIIGLCG

NTLVIYVILRYAKMKTITNIYILNLAIADELFMLGLPFLAMQVALVHWPFGKAICRVVMT

VDGINQFTSIFCLTVMSIDRYLAVVHPIKSAKWRRPRTAKMITMAVWGVSLLVILPIMIY

AGLRSNQWGRSSCTINWPGESGAWYTGFIIYTFILGFLVPLTIICLCYLFIIIKVKSSGI

RVGSSKRKKSEKKVTRMVSIVVAVFIFCWLPFYIFNVSSVSMAISPTPALKGMFDFVVVL

TYANSCANPILYAFLSDNFKKSFQNVLCLVKVSGTDDGERSDSKQDKSRLNETTETQRTL

LNGDLQTSI

>Ma's night monkey SSTR3 Uniprot ID:A0A2K5E308

MDTLHPSPVSTTSEPENASSAWPPDATLGNVSAGPSSAGLAVSGVLIPLVYLVVCVVGLL

GNSLVIYVVLRHTASPSVTNVYILNLALADELFMLGLPFLAAQNALSYWPFGSLMCRLVM

AVDGINQFTSIFCLTVMSVDRYLAVVHPTRSARWRTAPVARMVSAAVWVASAVVVLPVVV

FSGVPRGMSTCHMQWPEPAAAWRAGFIIYTAALGFFGPLLVICLCYLLIVVKVRSAGRRV

WAPSCQRRRRSERRVTRMVVAVVALFVLCWMPFYVLNIVNVVCPLPEEPAFFGLYFLVVA

LPYANSCANPILYGFLSYRFKQGFRRVLLRPSRRVCSQEPTAGPPEKTEEEDEEEEDGEG

SRERGKGKEMNGRVSQITQPRTSGQERPPSRAASKEQQFLPQEPSTGEKSSTRHTSYL

>Ma's night monkey SSTR1 Uniprot ID:A0A2K5C1A3

MFPNGTASSPSSSPSPSPGSCCEGGGSRGPGAGAADGMEEPGRNASQNGTLSEGQGSAIL

ISFIYSVVCLVGLCGNSMVIYVILRYAKMKTATNIYILNLAIADELLMLSVPFLVTSTLL

HHWPFGALLCRLVLSVDAVNMFTSIYCLTVLSVDRYVAVVHPIKAARYRRPTVAKVVNLG

VWVLSLLVILPIVVFSRTAANSDGTVACNMLMPEPAQRWLVGFVLYTFLMGFLLPVGAIC

LCYVLIIAKMRMVALKAGWQQRKRSERKITLMVMMVVMVFVICWMPFYVVQLVNVFAEQD

DATVSQLSVILGYANSCANPILYGFLSDNFKRSFQRILCLSWMDNAAEEPVDYYATALKS

RAYSVEDFQPENLESGGVFRNGTCTSRITTL

>Ma's night monkey SSTR4 Uniprot ID:A0A2K5E039

MSAPSTLPPGGEEGLETAWPPAANTSSAPAEEEEAVAGRGGAGAAGMVAIQCIYALVCLV

GLVGNALVIFVILRYAKMKTATNIYLLNLAVADELFMLSVPFVASSAALRHWPFGSVLCR

AVLSVDGLNMFTSVFCLTVLSVDRYVAVVHPLRAATYRRPSVAKLINLGVWLASLLVTLP

IAIFADTRPARGGQAVACNLHWPHPAWSAVFVVYTFLLGFLLPVLAIGLCYLLIVGKMRA

VALRAGWQQRRRSEKKITRLVLMVVAVFVLCWMPFYVVQLLNLFVTSLDATVNHVSLILS

YANSCANPILYGFLSDNFRRSFQRVLCLRCCLLEGAGGAEEEPLDYYATALKSRGGAGCI

CPPLPCQQEPLQPEPSRKRIPFTRTTTF

>Rat SSTR1 Uniprot ID:P28646

MFPNGTAPSPTSSPSSSPGGCGEGVCSRGPGSGAADGMEEPGRNSSQNGTLSEGQGSAIL

ISFIYSVVCLVGLCGNSMVIYVILRYAKMKTATNIYILNLAIADELLMLSVPFLVTSTLL

RHWPFGALLCRLVLSVDAVNMFTSIYCLTVLSVDRYVAVVHPIKAARYRRPTVAKVVNLG

VWVLSLLVILPIVVFSRTAANSDGTVACNMLMPEPAQRWLVGFVLYTFLMGFLLPVGAIC

LCYVLIIAKMRMVALKAGWQQRKRSERKITLMVMMVVMVFVICWMPFYVVQLVNVFAEQD

DATVSQLSVILGYANSCANPILYGFLSDNFKRSFQRILCLSWMDNAAEEPVDYYATALKS

RAYSVEDFQPENLESGGVFRNGTCASRISTL

>Rat SSTR3 Uniprot ID:P30936

MAAVTYPSSVPTTLDPGNASSAWPLDTSLGNASAGTSLAGLAVSGILISLVYLVVCVVGL

LGNSLVIYVVLRHTSSPSVTSVYILNLALADELFMLGLPFLAAQNALSYWPFGSLMCRLV

MAVDGINQFTSIFCLTVMSVDRYLAVVHPTRSARWRTAPVARMVSAAVWVASAVVVLPVV

VFSGVPRGMSTCHMQWPEPAAAWRTAFIIYTAALGFFGPLLVICLCYLLIVVKVRSTTRR

VRAPSCQWVQAPACQRRRRSERRVTRMVVAVVALFVLCWMPFYLLNIVNVVCPLPEEPAF

FGLYFLVVALPYANSCANPILYGFLSYRFKQGFRRILLRPSRRVRSQEPGSGPPEKTEEE

EDEEEEERREEEERRMQRGQEMNGRLSQIAQPGPSGQQQRPCTGTAKEQQLLPQEATAGD

KASTLSHL

>Rat SSTR4 Uniprot ID:P30937

MNTPATLPLGGEDTTWTPGINASWAPDEEEDAVRSDGTGTAGMVTIQCIYALVCLVGLVG

NALVIFVILRYAKMKTATNIYLLNLAVADELFMLSVPFVASAAALRHWPFGAVLCRAVLS

VDGLNMFTSVFCLTVLSVDRYVAVVHPLRAATYRRPSVAKLINLGVWLASLLVTLPIAVF

ADTRPARGGEAVACNLHWPHPAWSAVFVIYTFLLGFLLPVLAIGLCYLLIVGKMRAVALR

AGWQQRRRSEKKITRLVLMVVTVFVLCWMPFYVVQLLNLFVTSLDATVNHVSLILSYANS

CANPILYGFLSDNFRRSFQRVLCLRCCLLETTGGAEEEPLDYYATALKSRGGPGCICPPL

PCQQEPMQAEPACKRVPFTKTTTF

>Rat SSTR5_ P30938

MEPLSLASTPSWNASAASSGNHNWSLVGSASPMGARAVLVPVLYLLVCTVGLSGNTLVIY

VVLRHAKMKTVTNVYILNLAVADVLFMLGLPFLATQNAVVSYWPFGSFLCRLVMTLDGIN

QFTSIFCLMVMSVDRYLAVVHPLRSARWRRPRVAKMASAAVWVFSLLMSLPLLVFADVQE

GWGTCNLSWPEPVGLWGAAFITYTSVLGFFGPLLVICLCYLLIVVKVKAAGMRVGSSRRR

RSEPKVTRMVVVVVLVFVGCWLPFFIVNIVNLAFTLPEEPTSAGLYFFVVVLSYANSCAN

PLLYGFLSDNFRQSFRKVLCLRRGYGMEDADAIEPRPDKSGRPQATLPTRSCEANGLMQT

SRI

>Rat SSTR2_ P30680

MELTSEQFNGSQVWIPSPFDLNGSLGPSNGSNQTEPYYDMTSNAVLTFIYFVVCVVGLCG

NTLVIYVILRYAKMKTITNIYILNLAIADELFMLGLPFLAMQVALVHWPFGKAICRVVMT

VDGINQFTSIFCLTVMSIDRYLAVVHPIKSAKWRRPRTAKMINVAVWGVSLLVILPIMIY

AGLRSNQWGRSSCTINWPGESGAWYTGFIIYAFILGFLVPLTIICLCYLFIIIKVKSSGI

RVGSSKRKKSEKKVTRMVSIVVAVFIFCWLPFYIFNVSSVSVAISPTPALKGMFDFVVIL

TYANSCANPILYAFLSDNFKKSFQNVLCLVKVSGAEDGERSDSKQDKSRLNETTETQRTL

LNGDLQTSI

>Cat SSTR4 Uniprot ID:M3X0A6

MSAPPTLPPEGEEELQTAWSPAVNASCAPAEEEAAAGTGDAGAPGMVAIQCIYALVCLVG

LVGNALVIFVILRYAKMKTATNIYLLNLAIADELFMLSVPFVASSAALRHWPFGSVLCRA

VLSVDGLNMFTSVFCLTVLSVDRYVAVVHPLRAATYRRPSVAKLINLGVWLASLLVTLPI

AIFADTKPARGGQAVACNLHWPHPAWSAVFVIYTFLLGFLLPVLAIGLCYLLIVGKMRAV

ALRAGWQQRRRSEKKITRLVLMVVAVFVLCWMPFYVVQLLNLFVTSLDATVNHVSLILSY

ANSCANPILYGFLSDNFRRSFQRVLCLRCCLLDVAGGAEEEPLDYYATAVKSRGGAGWIC

PPLPCQQEPLRPEPSRKPIPLTRTTTF

>Cat SSTR2 Uniprot ID:M3W2Q8

MDMEYELLNESRPWLSPPFDLNGSVVAANGSNQTEPYYDLTSNAVLTFIYFVVCIIGLCG

NTLVIYVILRYAKMKTITNIYILNLAIADELFMLGLPFLAMQVALVHWPFGKAICRVVMT

VDGINQFTSIFCLTVMSIDRYLAVVHPIKSAKWRRPRTAKMVNVAVWGVSLLVILPIMIY

AGLRSNQWGRSSCTINWPGESGAWYTGFIIYTFILGFLVPLTIICLCYLFIIIKVKSSGI

RVGSSKRKKSEKKVTRMVSIVVAVFIFCWLPFYIFNVSSVSVAINPTPALKGMFDFVVVL

TYANSCANPILYAFLSDNFKKSFQNVLCLVKVSGTDDGERSDSKQDKSRLNETTETQRTL

LNGDLQTSI

>Cat SSTR5 Uniprot ID:M3X0A6

MEPLFPVPTLAAWNASAAAAPGAGGHNGTLAGPAASPGARAVVVPVLYLLVCVVGLGGNA

LVIYVVLRHAKMKTVTNIYILNLAVADVLLMLGLPFLATQNAVSYWPFGPVLCRLVMTLD

GINQFTSVFCLTVMSVDRYLAVVHPIRSARWRRPRVAKLASAGVWTFSLLMSLPLVVFAD

IQEGWDTCNVSWPEPVGLWGAVFIIYTSVLGFFGPLLVICLCYLLIVVKVKASGVRVGAT

RRRSERKATRMVVVVVVVFAGCWLPFFIVNIVNLAFVLPAEPASAGAYFFVVVLSYANSC

ANPVLYGFLSDNFRQSFRKVLCLRKGYGAEDAEAAEPQADKSGRLREATTPARGSEANGL

MQTSRL

>Cat SSTR1 Uniprot ID:M3WBJ6

MFPNGTASSPSSPSPSPGSCGEGGGSRGPGAGAAGGMEEPGRNASQNGTLSEGQGSAILI

SFIYSVVCLVGLCGNSMVIYVILRYAKMKTATNIYILNLAIADELLMLSVPFLVTSTLLR

HWPFGALLCRLVLSVDAVNMFTSIYCLTVLSVDRYVAVVHPIKAARYRRPTVAKVVNLGV

WVLSLLVILPIVVFSRTAANSDGTVACNMLMPEPAQRWLVGFVLYTFLMGFLLPVGAICL

CYVLIIAKMRMVALKAGWQQRKRSERKITLMVMMVVMVFVICWMPFYVVQLVNVFAEQDD

ATVSQLSVILGYANSCANPILYGFLSDNFKRSFQRILCLSWMDNAAEEPVDYYATALKSR

AYSVEDFQPENLESGGGVFRNGTCTSRITTL
